# Nonenzymatic Hydration of Phosphoenolpyruvate: General Conditions for Hydration in Protometabolism by Searching Across Pathways

**DOI:** 10.1002/anie.202410698

**Published:** 2024-12-02

**Authors:** Joris Zimmermann, Atalay Bora Basar, Joseph Moran

**Affiliations:** ^1^ Institut de Science et d'Ingénierie Supramoléculaires (ISIS), CNRS UMR 7006 Université de Strasbourg 8 Allée Gaspard Monge 67000 Strasbourg France; ^2^ Department of Chemistry and Biomolecular Sciences University of Ottawa Ottawa Ontario K1 N 6 N5 Canada

**Keywords:** Protometabolism, Alkene hydration, Phosphoenolpyruvate, Prebiotic Chemistry, Key Mechanisms

## Abstract

Numerous reactions within metabolic pathways have been reported to occur nonenzymatically, supporting the hypothesis that life arose upon a primitive nonenzymatic precursor to metabolism. However, most of those studies reproduce individual transformations or segments of pathways without providing a common set of conditions for classes of reactions that span multiple pathways. In this study, we search across pathways for common nonenzymatic conditions for a recurring chemical transformation in metabolism: alkene hydration. The mild conditions that we identify (Fe oxides such as green rust) apply to all hydration reactions of the rTCA cycle and gluconeogenesis, including the hydration of phosphoenolpyruvate (PEP) to 2‐phosphoglycerate (2PGA), which had not previously been reported under nonenzymatic conditions. Mechanistic insights were obtained by studying analogous substrates and through anoxic and radical trapping experiments. Searching for nonenzymatic conditions across pathways provides a complementary strategy to triangulate conditions conducive to the nonenzymatic emergence of a protometabolism.

## Graphical abstract

By using a novel search strategy, we identified common mild nonenzymatic conditions for all hydration reactions in gluconeogenesis and the rTCA cycle and for the retro‐aldol cleavage of citrate, advancing the search for an environment conducive to the nonenzymatic emergence of a protometabolism.

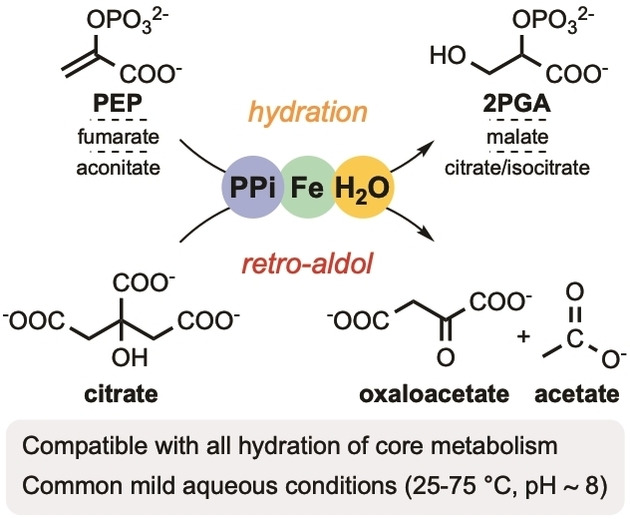

## Introduction

Life is a complex chemical system and identifying the processes leading to its emergence is one of the most challenging problems in science. Self‐organized complex systems are created when a specific group of repeating mechanisms interact with each other. The “metabolism‐first hypothesis” is an approach to understanding the origin of life[^1^, ^2^, ^3^, ^4^, ^5^, ^6^, ^7^, ^8^, ^9^, ^10^] that suggests that the initial stage of life's development involved a complex network of self‐organized chemical reactions made up of repeating chemical mechanisms that were driven by geological processes on the early Earth. Indeed, biological metabolism features a repeating set of chemical mechanisms, consistent with this idea. As the number of mechanisms found in the core of metabolism is restrained, and since many other downstream functions depend on their continuous operation, the extent to which the core of metabolism might change appears to be highly limited. Indeed, comparing modern autotrophic microbial metabolisms with those inferred to be operating in the last universal common ancestor (LUCA) indicates very little change to the structure and mechanisms of metabolism over roughly 4 billion years.^[10]^ For the same reasons, the main chemical mechanisms and reactions found in the core of metabolism may bear strong similarities to those in the original prebiotic network.

Towards the goal of recreating the origin of life in the lab within the metabolism‐first framework, our group is attempting to infer the environment under which self‐organized chemistry began by systematically searching for conditions under which the most conserved parts of the anabolic network occur in the absence of enzymes. Over the past decade, we and others have reported numerous reactions within metabolic pathways that occur in the presence of inorganic promoters.[^11^, ^12^, ^13^, ^14^, ^15^, ^16^, ^17^, ^18^, ^19^, ^20^, ^21^, ^22^, ^23^, ^24^, ^25^, ^26^, ^27^] However, most of these studies reproduce individual transformations or reaction segments within a single pathway without providing a common set of conditions for classes of reactions that span multiple pathways. Searching across metabolic pathways for conditions that enable a recurring chemical mechanism would offer a complementary search strategy that could help pinpoint conditions of broader relevance to the origins of metabolism.

**Figure 1:**
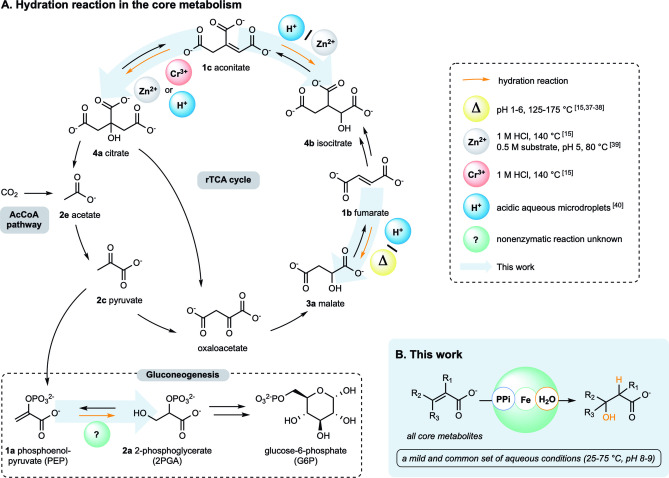
(A) Hydration reactions in core metabolism and the different reports of this nonenzymatic transformation, notably lacking the hydration of **PEP** to **2PGA**. (B) This work, in which a common set of mild aqueous conditions is suitable for the hydration of all core metabolites in the Figure.

Alkene hydration (and its mechanistic reverse, alcohol dehydration) is a reaction mechanism that occurs three times within the rTCA cycle (known as the reverse Krebs cycle),^[28]^ an autocatalytic pathway that produces life's universal organic building blocks for biosynthesis^[29]^ (Figure 1A). Within this cycle, fumarate is hydrated to malate whereas aconitate undergoes hydration to citrate or to isocitrate. The hydration reactions converting fumarate (**1 b**) to malate (**3 a**) and, aconitate (**1 c**) to citrate (**4 a**) and isocitrate (**4 b**) are thermodynamically favorable (−3.57, −8.49, and −2.38 kJ/mol, respectively^[30]^) and involve an iron‐sulfur cluster as a co‐factor.[^31^, ^32^] The mechanism proceeds through the *anti*‐addition of a water molecule across the double bond of fumarate or aconitate by fumarase or aconitase, respectively.^[33]^ Alkene hydration also occurs once within gluconeogenesis, the pathway that life uses to build the sugar‐phosphate backbone that becomes incorporated into nucleic acids. The alkene within the enol moiety of phosphoenolpyruvate (**PEP**, **1 a**) undergoes hydration to form 2‐phosphoglycerate (**2PGA**, **2 a**).^[34]^ Unlike the hydration reactions in the rTCA cycle, the biological hydration of **PEP** to **2PGA** is not thermodynamically favorable (+2.8 kJ/mol^[35]^) and does not involve a Lewis acid Fe−S cluster; instead, Mg^2+^ catalysis is used.[^34^, ^36^]

Some of the metabolic hydration reactions described above have been reported to occur under nonenzymatic conditions, albeit under quite different ones, whereas the hydration of **PEP** to **2PGA** has not yet been reported (Figure 1A). For example, the hydration of fumarate to malate was found to occur in acidic conditions (pH 1–6) at elevated temperatures (125–200 °C) without metal catalysts.[^15^, ^37^, ^38^] The conversion of aconitate to isocitrate was promoted by Zn^2+^ ions in highly acidic conditions (1 M HCl) at elevated temperature (140 °C)^[15]^ or in concentrated and acidic conditions (0.5 M substrate, pH 5) at 80 °C.^[39]^ Similarly, the conversion of aconitate to citrate was shown to proceed in highly acidic conditions (1 M HCl) under Cr^3+^ catalysis at elevated temperatures (140 °C).^[15]^ Alternatively, aqueous microdroplets generated under high‐pressure nebulization were found to hydrate fumarate to malate and to hydrate aconitate to a mixture of isocitrate and citrate at the air–water interface,^[40]^ which was found to be highly acidic.^[41]^ However, highly acidic conditions, especially those rich in metal ions, are not compatible with the hydration of **PEP** to **2PGA** since **PEP** readily hydrolyses to pyruvate under those conditions.[^42^, ^43^, ^44^] Here, we search across pathways for common nonenzymatic conditions for alkene hydration and identify mild, unified conditions that apply to all hydration reactions of the rTCA cycle and gluconeogenesis (Figure 1B), including, for the first time, the hydration of **PEP** to **2PGA**. Interestingly, the conditions empirically identified through a broad screen are environments previously predicted to be of high interest for the origin of metabolism (mildly alkaline aqueous conditions containing Fe oxides such as green rust).[^3^, ^45^] These results help constrain the search for conditions for the emergence of a nonenzymatic metabolism‐like reaction network.

## Results and Discussion

### Nonenzymatic Hydration of Phosphoenolpyruvate

**Figure 2:**
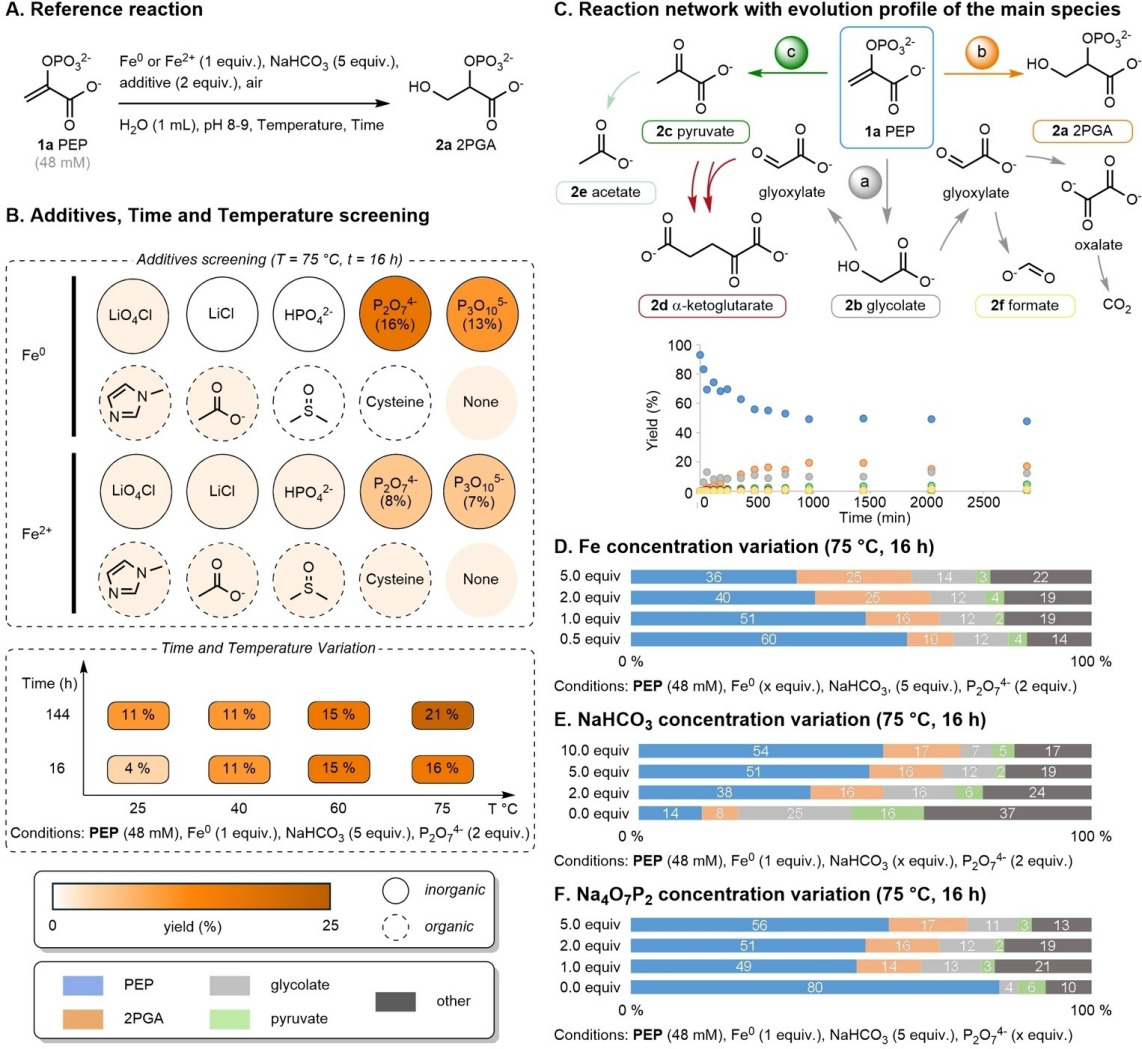
(A) Reference reaction. (B) Additives, time, and temperature screening. (C) Reaction network and the associated quantitative evolution of the main species in the reaction (for details, see Table S7). Concentration variations with the quantification of the main species, including **1 a** (blue), **2 a** (orange), **2 b** (grey), and **2 c** (green) in the reaction with unaccounted mass balance given in black with the component varied: (D) Fe, (E) NaHCO_3_ and (F) Na_4_O_7_P_2_.

We began our investigation by screening for conditions that allowed for the nonenzymatic hydration of **PEP** to **2PGA**. **PEP** (**1 a**) hydrolyzes to pyruvate (**2 c**) under acidic conditions (pH 1–7),^[42]^ and thus alkaline pH values were expected to be more suitable. We screened a series of metals using NaHCO_3_ to buffer the reaction at pH 8–9 under air for 24 h at 60 °C (for details see Table S1 and Figure S10), quenching the reaction by precipitating metals with a thiolate/phosphate solution prior to ^1^H NMR analysis. Of the 15 combinations of metals in various oxidation states that were screened, characteristic signals of **2PGA** (**2 a**) were observed in trace amounts only when iron metal or ferrous iron salts were used (Figure S12). Notably, Cr^3+^ or Zn^2+^, which are known to catalyze nonenzymatic hydration reactions under acidic conditions,^[15],[39][46,47]^ or Fe^3+^ were ineffective. We next evaluated the influence of organic and inorganic ligands on the iron metal (Fe^0^) or iron (II) chloride (Fe^2+^) promoted reaction, screening at 75 °C for 16 h (Figure 2A–B, for details, see Table S2). Polyphosphates significantly improved the formation of **2 a** (Figure 2B). Most notably, starting the reaction with Fe^0^ (1 equiv.) and pyrophosphate (2 equiv.), **2 a** was formed in 16 % yield, as determined by quantitative ^1^H NMR (for details, see Table S2). No reaction was observed when pyrophosphate was used in the absence of Fe^0^. When starting the reaction from Fe^2+^ (1 equiv.) and pyrophosphate (2 equiv.), **2 a** was formed in 8 % yield. Without polyphosphates or in the presence of any other organic or inorganic ligands, yields <2 % were obtained (Figure 2B, left) and significant amounts of precipitated iron salts were observed at the end of the reaction (Table S2). Phosphate ligands possess a high affinity for iron ions[^48^, ^49^] and polyphosphates are known to prevent iron precipitation,[^50^, ^51^, ^52^] for example by ligating iron ions released from the oxidation of Fe^0^.^[50]^

We noticed that the conditions that promoted the hydration reaction were very similar to those needed for the formation of green rust. In NaHCO_3_ solutions, green rust can be formed from metallic iron (Fe^0^) in a deep‐green homogeneous layer that covers the metallic surface.^[53]^ A chloride green rust sample was therefore independently prepared from a mixture of Fe^2+^ and Fe^3+^ and tested in our standard conditions (under air, 75 °C, 16 h) to verify that the reaction could occur in the presence of such naturally abundant minerals. In this case, the formation of **2 a** was detected in up to 10 % yield in the presence of pyrophosphate (for details, see Table S8 entry 7–8). Further experiments related to green rust are described in the next section. For subsequent investigation, conditions starting from Fe^0^ were employed since these gave the best yields of **2 a**.

To gain further insight into the mass balance and mechanism, a series of identical reactions were quenched at different times by precipitating metals using a thiolate/phosphate solution and examined by ^1^H and ^31^P NMR (for details, see Table S7). Three main products were identified after quenching the reaction: **2 a**, glycolate (**2 b**), and pyruvate (**2 c**) (Figure 2C). During the first hour of the reaction, mostly **2 b** is formed (13 % yield). After a 1 h induction period, **2 a** starts to form over 16 h, accompanied by a corresponding decrease in the concentration of **1 a** (50 % of **1 a** recovered, Figure 2C). Along with these two main pathways, we identified the formation of a few additional minor species like α‐ketoglutarate (**2 d**), acetate (**2 e**), and formate (**2 f**) (Figure 2C, for details, see Table S7). To understand the mechanism of formation of these side products, we re‐subjected **2 a**, **2 b**, and **2 c** to the standard conditions (i.e., **Fe^0^** (1 equiv.), Na_4_P_2_O_7_ (2 equiv.), and NaHCO_3_ (5 equiv.)). Starting from either of these three compounds, only trace amounts of **2 e** and **2 f** were detected (Figure S17–19). When the reaction was started from **2 b**, it was found to partially oxidize to glyoxylate (Figure 2C, see also Figure S18). **2 d** was recently reported to form through a reductive aldol condensation between **2 c** and glyoxylate.[^18^, ^54^, ^55^] Therefore, the formation of **2 d** is explained by the in situ formation of glyoxylate and **2 c** from **2 b** and **1 a**, respectively (Figure S20).

To better understand this new transformation and its limitations, we systematically evaluated the influence of various parameters on the hydration reaction (i.e., temperature, time, pH, and concentration). Our goal was not to optimize the alkene hydration, but rather to map the spectrum of reaction conditions, which is a key aspect of understanding and developing a protometabolic network. The reaction was found to occur between 25 to 75 °C (Figure 2B, right, for details, see Table S4), at substrate concentration as low as 10 mM (Table S5 entry 11), and at pH values higher than 6 (Table S6).

We next investigated how the different reaction parameters could influence the formation of the main products (Figure 2D–F, for details, see Table S5). The quantification of the main species, including **1 a** (blue), **2 a** (orange), **2 b** (grey), and **2 c** (green), is represented in Figure 2D–F, with the unaccounted mass balance given in black. When the concentration of Fe^0^ was doubled from 1 to 2 equiv., the yield of **2 a** increased from 16 % to 25 % but without a further notable improvement when 5 equiv. of Fe^0^ was used (Figure 2D). Notably, increasing the equiv. of Fe^0^ increased the yield of **2 a** but not of **2 b** or **2 c**. However, when the amount of Fe^0^ was decreased to 0.5 equiv., the yield of **2 a** was also decreased to 10 %. The formation of **2 a** appears linked to the equiv. of Fe^0^, whereas **2 b** and **2 c** are not. Increasing the concentration of NaHCO_3_ did not significantly affect the reaction whereas decreasing the amount of NaHCO_3_ from 5 to 2 to 0 equiv. considerably increased the yields of side products **2 c** (increasing from 3–4 % to 16 %) and **2 b** (increasing from 12 % to 25 %) while decreasing the yield of **2 a** (decreasing from 16 % down to 8 %) (Figure 2E). Furthermore, we observed a significant decrease of the discernable mass balance. These results show that bicarbonate favors the formation of **2 a** over the side products while limiting adsorption on the surface of the metal (for details, see Supporting Information p. S24). We evaluated the importance of the Na_4_O_7_P_2_ concentration (Figure 2F). As previously mentioned, by removing Na_4_O_7_P_2_ from the reaction conditions, the yield of **2 a** was decreased to trace amounts as well as lower yields of **2 b**. However, decreasing the equivalents of Na_4_O_7_P_2_ from 2 to 1 or increasing from 2 to 5 had little influence on the outcome. We note that in Figures 2D–F, the missing mass balance (10–37 %) is accounted for by the other side products in Figure 2C and by the unavoidable loss of metabolites within precipitates or on surfaces (for details, see Table S7).

### Investigation of the Promoter of the Reaction

**Figure 3:**
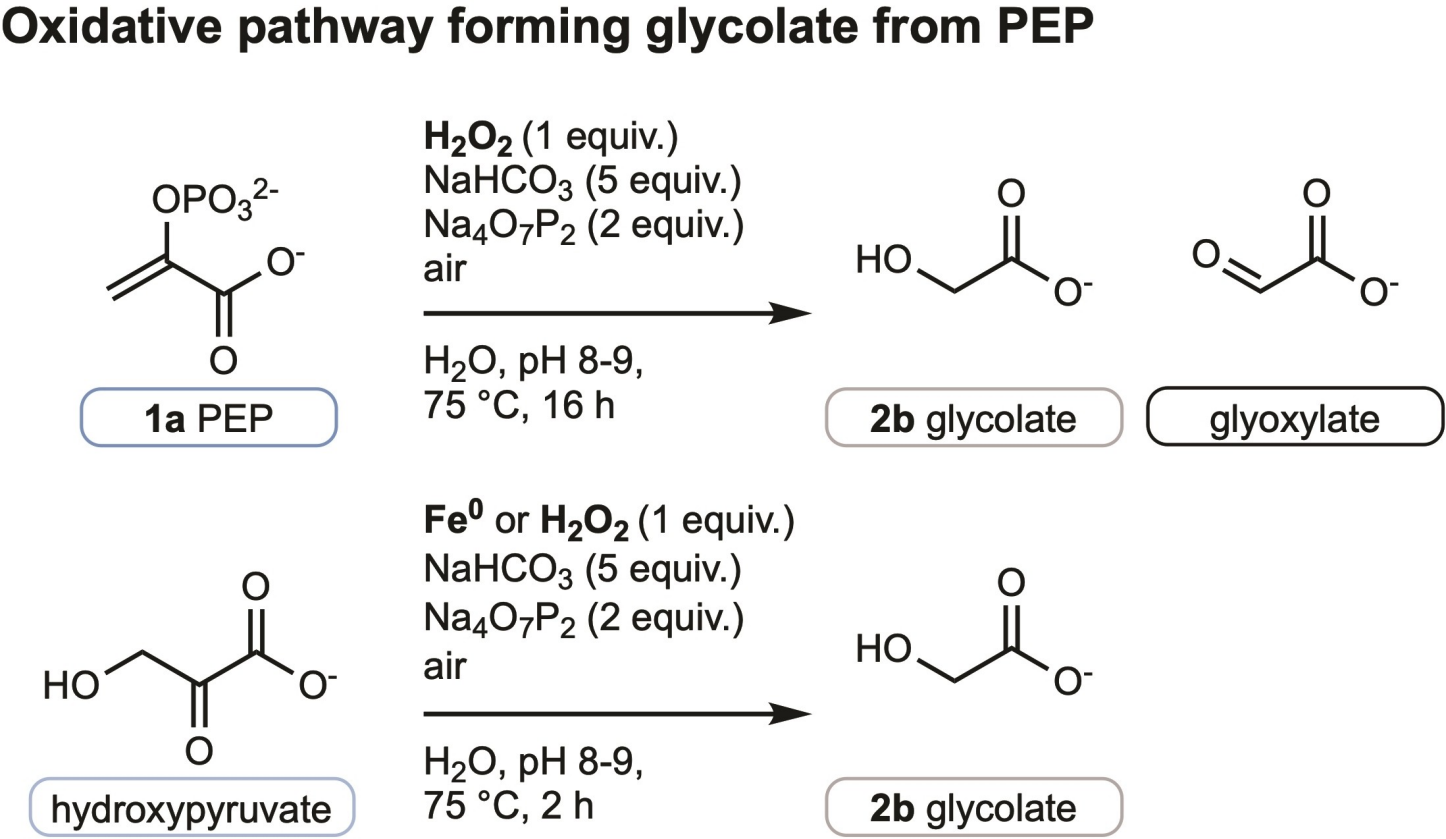
Investigation of the formation of glycolate (**2 b**). Two control experiments were performed in which **2 b** is formed.

The observations that 1) **2 b** is immediately formed in the reaction, 2) **2 a** only begins to be formed after an induction period of approximately 1 h, and 3) **2 a** and **2 b** are not derived from each other suggest that their formation from **1 a** follows two independent mechanisms. As all reactions were thus far carried out under air, we wondered whether O_2_ reduction might be involved in the formation of one of both products. Indeed, in the presence of iron species, O_2_ can be reduced to H_2_O_2_ forming iron oxides in solution which can further form hydroxyl radicals via the Fenton reaction.[^56^, ^57^, ^58^, ^59^] Since the formation of **2 b** was previously reported from the degradation of **1 a** under a variety of oxidative conditions,^[60]^ we first tried mixing **1 a** with H_2_O_2_ (1 equiv.) in the absence of metal under our standard conditions and indeed observed the formation of **2 b** but not **2 a** (Figure 3, see also Figure S21).

Given reports of epoxidation of alkenes by H_2_O_2_ in NaHCO_3_ solution,^[61]^ we propose that epoxidation of **1 a** is followed by hydrolysis of the epoxide^[62]^ and subsequent elimination of phosphate to form hydroxypyruvate, which undergoes oxidative decarboxylation to give **2 b** (Figure S22B). To verify this hypothesis, hydroxypyruvate was subjected to the oxidative reaction conditions and the standard conditions using Fe^0^. In both cases, **2 b** was formed after 2 h at 75 °C (Figure 3, for details, see Figure S22A). Product **2 b** therefore appears to be a direct result of O_2_ reduction under the reaction conditions.

To get more insights and define key parameters regarding the formation of **2 a**, additional experiments were performed. In the lab, green rust can be produced from the oxidation of metallic iron, by the oxidation of Fe(II) species, or by mixing Fe(II) and Fe(III) salts. Carbonate green rust oxidizes into α‐FeO(OH) (goethite) or transforms into Fe_3_O_4_ (magnetite) when left in solution.[^63^, ^64^] We tested the influence of α‐FeO(OH) or Fe_3_O_4_ on the reaction but did not observe the formation of **2 a** in either case (Table S8, entries 9–10). Starting the reaction with Fe^3+^ was ineffective, whereas the addition of a reducing agent such as Zn^0^ in the same mixture promoted the formation of **2 a** in 5 % yield (Table S8, entry 2), highlighting the need for iron oxides of mixed oxidation state generated under air. When Zn^0^ was added under our standard conditions starting from Fe^0^ or Fe^2+^, we observed an increase of yield of **2 a** from 16 % and 8 % to 20 % and 11 %, respectively (Table S8, entries 5 and 11). This suggests that Zn^0^ acts as a reducing agent to regenerate active iron species from overoxidized inactive iron species. Therefore, the overoxidation of iron in solution could be the reason for the loss of reactivity after 1000 min (cf. Figure 2C). Moreover, the source of protons at the C2‐position of **2 a** was verified by performing the reaction in D_2_O, in which case deuterium incorporation was indeed observed (Figure S34). Kinetic experiments were performed to further investigate the mechanism, however, without conclusive results due to the heterogeneous character of the reaction (for details, see SI, section VII, p. S62–66).

**Figure 4:**
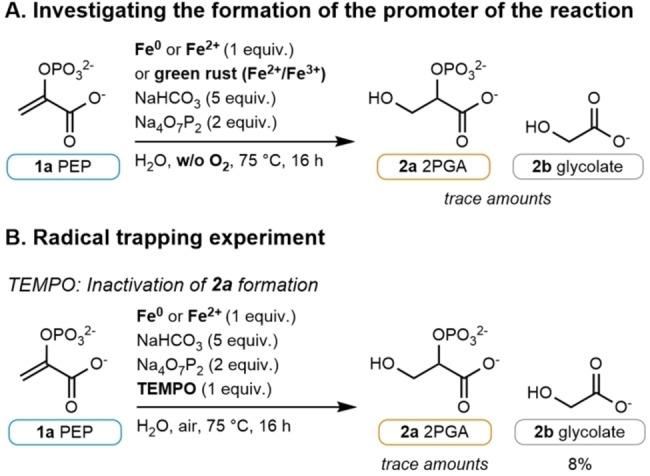
(A) Investigating the formation of the active promoter of the reaction under an inert nitrogen atmosphere. (B) Radical trapping experiments using TEMPO. Quantification of the products by ^1^H qNMR (for details, see Table S10).

To investigate whether O_2_ is involved in the formation of the active promoter, the reaction was carried out starting from Fe^0^, from Fe^2+^, or from preformed green rust in an anaerobic environment (Figure 4A, see Table S9). In the case of Fe^0^ or Fe^2+^, the experiments only produced **2 a** and **2 b** in trace amounts while no product was observed in the case of green rust.

To investigate whether radical species (e.g., derived from the Fenton reaction) are involved in the formation of **2 a**, we performed the reaction under air in the presence of the radical scavenger 2,2,6,6‐tetramethylpiperidinyloxy (TEMPO, 1 equiv.). In this case, only trace amounts of **2 a** were detected while **2 b** was still formed in 8 % yield (Figure 4B, see Table S10). This experiment suggests that an open‐shell species is involved in the formation of **2 a**. Considering that **2 b** forms immediately in the presence of H_2_O_2_ even without Fe whereas **2 a** forms only in the presence of Fe after approximately a 30‐60 minute induction period (for details, see Table S7, Table S18, and Figure S35), the formation of **2 a** likely depends not only on the presence of a radical (possibly a hydroxyl radical), but also on the slow formation of active iron species. This radical chemistry raises the question of compatibility with other organic species in a prebiotic environment. To evaluate the possibility that simple organic radical scavengers could inhibit the hydration reaction, we performed two additional control experiments using 2‐propanol and *tert‐*butanol. When the reaction was run in the presence of 2‐propanol, the formation of **2 a** was not affected (15 % yield) while the formation of **2 b** was affected (6 % yield of **2 b** compared to approx. 12 % in our standard conditions, Table S10, entry 3). However, when *tert‐*butanol was used as a hydroxyl radical scavenger, the formation of **2 a** decreased (8 % yield) while the formation of **2 b** greatly increased (24 % yield, Table S10, entry 4). These experiments show that the presence of radical scavengers, as we would expect in a prebiotic reaction network, does not necessarily inhibit the hydration reaction. However, future work aiming to use these conditions for the development of a complex chemical reaction network will have to consider and evaluate the possibility of radical scavenging mechanisms.

### Hydration of other Metabolites

**Figure 5:**
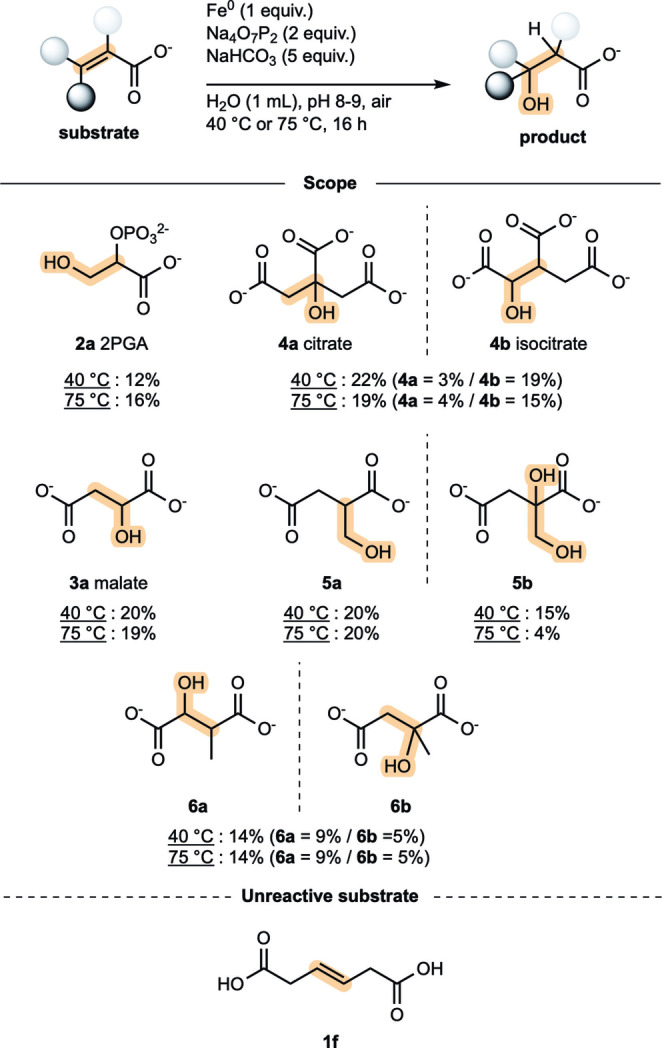
Scope of the reaction. Quantification of the product by ^1^H qNMR (for details, see Table S11–S16).

**Figure 6:**
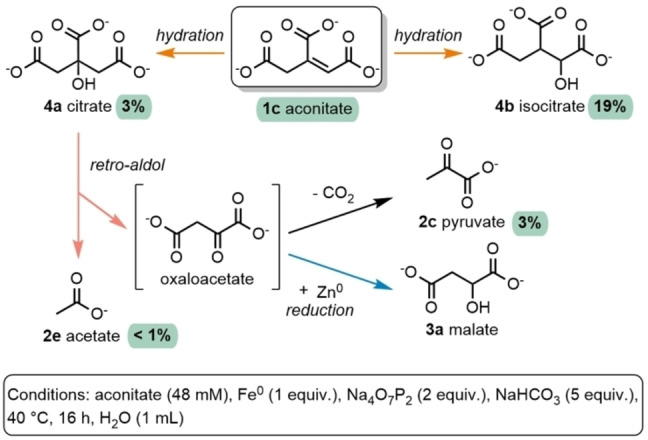
Hydration of aconitate and retro‐aldol of citrate under a common set of conditions. The products were quantified by ^1^H qNMR (for details, see Table S13). Oxaloacetate is not stable under the standard conditions but can be trapped using Zn^0^ as a reducing agent (blue arrow), in which case malate **3 a** was observed when starting from citrate **4 a**.

The hydration conditions were applied to other common metabolites found in core metabolism (Figure 5). Unlike **1 a**, most of the other hydration reactions in core metabolism do not act on a terminal alkene. Fumarate (**1 b**), a symmetric alkene, underwent hydration to malate (**3 a**) in 20 % and 19 % yields after 16 h at 40 °C or 75 °C, respectively (Table S11). Next, we studied the hydration of aconitate (**1 c**), whose alkene is not symmetric and thus presents two potential sites of hydration. By quenching a series of identical reactions at different times, we observed the formation of citrate (**4 a**) by ^1^H NMR along with a mixture of diastereoisomers of isocitrate (**4 b**), the three potential hydration products of **1 c**, again forming only after approximately a 1 h induction period (Figure S26). After a reaction time of 16 h at 40 °C, 22 % of hydrated products were obtained with **4 b** as the major product (19 %, mixture of diastereoisomers, Table S12) and **4 a** as the minor product (3 % yield). The hydrated products **3 a**, **4 a** and **4 b** could also be observed at 25 °C, but lower yields were obtained in that case (Table S11–S12). From the hydration of **1 c**, side products **2 c** (3 % yield, Table S12, entry 3) and **2 e** were also observed. These could be formed through the retro‐aldol reaction of **4 a** to **2 e** and oxaloacetate, which decarboxylates to form **2 c** (Figure 6). Indeed, when **4 a** was subjected to our standard conditions, **2 c** and **2 e** were detected, whereas this was not the case when starting from **4 b** (Table S13, see also Figure S28A–B). To support the intermediacy of oxaloacetate in this process, we set out to trap it using Zn^0^ by reducing it to malate (**3 a**) prior to decarboxylation to **2 c**. When **4 a** was subjected to standard conditions at 40 °C in the presence of 5 equiv. of Zn^0^, **3 a** was observed in 1 % yield in addition to **2 c**, and **2 e** (Table S13, see also Figure S28C). This result is notable as it shows that these conditions are compatible not only with all the hydration reactions in the rTCA cycle and gluconeogenesis but also with the retro‐aldol reaction in the rTCA cycle, a nonenzymatic reaction that had been previously observed only in aqueous microdroplets generated under high pressure.^[40]^

The regioselectivity of hydration was investigated. Itaconate (**1 d**), which features a terminal alkene, and mesaconate (**1 e**), were used as model substrates in our standard conditions (Figure 5). In the hydration of **1 d**, like that of **1 a**, we observed an exclusive oxa‐Michael hydration with formation of **5 a** in 20 % yield (for details, see Table S14). Moreover, we identified the formation of a dihydroxylation side product (**5 b**, Figure S29) in 15 % yield (after 16 h at 40 °C), which might, like **2 b**, have been formed from epoxidation followed by hydrative ring‐opening. Subjecting **1 e** to our standard conditions gave a mixture of hydration regioisomers **6 a** and **6 b** in 14 % combined yield in a 9 : 5 ratio, favoring hydration at the least substituted site (for details, see Table S15 and Figure S30). The influence of the position of the carboxylate group relative to the alkene was evaluated. Attempts to hydrate diacid **1 f** were unsuccessful, emphasizing the importance of conjugation between alkene and carboxylate (Figure 5, for details, see Table S16).

**Figure 7:**
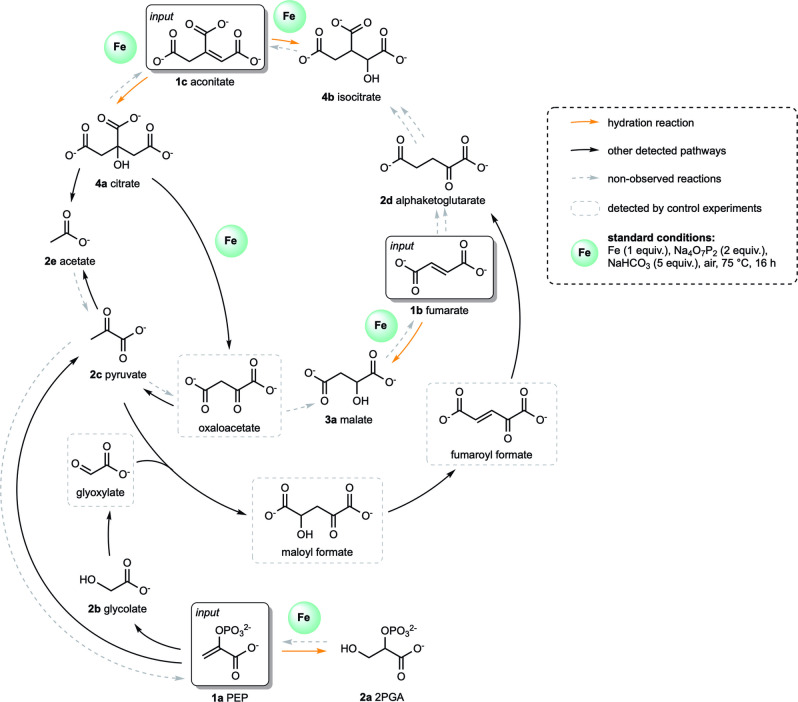
The nonenzymatic reaction network is generated from the hydration of key metabolites (i.e., PEP, aconitate, and fumarate). **1 a**; **1 b**, and **1 c** were added simultaneously in a one‐pot manner and products **2 a**–**2 e**, **3 a** and **4 a**–**4 b** were detected by ^1^H qNMR (for details, see Table S17). Products in dashed grey boxes were not detected directly by ^1^H NMR within the reaction network but observed via control experiments (for details, see Figure S18, S20 and S28 C).

Finally, we tested the ability of the various hydration reactions (and their respective reaction networks) to occur in the same environment, and their potential competition (Figure 7). When a mixture of **1 a**, **1 b**, and **1 c** at similar concentrations was subjected to our standard conditions (48 mM, 1 equiv. of **Fe^0^**), we observed the formation of **2 a**–**2 e**, **3 a** and **4 a**–**4 b** by ^1^H NMR (Figure S32). The hydrated products were obtained in 5 % yield each for **2 a**, **3 a**, and **4 b** whereas **4 a** was formed in 1 % yield for a total of 16 % yield of hydrated products (Table S17, entry 1). Moreover, when we increased the equivalents of **Fe^0^** from 1 to 3, the ratio between the different hydrated products did not change significantly. In this case, **2 a**, **3 a**, and **4 b** were formed in 8 %, 11 %, and 9 % yields, respectively, while **4 a** was formed in 2 % yield; 30 % overall yield (Table S17, entry 2). These experiments demonstrate the existence of continuous (in the sense of happening at the same time in a one‐pot manner^[65]^) reaction networks occurring in parallel and involving several different classes of transformations, including hydration (formation of **2 a**; **3 a**, **4 a**, and **4 b**), dehydration (maloyl formate to fumaroyl formate), aldol (glyoxylate and **2 c** to maloyl formate) and retro‐aldol (**4 a** to oxaloacetate and **2 e**), oxidation (**2 b** to glyoxylate) and reduction (fumaroyl formate to **2 d** and oxaloacetate to **3 a**), hydrolysis (**1 a** to **2 c**), and decarboxylation (**2 c** to **2 e**). Neither new reactions nor competitive behaviors were observed in the mixture, highlighting the absence of cross‐inhibition between the chemical reaction networks, a fundamental aspect for understanding and developing a complex chemical reaction network and “life‐like” behaviors.^[65]^

### Prebiotic Relevance of this Study

Our study shows that all hydration reactions found in the rTCA cycle and gluconeogenesis can occur under a common set of mild nonenzymatic conditions (25–75 °C in aqueous bicarbonate solution). The reaction works under oxidative conditions in the presence of a reduced iron source (either Fe^0^, Fe^2+^, or green rust) and pyrophosphate, components that can be found on early Earth.

Although the steady‐state concentration of O_2_ in the prebiotic atmosphere is thought to have been very low, several studies now show that O_2_ was continuously produced locally on the Archean Earth by mechanochemical water splitting due to turbulent water flow over minerals,[^66^, ^67^] or through radiolysis of water by ionizing radiation of ^40^K isotopes.^[68]^ Recently, Sweetman et al. demonstrated the production of “dark oxygen” under anaerobic conditions by electrochemical water splitting over the surface of metals with concentrations of 300–400 μmol/L,^[69]^ values above the solubility of oxygen from air in water (290 μmol/L at 20 °C under 1 atm of air^[70]^). In addition, when we performed the hydration reaction in a N_2_‐filled glovebag using deoxygenated water, we still observed trace amounts of hydrated products due to remaining trace amounts of oxygen (for details, see Figure S23, p. S41). Therefore, even in the presence of very low concentrations of oxygen, the hydration reaction still occurred.

The abovementioned studies about anaerobic oxygen production also report the formation of reactive oxygen species (ROS) such as H_2_O_2_ and hydroxyl radicals, prior to the formation of O_2_. Among the various ways to form ROS, for instance by photolysis of atmospheric water vapor[^71^, ^72^] or by harnessing the properties of the water‐air interface of aqueous microdroplet,[^73^, ^74^] the ones reported at mineral‐water interfaces are of particular interest. Indeed, several studies demonstrated that ROS could be formed on naturally abundant sulfide minerals^[75]^ such as pyrite,[^76^, ^77^, ^78^, ^79^] which is proposed as a mineral of prime interest for the origin of life.[^3^, ^80^, ^81^, ^82^] Interestingly, these studies showed the formation of ROS in an anaerobic environment using abundant minerals containing iron[^76^, ^77^, ^78^, ^79^] and silicates[^66^, ^67^] naturally found in hydrothermal vents.[^3^, ^83^, ^84^, ^85^] Such environments present basic pH^[85]^ and a temperature range of 25 to 125 °C,[^84^, ^86^] both compatible with the conditions reported in this study for alkene hydration.

Another key element of the reported conditions is pyrophosphate. Polyphosphates such as pyrophosphate are thought to have been produced by geochemical processes on the early Earth.[^87^, ^88^, ^89^, ^90^] Interestingly, it was reported that pyrophosphate could be formed through the oxidation of reduced phosphorous (HPO_3_^2−^ or H_2_PO_2_^−^) in the presence of H_2_O_2_.^[87]^ In this study, pyrophosphate was used as a chelating agent for iron species, also under oxidative conditions, potentially preventing the formation of iron precipitates in solution,^[50]^ and allowing the nonenzymatic hydration of core metabolites.

Lastly, the key promoter of the reaction is an iron‐based species. Iron is of particular interest because it is one of the most abundant metals on Earth,^[91]^ plays fundamental roles in biology,^[31]^ and has been shown to be among the most efficient in promoting nonenzymatic metabolic reactions.[^11^, ^15^, ^16^, ^20^, ^92^, ^93^, ^94^, ^95^, ^96^, ^97^, ^98^, ^99^, ^100^] Notably, iron can be naturally found in the form of green rust, a mixture of Fe(II)/Fe(III) hydroxy salts. In this study, green rust was found to be a suitable source of iron for the reaction. Of particular interest is that the reported conditions are closely related to those that form carbonate green rust, which is thought to have covered the surface of the primitive ocean,^[45]^ and has been proposed as an “organizing seed” for the emergence of life.[^45^, ^101^, ^102^]

## Conclusion

We report mild nonenzymatic conditions (25–75 °C in aqueous bicarbonate solution) for all hydration reactions in the rTCA cycle, gluconeogenesis, and on metabolites from other pathways. Notably, the nonenzymatic hydration of **PEP** to **2PGA** was reported for the first time. The conditions also enable a nonenzymatic version of the retro‐aldol reaction of citrate found in the rTCA cycle, which was so far only reported in aqueous microdroplets. The conditions rely on a combination of a reduced iron source (either Fe^0^, Fe^2+^, or green rust), pyrophosphate, bicarbonate, and oxygen.

Preliminary mechanistic insights indicate that the reaction depends on at least one open‐shell species and the slow formation of an active iron species, which becomes inactivated over time due to overoxidation. The hydration occurs exclusively on α,β‐unsaturated carboxylate groups, and does so with a mild preference for the formation of the C−O bond at the least substituted carbon. The hydration reactions of the rTCA cycle and gluconeogenesis were found to all occur simultaneously with dehydration, (retro)aldol, oxidation/reduction, hydrolysis, and decarboxylation in the same pot under a common set of mild nonenzymatic conditions. Further research will show how the identified conditions can be merged with more complex reaction networks such as sequences of the rTCA cycle and of gluconeogenesis.

The present results are part of a larger effort to triangulate the conditions under which chemistry might self‐organize into a complex chemical reaction network (i.e. a protometabolism).^[103]^ In contrast to previous efforts to search for conditions relevant to reactions within a single metabolic pathway, here we took a complementary approach by screening for nonenzymatic conditions for reaction classes that transcend pathways. Future efforts to investigate by reaction class, rather than pathway, should continue to narrow the search.

## Supporting Information

The authors have cited additional references within the Supporting Information.[^104^, ^105^]

## Conflict of Interests

The authors declare no conflict of interest.

1

## Supporting information

As a service to our authors and readers, this journal provides supporting information supplied by the authors. Such materials are peer reviewed and may be re‐organized for online delivery, but are not copy‐edited or typeset. Technical support issues arising from supporting information (other than missing files) should be addressed to the authors.

Supporting Information

## Data Availability

The data that support the findings of this study are available in the supplementary material of this article.
