# Supplementary material for: Nonenzymatic Hydration of Phosphoenolpyruvate: General Conditions for Hydration in Protometabolism by Searching Across Pathways
Source: Angew Chem Int Ed Engl. 2024 Dec 2;64(2):e202410698. doi: 10.1002/anie.202410698 (PMC11720399; doi:10.1002/anie.202410698)
Supplement: Supplementary file 1 — Supporting Information [file ANIE-64-e202410698-s001.pdf]

Angewandte Chemie
Eine Zeitschrift der Gesellschaft Deutscher Chemiker

Supporting Information

Nonenzymatic Hydration of Phosphoenolpyruvate: General Conditions for Hydration in Protometabolism by Searching Across Pathways

*J. Zimmermann, A. Bora Basar, J. Moran**

Supplementary Information

Nonenzymatic Hydration of Phosphoenolpyruvate: General Conditions for Hydration in Protometabolism

Joris Zimmermann,[1] Atalay Bora Basar,[1] and Joseph Moran[1,2,3]

[1] Institut de Science et d'Ingénierie Supramoléculaires (ISIS), CNRS UMR 7006, Université de Strasbourg 8 Allée Gaspard Monge, 67000 Strasbourg (France)

[2] Institut Universitaire de France (IUF) (France)

[3] Department of Chemistry and Biomolecular Sciences, University of Ottawa, Ottawa, Ontario, K1N 6N5, Canada

Contact: jmoran@uottawa.ca

Table of Content

General information..4
I. Materials..4
II. NMR and mass spectroscopy..4
A. NMR spectroscopy..4
B. HRMS spectroscopy..4
III. Analytical methods..5
A. NMR standards..5
B. Product quantification by ^{1}H qNMR..14
C. Product identification by HRMS (direct infusion)..16
Synthetic procedures and quantification..17
IV. Study and optimization of the hydration of PEP (1a) to 2PGA (2a)..17
A. Metal screening in slightly basic conditions..17
B. Additive screening using Fe^{0} and Fe^{2+} as promoters..19
C. Metal screening in the presence of pyrophosphate..21
D. Optimization – variation of the temperature and the reaction time..22
E. Optimization – Variation of the different parameters..24
F. Buffer and pH effects..25
G. Reaction Profile, Mass balance, and Reaction Network..26
H. Investigating the formation of the side products of the reaction..32
1) Stability and decomposition of the products of the reaction..32
2) Study of α-ketoglutarate formation..35
3) Study of glycolate formation..37
V. Investigating the hydration of PEP to 2PGA..39
A. Study of the promoter for the hydration reaction..39
B. Control experiments to investigate the mechanistic requirements of the hydration reaction..40
1) Experiments under an inert atmosphere..40
2) Radical trapping experiments..42
VI. Scope of the reaction..45
A. General procedure for the hydration of other biological substrates..45
B. Hydration of fumarate (1b) to malate (3a)..45
C. Hydration of cis-aconitate (1c) to citrate (4a) and isocitrate (4b + 4b')..47
1) Study of the hydration of cis-aconitate (1c) over time..47
2) Hydration of cis-aconitate (1c)..49
3) Study of the retro-aldol reaction of citrate (4a)..50
D. Hydration of itaconate (1d) to (5a)..53

E. Hydration of mesaconate (1e) to (6a) 56
F. Hydration of a non-conjugated alkene (1f)...58
G. Hydration of all core metabolites (1a, 1b, and 1c) in one-pot....................................59
VII. Further investigations of the reaction mechanism 62
A. Investigation of kinetics of the reaction..62
1) General procedure for kinetics..62
2) Kinetics with variation of Fe^0 concentration..62
a) Kinetics using 1 equiv of Fe^0...62
b) Kinetics using 0.5 equiv of Fe^0...63
c) Kinetics with addition of 5% of products with 1 equiv. of Fe^0.................................64
d) Comparison between kinetics in the presence and in the absence of 5% of product...65
B. Deuterium isotope effect 66
C. Induction period experiment...67
References...69

General information

I. Materials

Unless otherwise noted, all reagents and solvents were purchased from Sigma-Aldrich, Fluka, TCI, Acros organics, or Carbosynth, and used without further purification. D(+)-2-Phosphoglyceric acid sodium salt hydrate, glycolic acid, sodium pyruvate, fumaric acid, DL-malic acid, cis-aconitic acid, citric acid, DL-isocitric acid trisodium salt hydrate, lithium β-hydroxypyruvate hydrate, glyoxylic acid monohydrate, and mesaconic acid were purchased from Sigma-Aldrich. Trans-3-hexenedioic acid was purchased from TCI. Itaconic acid and phospho(enol)pyruvic acid monopotassium salt were purchased from Alfa Aesar. The initial purity of phospho(enol)pyruvic acid monopotassium salt was up to 99%. At the end of the study, the substrate (i.e., phosphoenolpyruvate) being hygroscopic, the purity was quantified again indicating up to 95% so that this loss of purity was considered negligible for the calculation of the yields. Water was obtained from a Milli-Q purification system (18.2 MΩcm). All reactions were carried out in 2 mL Eppendorf vials with lids. Experiments were carried out in a Thermo Scientific™ Digital Heating Shaking Drybath or a Dutcher Agitateur chauffant et refroidissant HCM100-Pro without using stir bars with a speed of 800 rpm. pH values were measured using a Mettler Toledo FiveGo F2 pH-meter equipped with a Mettler-Toledo InLab® Flex-micro pH electrode. Anhydrous $FeCl_2$ was purchased from Sigma-Aldrich and was stored in the glove box to avoid degradation.

II. NMR and mass spectroscopy

A. NMR spectroscopy

^1H and ^{31}P NMR spectra were recorded on a Bruker Avance Neo-500 (500 MHz) or Bruker UltraShield Plus Avance III spectrometer (400 MHz) at ambient temperature (23 °C) in an H_2O: D_2O mixture (9:1), if not noted otherwise, using qNMR grade dimethyl sulfone (**DMS,** Sigma-Aldrich, TraceCERT) as internal standard (CH_3 group set at 3.00 ppm) and phosphonoacetate (Sigma-Aldrich, δ = 15.7 ppm in ^{31}P NMR).

Unless otherwise stated, the following parameters were used for the different NMR experiments:

Experiment	Pulse sequence	Pulse delay (d1)	Number of scans (ns)
^1H	zg30	2 s	16
^1H water suppression	noesygppr1d	2 s	16
^{31}P (^1H coupled)	zgdc30	3 s	64
^{31}P (^1H decoupled)	zgesfpgp	2 s	16
qNMR (^1H)	zg30	30 s	8, 16, or 32 scans
qNMR (^{31}P)	zgpg30	30 s	16 or 32 or 128 scans

Integration was performed using MestReNova v14.3.1 software.

B. HRMS spectroscopy

High-resolution mass spectrometry (HRMS) analysis was performed on a Thermo Scientific Exactive Plus EMR (ESI-Orbitrap) using electrospray ionization (ESI).

III. Analytical methods

A. NMR standards

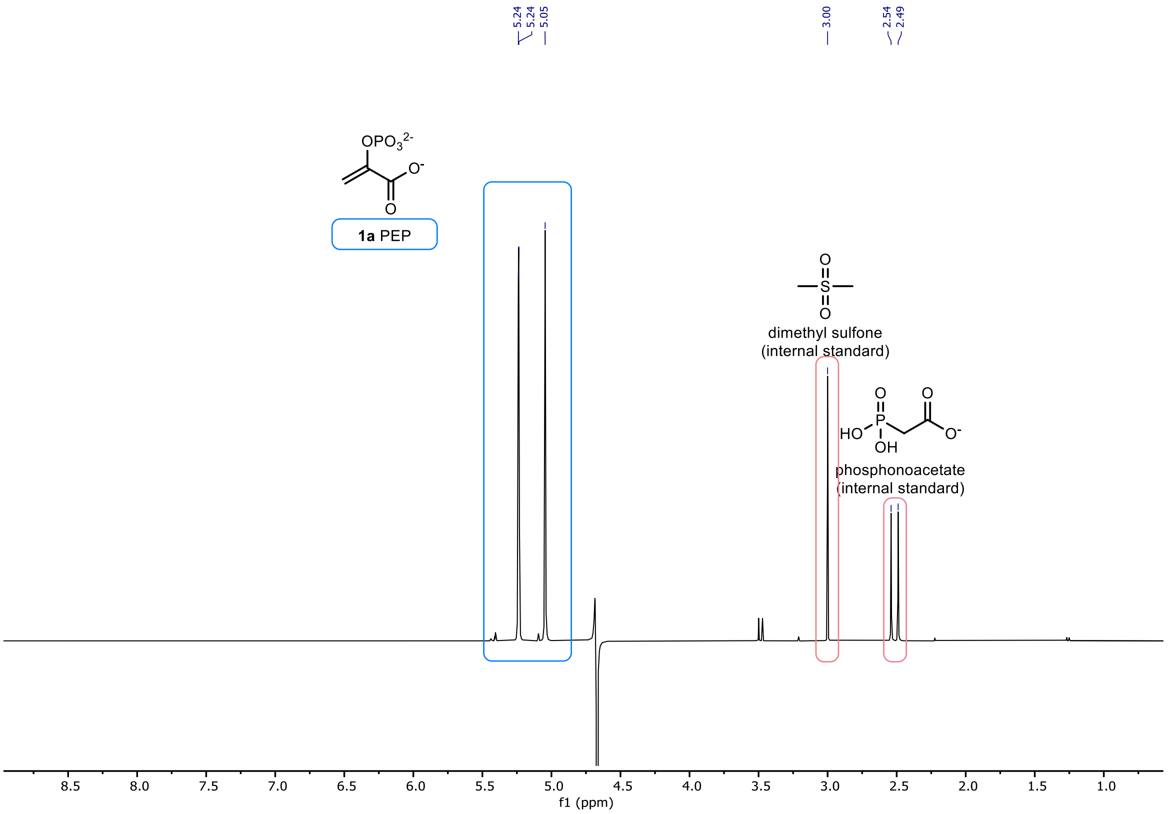

Figure S1. ^{1}H NMR (400 MHz, ns = 16, d1 = 2 s) of the starting material (phosphoenolpyruvate, **PEP**, **1a** in blue), and the two internal standards (dimethyl sulfone, **DMS** and phosphonoacetate in pink) used in this study in our standard conditions ($NaHCO_3$, 5 equiv, $Na_4P_2O_7$, 2 equiv). The mixture was prepared in an H_2O:D_2O mixture (9:1) as solvent (pH 8-9 due to the presence of $NaHCO_3$).

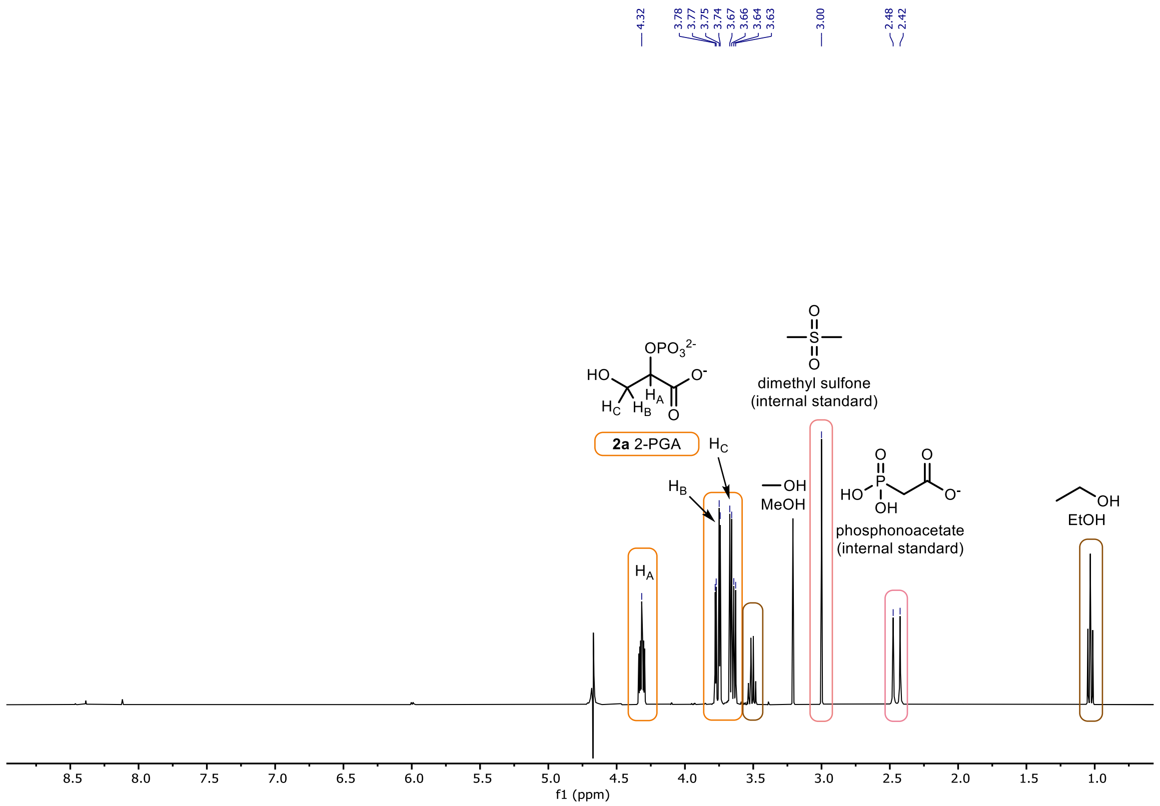

Figure S2. ^{1}H NMR (400 MHz, ns = 16, d1 = 2 s) of the product of hydration of **PEP** (2-phosphoglycerate, **2-PGA**, **2a**, in orange), and the two internal standards (dimethyl sulfone, **DMS** and phosphonoacetate in pink) used in this study in our standard conditions ($NaHCO_3$, 5 equiv, $Na_4P_2O_7$, 2 equiv). The mixture was prepared in an H_2O:D_2O mixture (9:1) as solvent (pH 8-9 due to the presence of $NaHCO_3$). The authentic sample contains ethanol and methanol impurities due to the purification method of the supplier. For quantification, the signal of $\mathbf{H_B}$ or $\mathbf{H_C}$ of the -CH_2 group of **2PGA** at 3.63-3.78 ppm was chosen for the quantification over the other proton at 4.32 ppm to minimize the loss of integration due to the ^{1}H water suppression method (see Fig. S11 in the case of **PEP**).

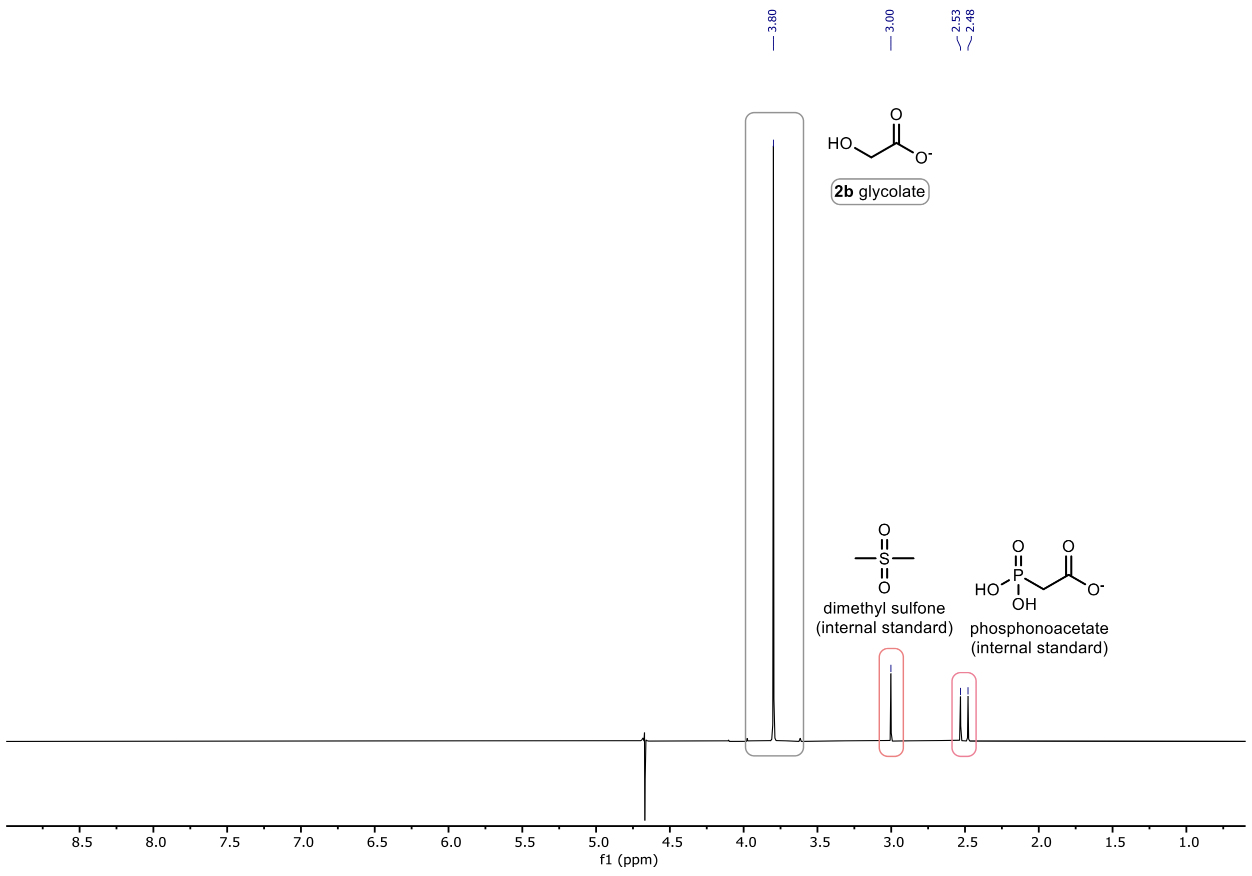

Figure S3. ^{1}H NMR (400 MHz, ns = 16, d1 = 2 s) of the product of oxidative decomposition of **PEP** (glycolate **2b** in grey) and the two internal standards (dimethyl sulfone, **DMS** and phosphonoacetate in pink) used in this study in our standard conditions ($NaHCO_3$, 5 equiv, $Na_4P_2O_7$, 2 equiv). The mixture was prepared in an H_2O:D_2O mixture (9:1) as solvent (pH 8-9 due to the presence of $NaHCO_3$).

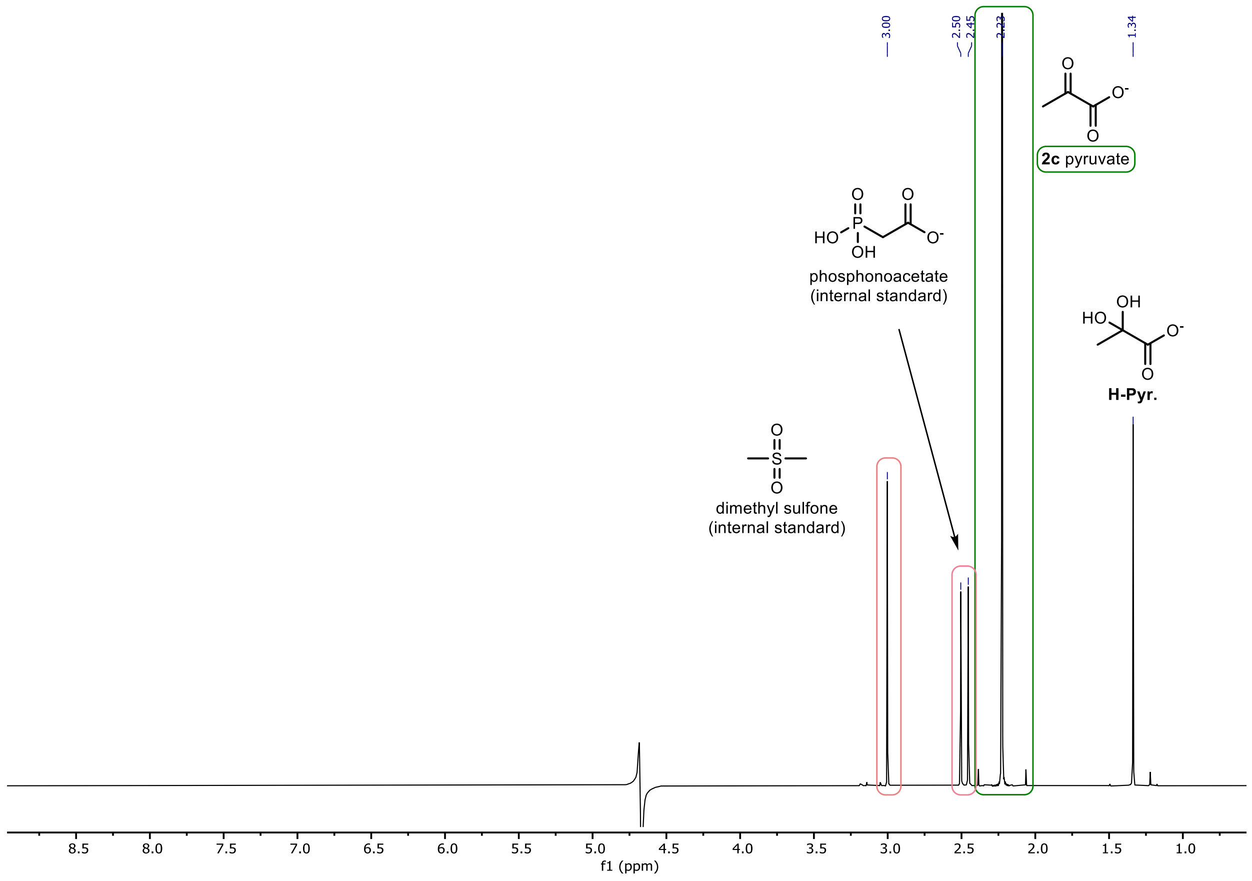

Figure S4. ^{1}H NMR (400 MHz, ns = 16, d1 = 2 s) of the product of hydrolysis of **PEP** (pyruvate **2c** in green and its hydrated form pyruvate hydrate **H-Pyr**), and the two internal standards (dimethyl sulfone, **DMS** and phosphonoacetate in pink) used in this study in our standard conditions ($NaHCO_3$, 5 equiv, $Na_4P_2O_7$, 2 equiv). The mixture was prepared in an H_2O:D_2O mixture (9:1) as solvent (pH 8-9 due to the presence of $NaHCO_3$).

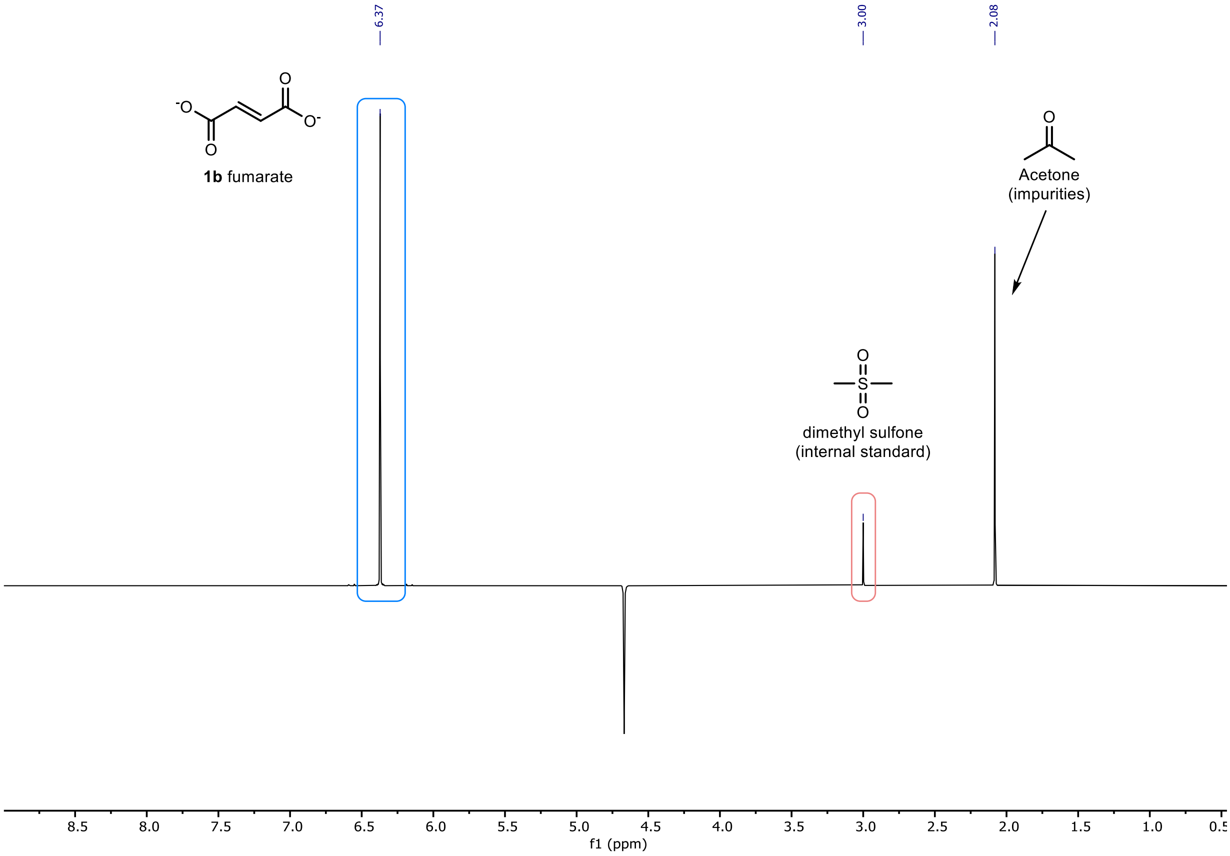

Figure S5. ^{1}H NMR (400 MHz, ns = 16, d1 = 2 s) of the starting material fumarate **1b**, and the internal standard dimethyl sulfone (**DMS** in pink) used in this study in our standard conditions ($NaHCO_3$, 5 equiv, $Na_4P_2O_7$, 2 equiv). The mixture was prepared in an H_2O:D_2O mixture (9:1) as solvent (pH 8-9 due to the presence of $NaHCO_3$).

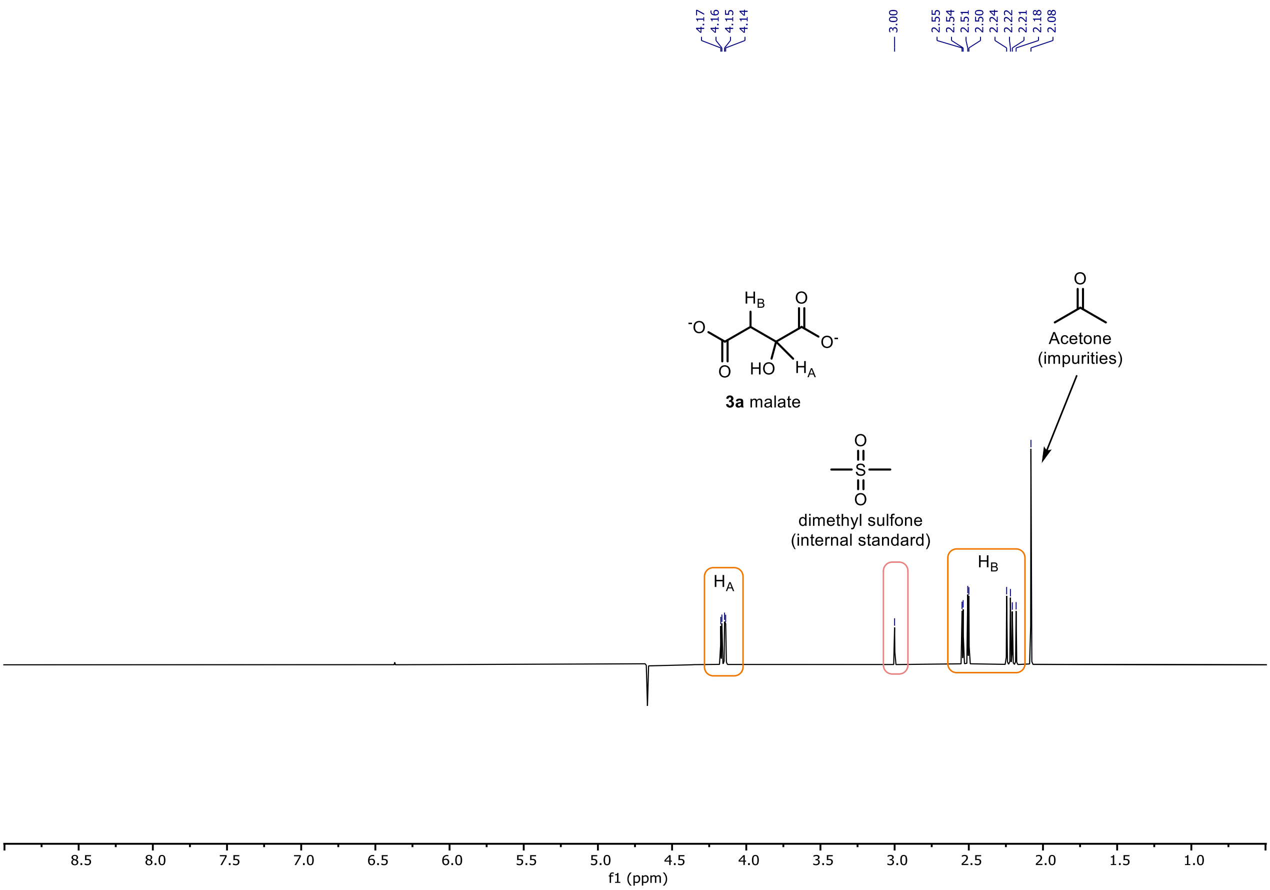

Figure S6. ^{1}H NMR (400 MHz, ns = 16, d1 = 2 s) of the product of hydration of fumarate **1b** (malate, **3b** in orange), and the internal standard dimethyl sulfone (**DMS** in pink) used in this study in our standard conditions ($NaHCO_3$, 5 equiv, $Na_4P_2O_7$, 2 equiv). The mixture was prepared in an H_2O:D_2O mixture (9:1) as solvent (pH 8-9 due to the presence of $NaHCO_3$).

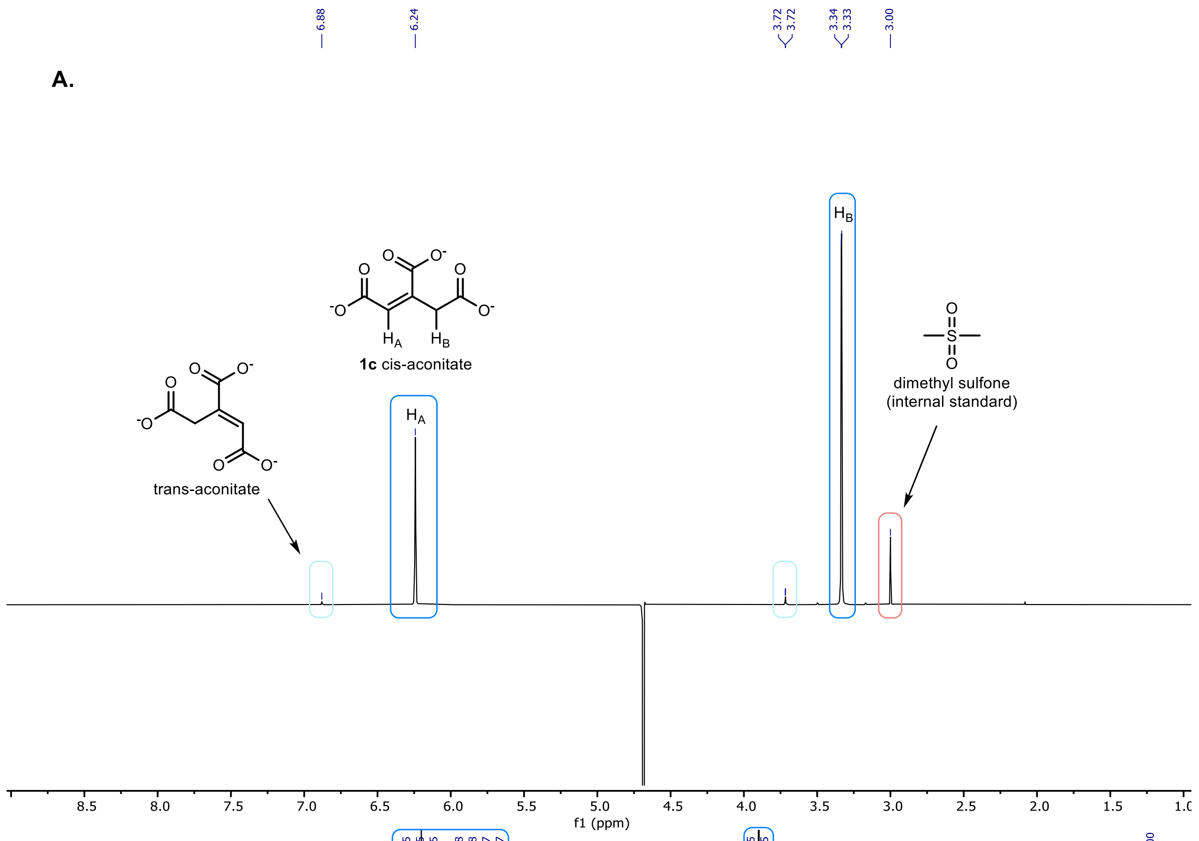
A.
trans-aconitate
1c cis-aconitate
dimethyl sulfone
(internal standard)
f1 (ppm)

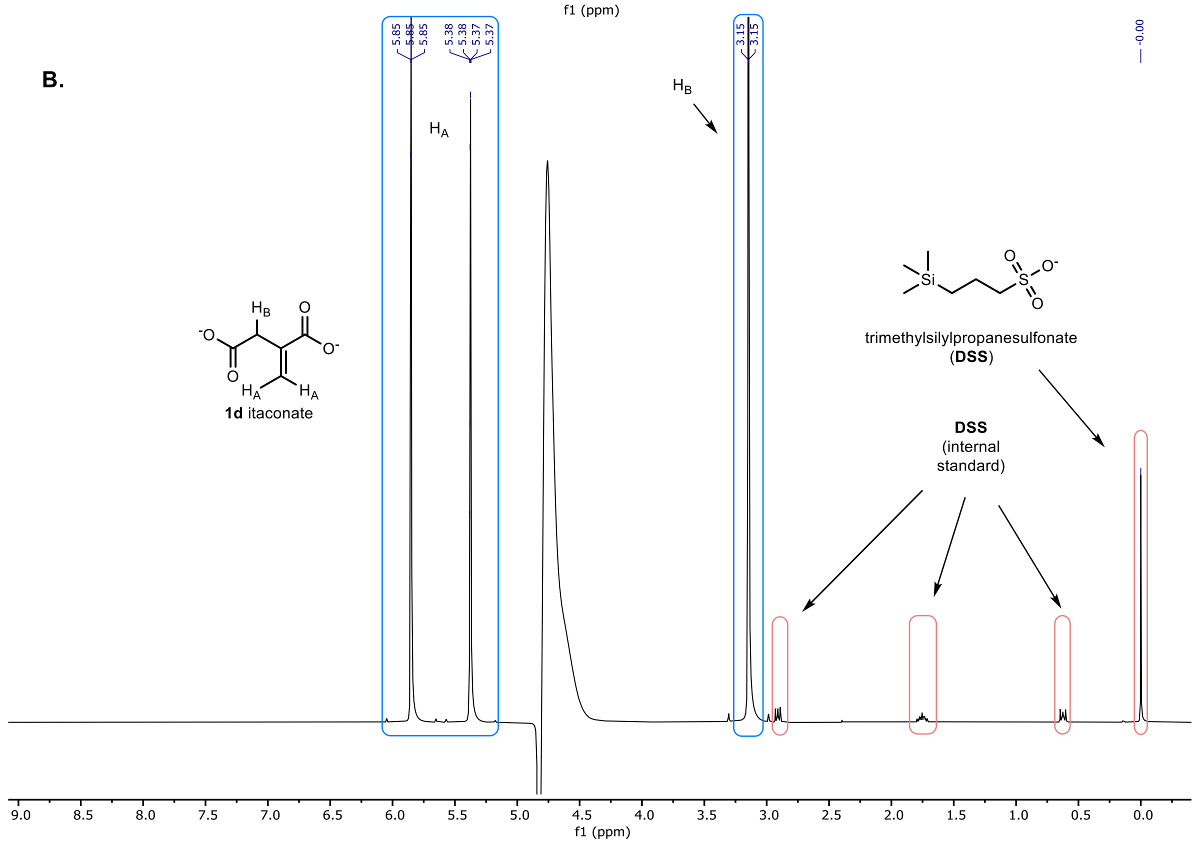
B.
1d itaconate
trimethylsilylpropanesulfonate
(DSS)
DSS
(internal
standard)
f1 (ppm)

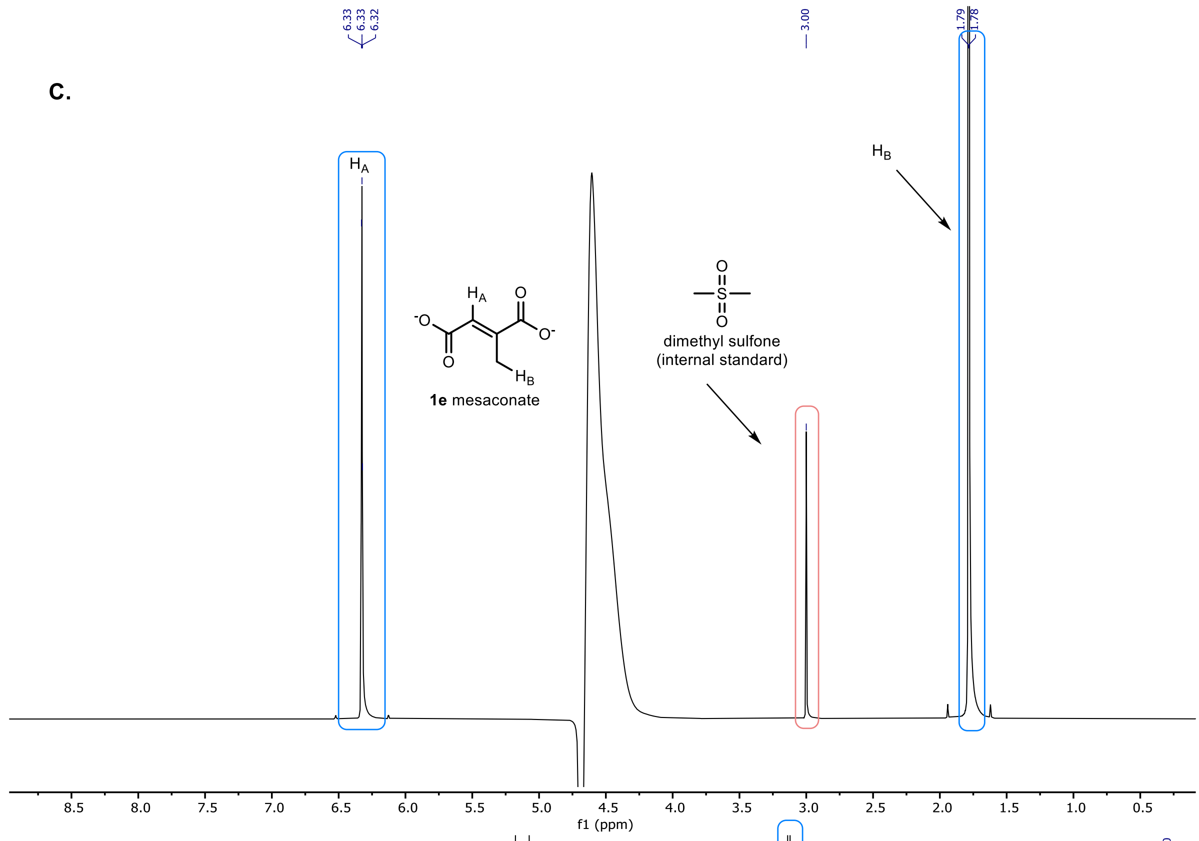

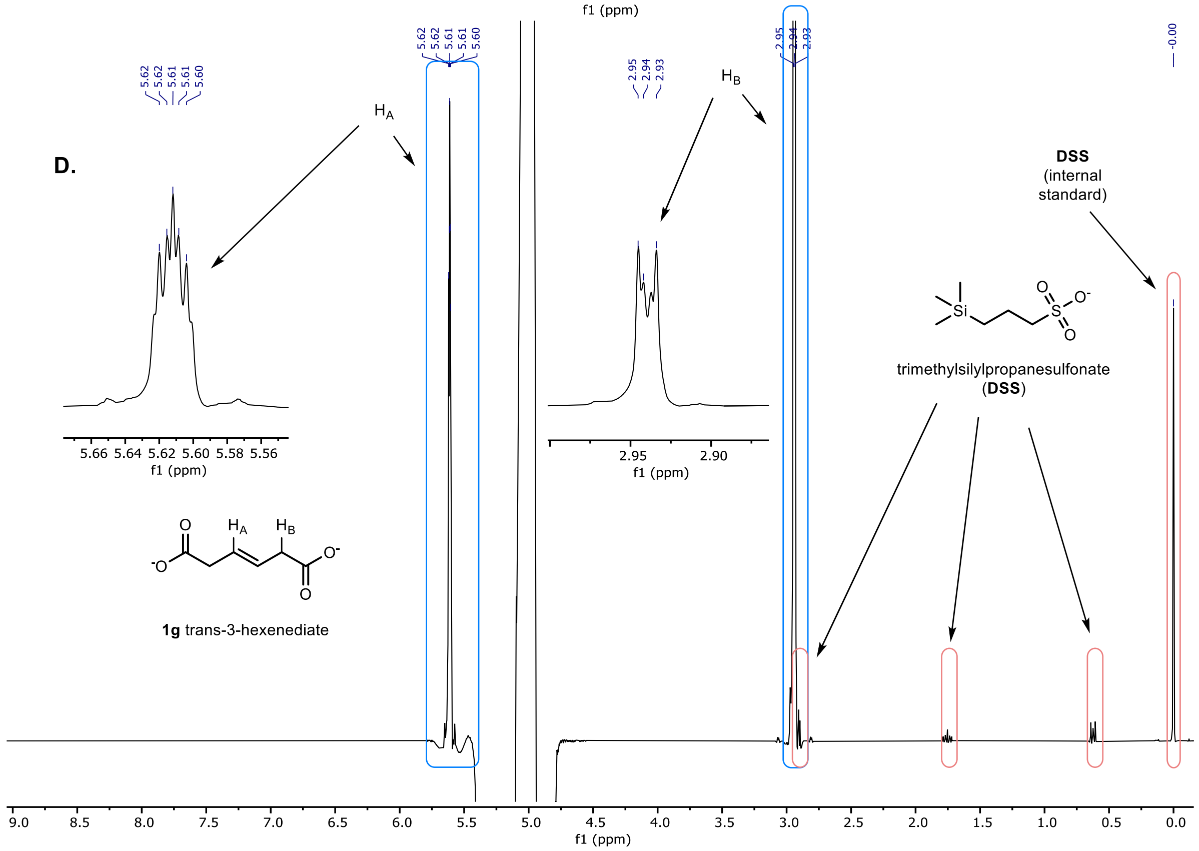

Figure S7. ^{1}H NMR spectra (400 MHz, ns = 16, d1 = 2 s) of the starting materials (in blue) and the internal standard (in pink) dimethyl sulfone (**DMS**) or trimethylsilylpropanesulfonate (**DSS**) used in this study in our standard conditions ($NaHCO_3$, 5 equiv, $Na_4P_2O_7$, 2 equiv). The mixtures were prepared in an H_2O:D_2O mixture (9:1) as solvent (pH 8-9 due to the presence of $NaHCO_3$). (A) cis-aconitate **1c** in equilibrium with trans-aconitate (in cyan); (B) itaconate **1d**; (C) mesaconate **1e**; (D) trans-3-hexenediate **1f**.

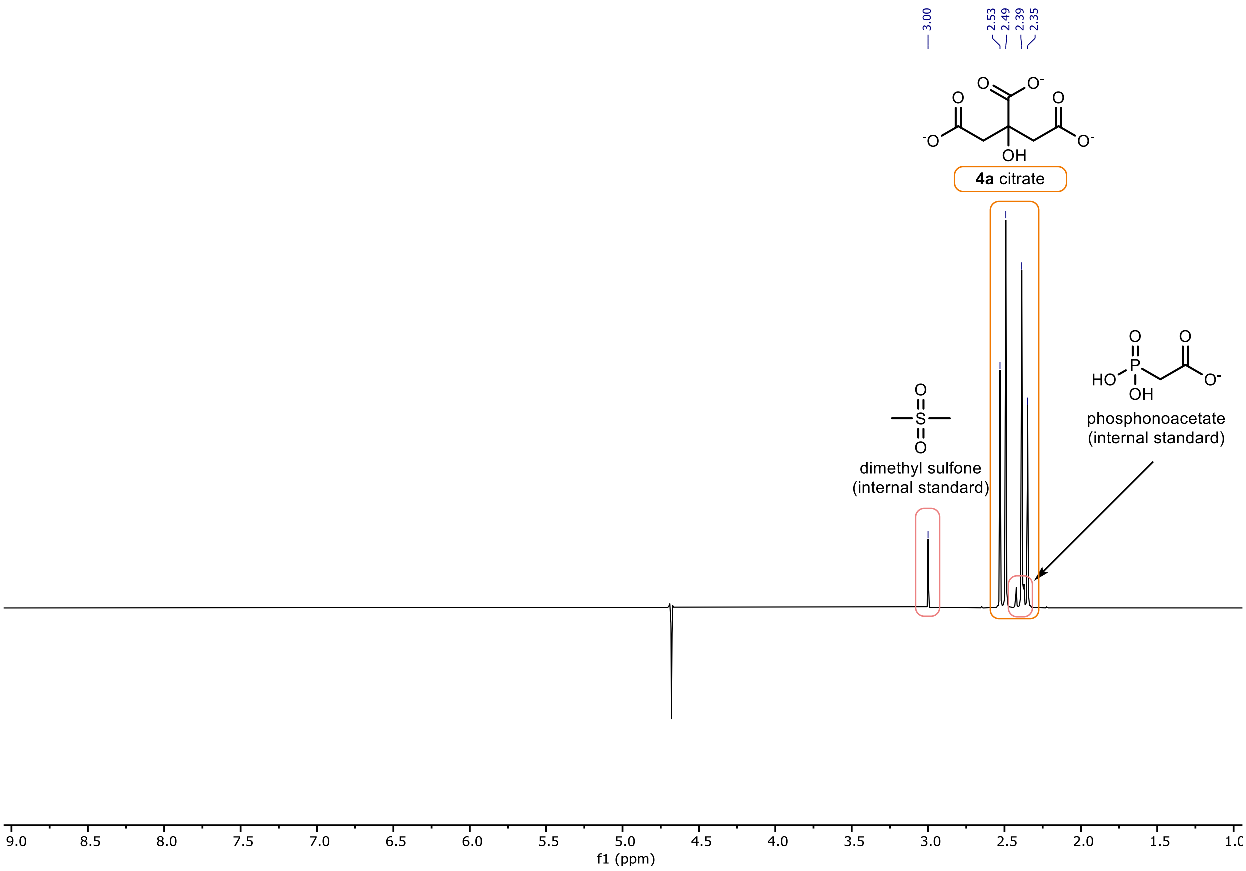

Figure S8. ^{1}H NMR (400 MHz, ns = 16, d1 = 2 s) of one of the products of hydration of cis-aconitate **1c** (citrate **4a** in orange), and the two internal standards (dimethyl sulfone, **DMS,** and phosphonoacetate in pink) used in this study in our standard conditions ($NaHCO_3$, 5 equiv, $Na_4P_2O_7$, 2 equiv). The mixture was prepared in an H_2O:D_2O mixture (9:1) as solvent (pH 8-9 due to the presence of $NaHCO_3$).

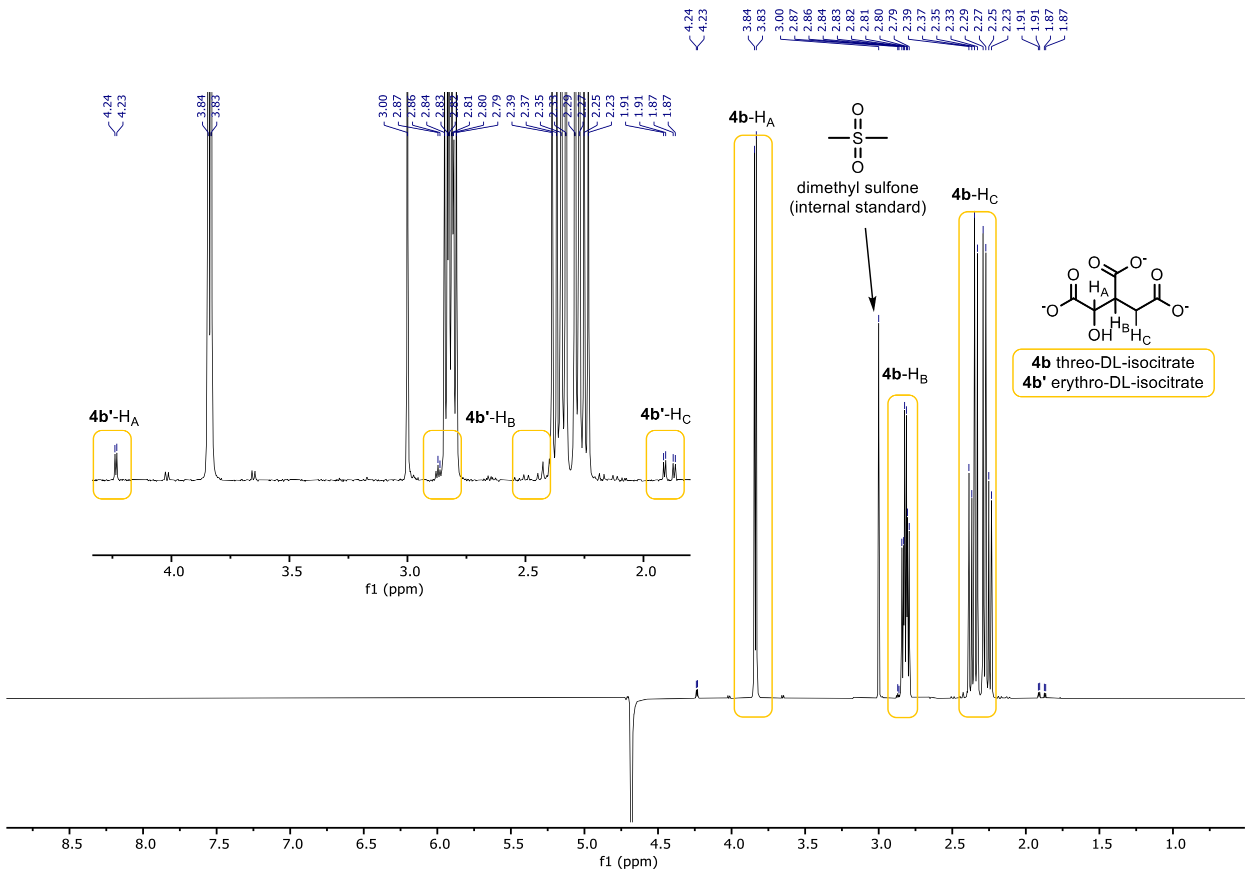

Figure S9. ^{1}H NMR (400 MHz, ns = 16, d1 = 2 s) of the other product of hydration of cis-aconitate **1c** (*threo*-DL-isocitrate **4b** along with traces amount of its diastereoisomers *erythro*-DL-isocitrate,[1] **4b'** in orange), and the internal standard dimethyl sulfone (**DMS**) used in this study in our standard conditions ($NaHCO_3$, 5 equiv, $Na_4P_2O_7$, 2 equiv). The mixture was prepared in an H_2O:D_2O mixture (9:1) as solvent (pH 8-9 due to the presence of $NaHCO_3$).

B. Product quantification by ^{1}H qNMR

General procedure (GP) for NMR sample preparation in the presence of metals: The removal of paramagnetic metal ions was performed using a procedure adapted from ref. S2.[2] After the reaction, the sample was centrifuged and 700 µL of the supernatant was transferred to a 2 mL Eppendorf vial. To this was added 150 µL (if not noted otherwise) of a thiolate/phosphate solution (1 g NaSH in 10 mL saturated aqueous Na_3PO_4), resulting in the solution turning deep black (Fig. S10C). After the addition of the thiolate/phosphate solution, the pH of the solution reached 13-14. The sample was then placed in the fridge (3 °C) where a black precipitate was formed over a few hours. The sample was then centrifuged to separate the supernatant from the black precipitate (Fig. S10D), and 500 µL of the supernatant was transferred to an NMR tube, and, if not noted otherwise, 50 µL of a 23.6 mM stock solution of **DMS** (dimethyl sulfone in D_2O) and 50 µL of a 77.8 mM stock solution of phosphonoacetate as internal standards were added.

Product quantification by ^{1}H qNMR: the products were quantified by quantitative ^{1}H NMR (^{1}H qNMR), using 500 µL of the prepared sample (see the above procedure **GP**) and, if not noted otherwise, 50 µL of a 23.6 mM **DMS** stock solution in D_2O, and 50 µL of a 77.8 mM phosphonoacetate stock solution in D_2O (total volume of NMR sample: 600 µL). The parameters reported in the **General Information** part (NMR Spectroscopy) were d1 = 30 s and ns = 8. Yields were calculated by comparing the ^{1}H NMR integrals against the **DMS** internal standard. For the quantification of **PEP**, the proton of **PEP** at 5.21 ppm was chosen for quantification to minimize the loss of integration due to the ^{1}H water suppression method (Fig. S11).

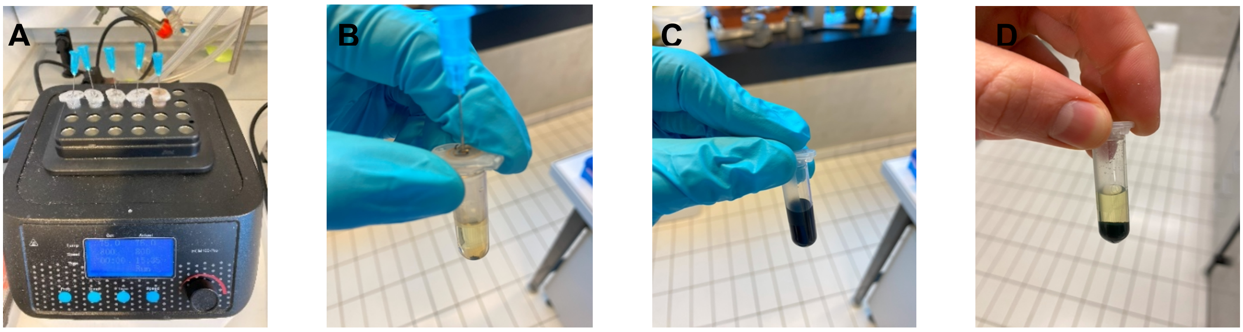

Figure S10. (A) A typical example of the setup showing the sample with a needle introduced into the top of the lid of the Eppendorf vial to perform the reaction under air without evaporation of the reaction mixture under heating. (B) A typical example of the reaction mixture after a reaction time of 16 h at 75 °C. (C) A typical example of the reaction mixture after the addition of the thiolate/phosphate solution to precipitate metals. (D) A typical example of the reaction mixture after the sample was placed in the fridge (3 °C) for a few hours and centrifugated to separate the supernatant from the black precipitate.

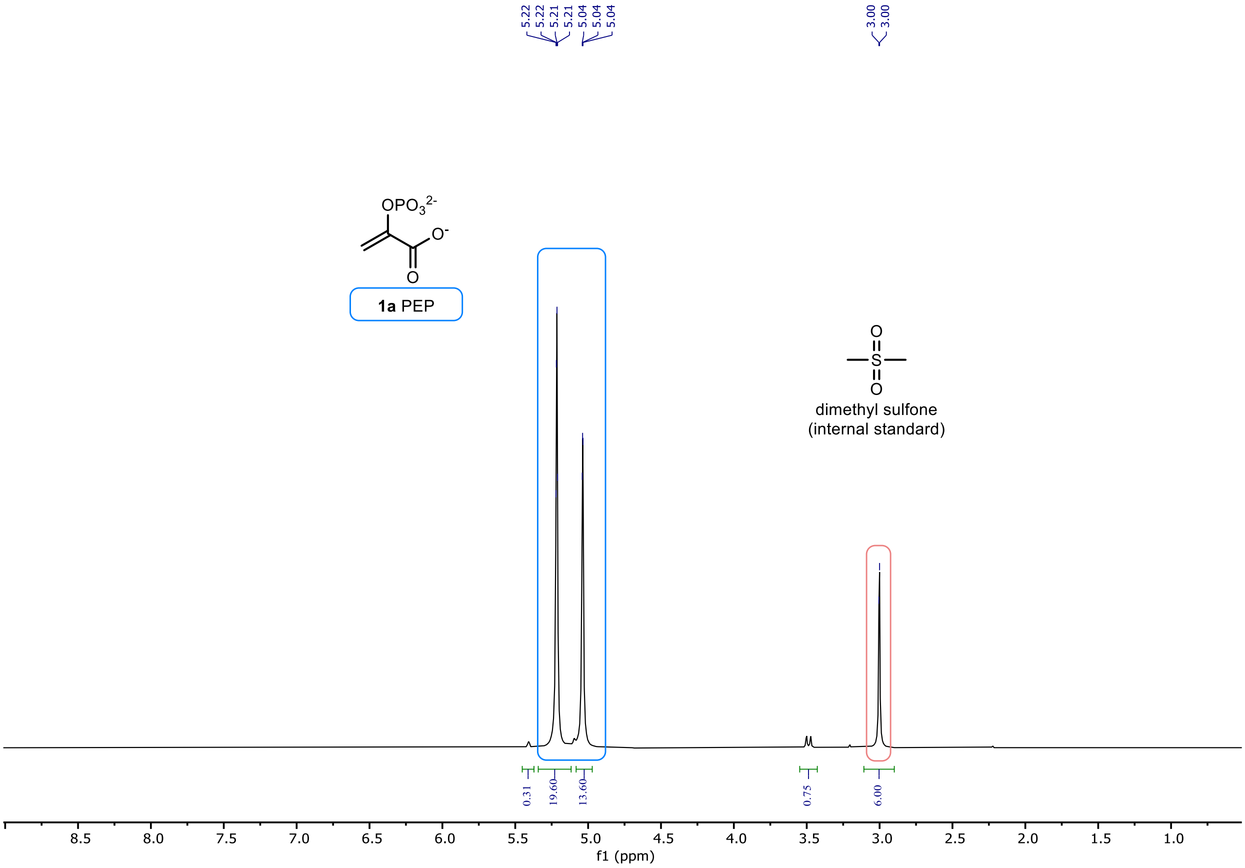

Figure S11. Quantification of **PEP** by ^{1}H qNMR (400 MHz, ns = 8, d1 = 30 s). The proton of **PEP** at 5.21 ppm was chosen for the quantification over the second one at 5.04 ppm to minimize the loss of integration due to the ^{1}H water suppression method.

C. Product identification by HRMS (direct infusion)

LC Method:

Flow Gradient

Time	Flow [mL/min]	%B	%C	%D	Curve
Equilibration					
0	Run				
0	0.4	0	100	0	5
1	0.4	0	100	0	5
3	0.4	0	60	40	5
3.2	0.4	0	100	0	5
3.5	0.4	0	100	0	5
3.501	0.4	0	100	0	5
3.501	Stop Run				

- (C): MeOH
- (D): MeOH + 0.05% formic acid

Mass Spectrometry Mode:

Full MS:

- General
  - Run time: 0 to 3.5 min
  - Polarity: Positive (or Negative)
- Full MS
  - Resolution: 140000
  - QGC target: 3e6
  - Maximum IT: 200 ms
  - Scan range: 50 to 750 m/z

Synthetic procedures and quantification

IV. Study and optimization of the hydration of PEP (1a) to 2PGA (2a)

A. Metal screening in slightly basic conditions

An aqueous stock solution of **PEP** (48 mM) and $NaHCO_3$ (5 equiv) was freshly prepared for 16 reactions as follows: **PEP** (48 mM, 160 mg) and $NaHCO_3$ (5 equiv, 323 mg) were dissolved in 16 mL of MQ H_2O. After the addition of H_2O, gas bubbles were observed, and the mixture was vortexed. Then, Eppendorf vials were charged with the metal catalyst (0.5 equiv, Table S1), and 1 mL of the freshly prepared stock solution was added to each reaction. To perform the reaction under air without evaporation of the solution under heating, a needle was introduced into the lid of the Eppendorf vial (see Fig. S10A). Samples were placed on a pre-tempered thermoshaker, and incubated for a reaction time of 24 h at 60 °C with a speed of 800 rpm. After the indicated time, the **GP** for NMR sample preparation in the presence of metal catalysts was applied. All NMR samples were analyzed by a standard water suppression pulse sequence (noesygppr1d, 400 MHz, ns = 16).

Table S1. Metal screening (n.d. = not detected).

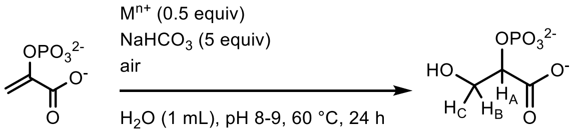

1a PEP (48 mM) → **2a** 2-PGA

Entry	Metal	Integration	Yield of 2a (%)
**1**	$FeCl_2$	Traces	Traces
**2**	$FeCl_3$	n.d.	n.d.
**3**	Fe(0)	Traces	Traces
**4**	$NiCl_2.6H_2O$	n.d.	n.d.
**5**	Ni(0)	n.d.	n.d.
**6**	$CoCl_2$	n.d.	n.d.
**7**	Co(0)	n.d.	n.d.
**8**	$MnCl_2$	n.d.	n.d.
**9**	Mn(0)	n.d.	n.d.
**10**	$ZnSO_4$	n.d.	n.d.
**11**	Zn(0)	n.d.	n.d.
**12**	$AlCl_3.6H_2O$	n.d.	n.d.
**13**	$MgCl_2$	n.d.	n.d.
**14**	$CuCl_2$	n.d.	n.d.
**15**	$CrCl_3$	n.d.	n.d.
**16**	/	n.d.	n.d.

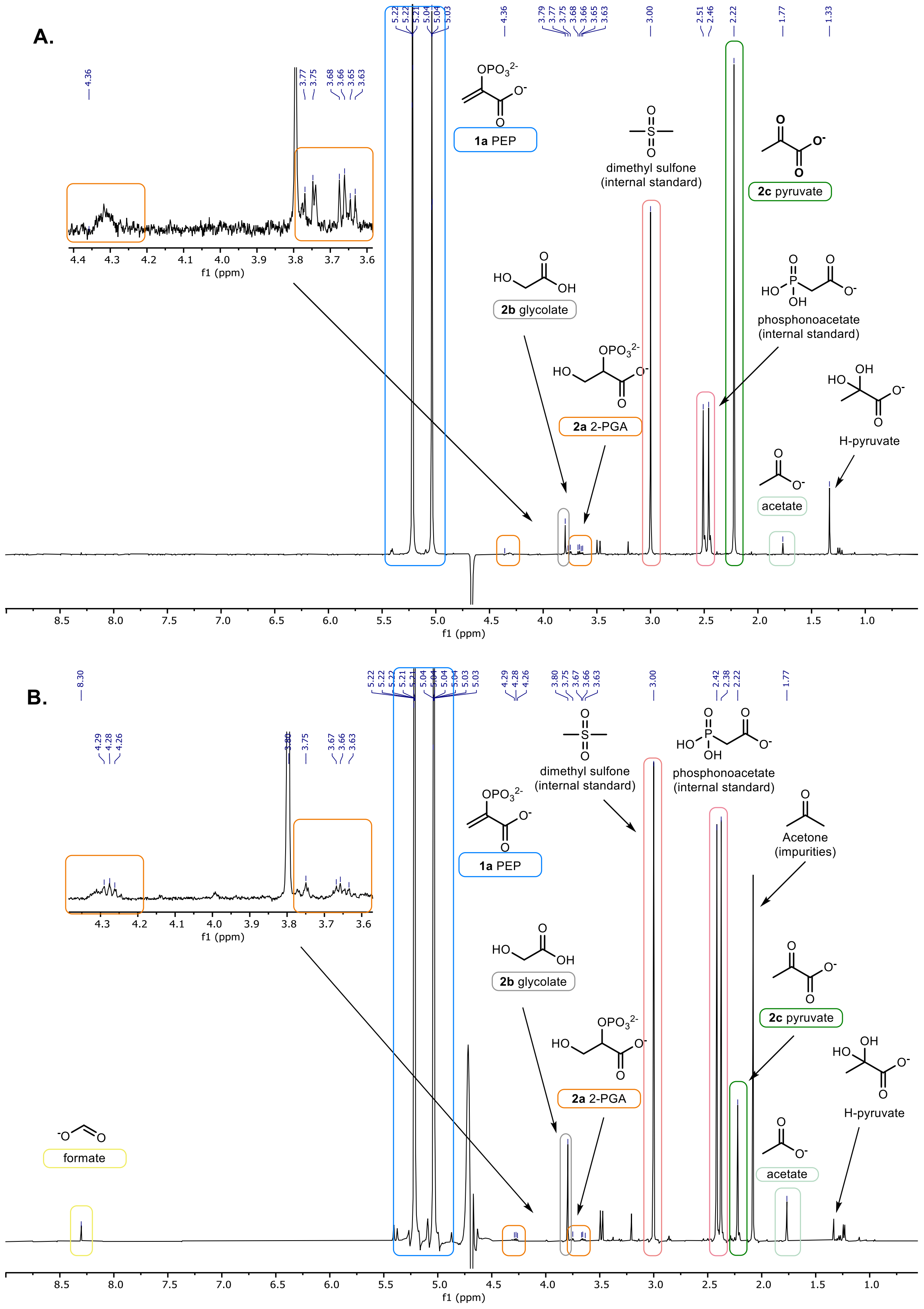

Figure S12. Typical ^{1}H NMR spectra (400 MHz, ns = 16, d1 = 2 s) for the experiments where **2-PGA** (**2a**) was observed with identification of the reaction mixture (pH 13-14 due to the addition of the thiolate/phosphate solution to precipitate metals). (A) Table S1-Entry 1. (B) Table S1-Entry 3.

B. Additive screening using Fe^0 and Fe^{2+} as promoters

An aqueous stock solution of **PEP** (48 mM) and $NaHCO_3$ (5 equiv) was freshly prepared for 20 reactions as follows: **PEP** (48 mM, 200 mg) and $NaHCO_3$ (5 equiv, 404 mg) were dissolved in 20 mL of MQ H_2O. After the addition of H_2O, gas bubbles were observed, and the mixture was vortexed. Then, Eppendorf vials were charged with the metal catalyst (1 equiv) and the additive (2 equiv, see Table S2), and, 1 mL of the freshly prepared stock solution was added in each reaction. To perform the reaction under air without evaporation of the solution under heating, a needle was introduced into the lid of the Eppendorf vial (Fig. S10A). Samples were placed on a pre-tempered thermoshaker, and incubated for a reaction time of 16 h at 75 °C with a speed of 800 rpm. After the indicated time, the **GP** for NMR sample preparation in the presence of metal catalysts was applied. For reactions containing $FeCl_2$ as a catalyst (entries 11-20), 200 µL, instead of 150 µL, of the thiolate/phosphate solution was used to precipitate the metals. All NMR samples were analyzed by a standard water suppression sequence (noesygppr1d, 400 MHz, ns = 16), and samples containing **2-PGA** (see table below) were subsequently analyzed by ^1H qNMR (d1 = 30 s, 8 scans).

Table S2. Additive screening (n.d. = not detected / n.c. = observed but not calculated).

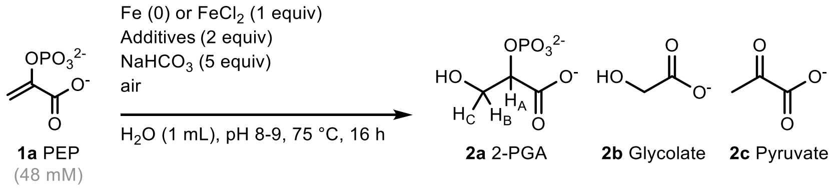

Entry	Catalyst	Additive	Int. **1a** (1 H, 5.21 ppm[b])	Yield **1a** (%)	Int. **2a** (1 H, H_B, 3.77-3.74 ppm)	Yield **2a** (%)	Int. **2b** (2 H, 3.8 ppm)	Yield **2b** (%)	Int. **2c** (3 H, 2.22 ppm)	Yield **2c** (%)	**PEP** (mM)	**DMS** (mM)[a]
1[c]		LiO_4Cl	n.c.	n.c.	traces	traces	n.c.	n.c.	n.c.	n.c.	47.8	1.965
2[c]		LiCl	n.c.	n.c.	n.d.	n.d.	n.c.	n.c.	n.c.	n.c.	47.8	1.965
3[c]		$Na_2HPO_4^{2-}$	n.c.	n.c.	n.d.	n.d.	n.d.	n.d.	n.c.	n.c.	47.8	1.964
4[d]		$Na_4O_7P_2^{4-}$	8.5	50.9	2.6	15.6	4.1	12.3	1.2	2.4	47.8	1.964
5[d]		$Na_5O_9P_3^{5-}$	9.3	55.7	2.2	13.2	3.8	11.4	1.9	3.8	47.8	1.964
6[c]	Fe (0)	1-methyl-imidazole	n.c.	n.c.	traces	traces	n.c.	n.c.	n.c.	n.c.	47.8	1.965
7[c]		KOAc	n.c.	n.c.	traces	traces	n.c.	n.c.	n.c.	n.c.	47.8	1.963
8[e]		Cysteine	n.c.	n.c.	n.d.	n.d.	n.d.	n.d.	n.c.	n.c.	47.8	1.963
9[c]		DMSO	n.c.	n.c.	n.d.	n.d.	n.c.	n.c.	n.c.	n.c.	47.8	1.965
10[c]		/	n.c.	n.c.	traces	traces	n.c.	n.c.	n.d.	n.d.	47.8	1.964
11[c]		LiO_4Cl	4.3	27.0	0.1	0.6	0.8	2.5	17.9	37.6	48.0	1.961
12[c]		LiCl	n.c.	n.c.	traces	traces	n.c.	n.c.	n.c.	n.c.	48.0	1.961
13[e]		$Na_2HPO_4^{2-}$	9.8	61.8	0.1	0.6	3.7	11.7	6.2	13.0	48.0	1.961
14[d]		$Na_4O_7P_2^{4-}$	10.0	63.1	1.2	7.6	2.3	7.3	2.2	4.6	48.0	1.961
15[d]		$Na_5O_9P_3^{5-}$	9.2	58.0	1.1	6.9	1.6	5.1	6.6	13.9	48.0	1.961
16[c]	[a]$FeCl_2$ anh.	1-methyl-imidazole.	n.c.	n.c.	traces	traces	n.c.	n.c.	n.c.	n.c.	48.0	1.961
17[c]		KOAc	3.7	23.3	0.1	0.6	0.6	1.9	19.6	41.2	48.0	1.961
18[e]		Cysteine	6.1	38.5	0.3	1.9	1.1	3.5	7.5	15.8	48.0	1.961
19[c]		DMSO	n.c.	n.c.	traces	traces	n.c.	n.c.	n.c.	n.c.	48.0	1.961
20[c]		/	5.6	35.3.	0.2	1.3	0.8	2.5	20.2	42.5	48.0	1.961

[a] The yields were determined relative to dimethyl sulfone used as an internal standard (integral set to 6 H, concentration inside the NMR tube reported in the last column).

[b] The proton of **PEP** at 5.21 ppm was chosen for quantification to minimize the loss of integration due to the ^1H water suppression method (for details, see Fig. S11).

[c] Most of the iron catalyst was precipitated in our conditions (some brown/rusty precipitates were observed at the end of the reaction and, only a low quantity of black precipitate was observed after the addition of the thiolate/phosphate solution to precipitate the iron metals).

[d] No precipitates were observed at the end of the reaction; the solution was yellowish (see Fig. S10B). Sometimes trace amounts of white precipitates could be observed at the end of the reaction before the addition of the thiolate/phosphate solution.

[e] A black precipitate was observed in the case of mixing Fe^0 or $FeCl_2$ with cysteine and a white/grey precipitate was observed in the case of mixing $FeCl_2$ with $Na_2HPO_4^{2-}$.

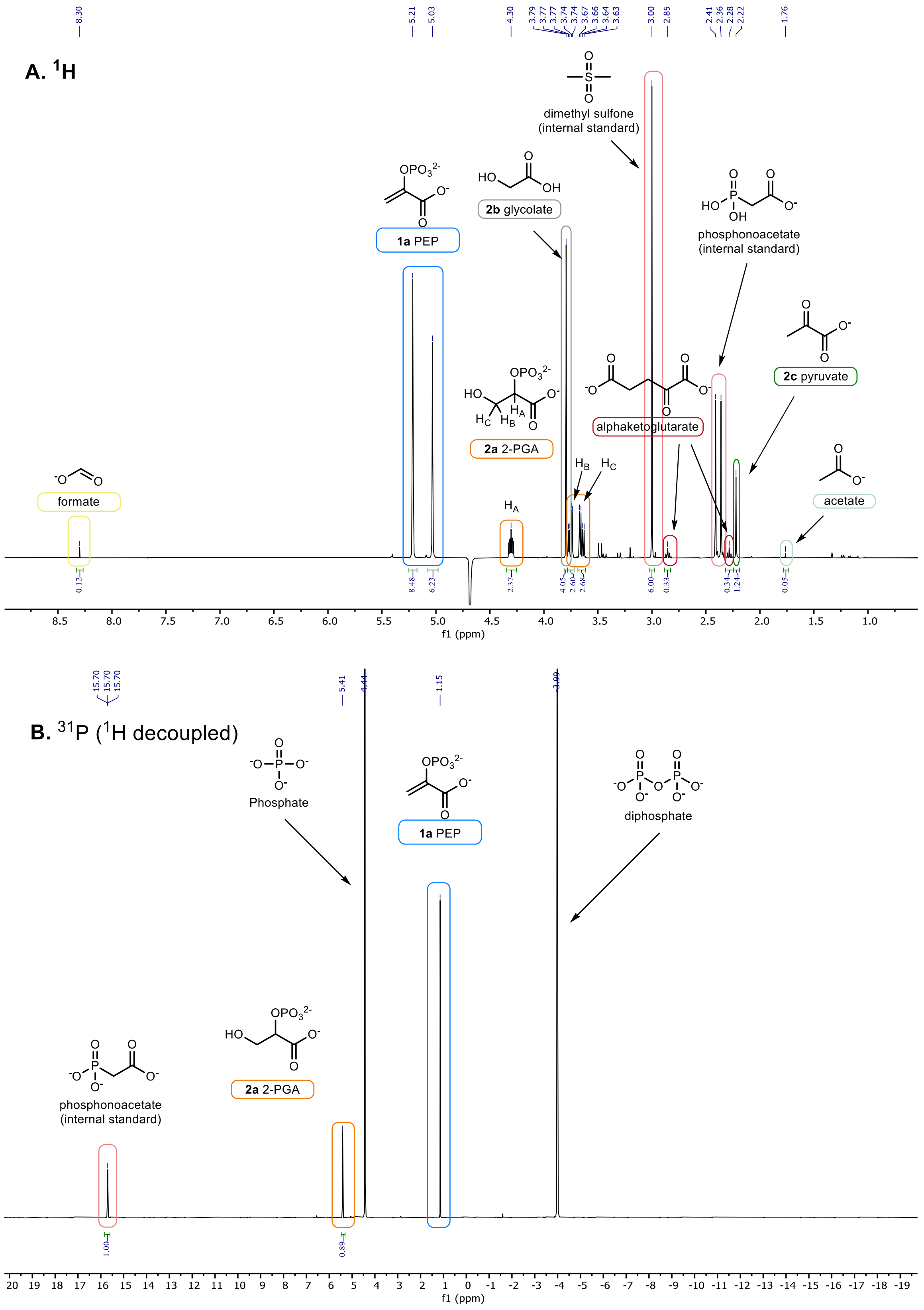

Figure S13. Typical ^{1}H and ^{31}P NMR spectra for the experiments where **2-PGA** was observed with identification of the reaction mixture (pH 13-14 due to the addition of the thiolate/phosphate solution to precipitate metals). (A) ^{1}H NMR of the Table S2-Entry 4. (B) ^{31}P NMR of the Table S2-Entry 4.

C. Metal screening in the presence of pyrophosphate

An aqueous stock solution of **PEP** (48 mM), $Na_4O_7P_2$ (2 equiv) and $NaHCO_3$ (5 equiv) was freshly prepared for 15 reactions as follows: **PEP** (48 mM, 150 mg), $Na_4O_7P_2$ (2 equiv, 382.5 mg) and $NaHCO_3$ (5 equiv, 303 mg) were dissolved in 15 mL of MQ H_2O. After the addition of H_2O, gas bubbles were observed, and the mixture was vortexed until the dissolution of the remaining $Na_4O_7P_2$ powder. Then, Eppendorf vials were charged with the metal catalyst (1 equiv), and 1 mL of the freshly prepared stock solution was added to each reaction. To perform the reaction under air without evaporation of the solution under heating, a needle was introduced into the lid of the Eppendorf vial (Fig. S10A). Samples were placed on a pre-tempered thermoshaker, and incubated for a reaction time of 24 h at 60 °C with a speed of 800 rpm. After the indicated time, the **GP** for NMR sample preparation in the presence of metal catalysts was applied. All NMR samples were analyzed by a standard water suppression sequence (noesygppr1d, 400 MHz, ns = 16), and samples containing **2-PGA** (see table below) were subsequently analyzed by ^1H qNMR (d1 = 30 s, 8 scans).

Table S3. Metal screening in the presence of $Na_4O_7P_2$ (n.d. = not detected / n.c. = observed but not calculated).

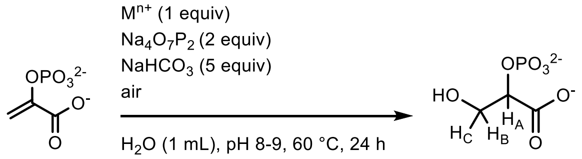

1a PEP
(48 mM)

2a 2-PGA

Entry	Metal	Int. **PEP** (1 H, 5.21 ppm)[b]	Yield **PEP** (%)	Int. **2-PGA** (1 H, H_B, 3.77-3.74 ppm)	Yield **2-PGA** (%)	**PEP** (mM)	**DMS** (mM)[a]
**1**	$FeCl_2$	9.5	52.3	0.6	3.3	47.8	1.804
**2**	$FeCl_3$	n.c.	n.c.	n.d.	n.d.	47.8	1.804
**3**	Fe(0)	7.4	40.7	2.3	12.7	47.8	1.804
**4**	$NiCl_2.6H_2O$	n.c.	n.c.	n.d.	n.d.	47.8	1.804
**5**	Ni(0)	n.c.	n.c.	n.d.	n.d.	47.8	1.804
**6**	$CoCl_2$	n.c.	n.c.	n.d.	n.d.	47.8	1.804
**7**	Co(0)	n.c.	n.c.	n.d.	n.d.	47.8	1.804
**8**	$MnCl_2$	n.c.	n.c.	n.d.	n.d.	47.8	1.804
**9**	Mn(0)	n.c.	n.c.	n.d.	n.d.	47.8	1.804
**10**	$ZnSO_4.7H_2O$	n.c.	n.c.	n.d.	n.d.	47.8	1.804
**11**	$AlCl_3.6H_2O$	n.c.	n.c.	n.d.	n.d.	47.8	1.804
**12**	$MgCl_2$	n.c.	n.c.	n.d.	n.d.	47.8	1.804
**13**	$CuCl_2$	n.c.	n.c.	n.d.	n.d.	47.8	1.804
**14**	$CrCl_3$	n.c.	n.c.	n.d.	n.d.	47.8	1.804
**15**	/	n.c.	n.c.	n.d.	n.d.	47.8	1.804

[a] The yields were determined relative to dimethyl sulfone used as an internal standard (integral set to 6 H, concentration inside the NMR tube reported in the last column).

[b] The proton of **PEP** at 5.21 ppm was chosen for quantification to minimize the loss of integration due to the ^1H water suppression method (for details, see Fig. S11).

D. Optimization – variation of the temperature and the reaction time

An aqueous stock solution of **PEP** (48 mM), $Na_4O_7P_2$ (2 equiv), and $NaHCO_3$ (5 equiv) was freshly prepared for 21 reactions as follows: **PEP** (48 mM, 210 mg), $Na_4O_7P_2$ (2 equiv, 535 mg) and $NaHCO_3$ (5 equiv, 424 mg) were dissolved in 21 mL of MQ H_2O. After the addition of H_2O, gas bubbles were observed, and the mixture was vortexed until dissolution of the remaining $Na_4O_7P_2$ powder. Then, Eppendorf vials were charged with the metal catalyst (1 equiv), and 1 mL of the freshly prepared stock solution was added to each reaction. To perform the reaction under air without evaporation of the solution under heating, a needle was introduced into the lid of the Eppendorf vial (Fig. S10A). Samples were placed on a pre-tempered thermoshaker and incubated for different reaction times at different temperatures (see Table S4) with a speed of 800 rpm. For this screening, 200 µL, instead of 150 µL, of the thiolate/phosphate solution was used to precipitate the metals (Table S4, entries 1-20). After the indicated time, the **GP** for NMR sample preparation in the presence of metal catalysts was applied (except for entry 21). All NMR samples were analyzed by ^1H qNMR (d1 = 30 s, 8 scans).

Table S4. Optimization table with product quantification by ^1H qNMR[a].

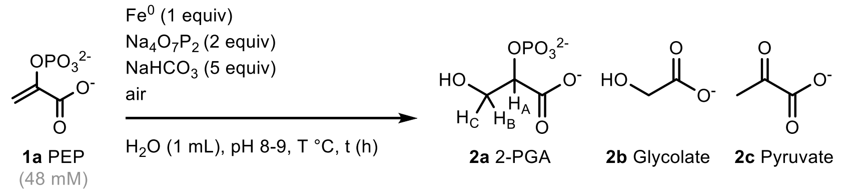

Entry	T (°C)	Time (in hours)	Int. **1a** (1 H, 5.21 ppm)[b]	Yield **1a** (%)	Int. **2a** (1 H, H_B, 3.77-3.74 ppm)	Yield **2a** (%)	Int. **2b** (2 H, 3.8 ppm)	Yield **2b** (%)	Int. **2c** (3 H, 2.22 ppm)	Yield **2c** (%)	**PEP** (mM)	**DMS** (mM)[a]
**1**		8	[redacted]									
**2**[c]		16	7.9	50.0	0.7	4.4	5.6	17.7	0.4	0.8	47.91	1.963
**3**[d]	25	24	6.3	39.8	0.8	5.1	8.1	25.6	0.4	0.8	47.91	1.963
**4**		48	[redacted]									
**5**[e]		144	9.0	54.0	1.9	11.4	5.2	15.6	0.5	1.0	47.8	1.965
**6**[c]		8	9.5	60.1	0.7	4.4	5.4	17.1	0.3	0.6	47.91	1.963
**7**[d]		16	7.8	49.3	1.7	10.8	5.8	18.3	0.6	1.3	47.91	1.963
**8**[d]	40	24	6.8	43.0	1.7	10.8	5.4	17.1	0.5	1.1	47.91	1.963
**9**[d]		48	6.5	41.1	1.7	10.8	5.7	18.0	0.5	1.1	47.91	1.963
**10**[e]		144	5.2	32.9	1.7	10.8	6.7	21.2	0.4	0.8	47.91	1.963
**11**[c]		8	9.6	60.7	1.9	12.0	3.5	11.1	0.5	1.1	47.91	1.963
**12**[d]		16	8.3	52.5	2.4	15.2	4.4	13.9	0.7	1.5	47.91	1.963
**13**[d]	60	24	8.5	53.8	2.7	17.1	3.6	11.4	0.7	1.5	47.91	1.963
**14**[d]		48	8.9	56.3	2.4	15.2	3.7	11.7	0.8	1.7	47.91	1.963
**15**[e]		144	8.1	51.2	2.4	15.2	4.2	13.3	1.2	2.5	47.91	1.963
**16**[d]		8	10.1	63.9	2.1	13.3	3.2	10.1	1.1	2.3	47.91	1.963
**17**[d]		16	7.9	50.0	2.6	16.4	4.4	13.9	1.3	2.7	47.91	1.963
**18**[f]	75	24	6.0	37.9	3.7	23.4	4.2	13.3	1.7	3.6	47.91	1.963
**19**[f]		48	8.0	50.6	3.1	19.6	3.1	9.8	2.6	5.5	47.91	1.963
**20**[f]		144	6.7	42.4	3.3	20.9	3.5	11.1	5.0	10.5	47.91	1.963
**21**[g]	60	120	8.0	50.6	2.4	15.2	4.7	14.9	1.0	2.1	47.91	1.963

[a] The yields were determined relative to dimethyl sulfone used as an internal standard (integral set to 6 H, concentration inside the NMR tube reported in the last column). For this screening (entries 1-20), 200 µL, instead of 150 µL, of the thiolate/phosphate solution were used to precipitate the metals.

[b] The proton of **PEP** at 5.21 ppm was chosen for quantification to minimize the loss of integration due to the ^1H water suppression method (for details, see Fig. S11).

[c] No precipitates were observed at the end of the reaction; the solution was light blue-violet. Sometimes trace amounts of white precipitates can be formed.

[d] No precipitates were observed at the end of the reaction; the solution was yellowish (see Fig. S10B). Sometimes trace amounts of white precipitates can be formed.

[e] Some white precipitates at the end of the reaction; the solution was yellowish (see Fig. S10B).

[f] Some orange-brown precipitates at the end of the reaction; the color of the solution was yellow-orange.

[g] To verify the effect of precipitation of metals by the addition of the thiolate/phosphate solution increasing the pH of the reaction mixture (pH 13-14), metals were removed by a Chelex ion exchange resin (Chelex® 100 sodium form). After centrifugation, the Chelex was added (roughly 150 mg of solid) to the supernatant, and the sample was shaken for 30 minutes at room temperature. The sample was centrifuged, and 500 µL of the supernatant was transferred to an NMR tube with 50 µL of a 23.6 mM stock solution of DMS and 50 µL of a 77.8 mM stock solution of phosphonoacetate. The sample was subsequently analyzed by 1H qNMR (d1 = 30 s, 8 scans, see Fig. S14B).

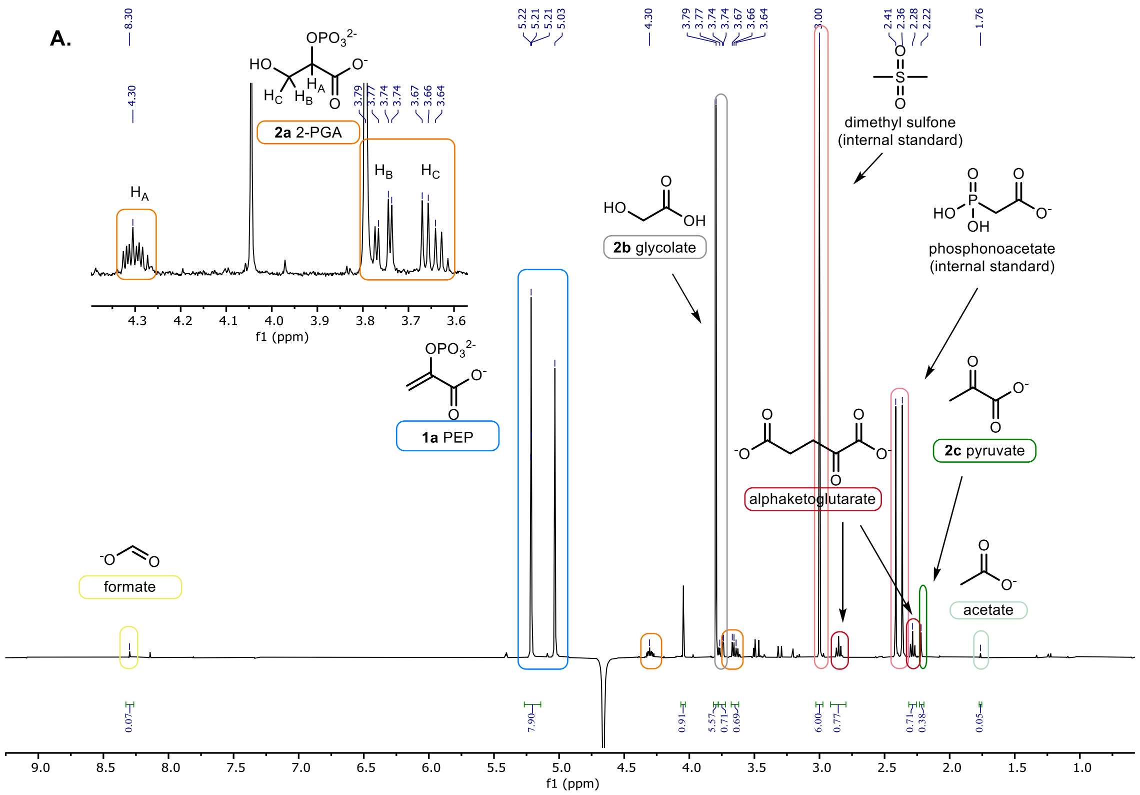

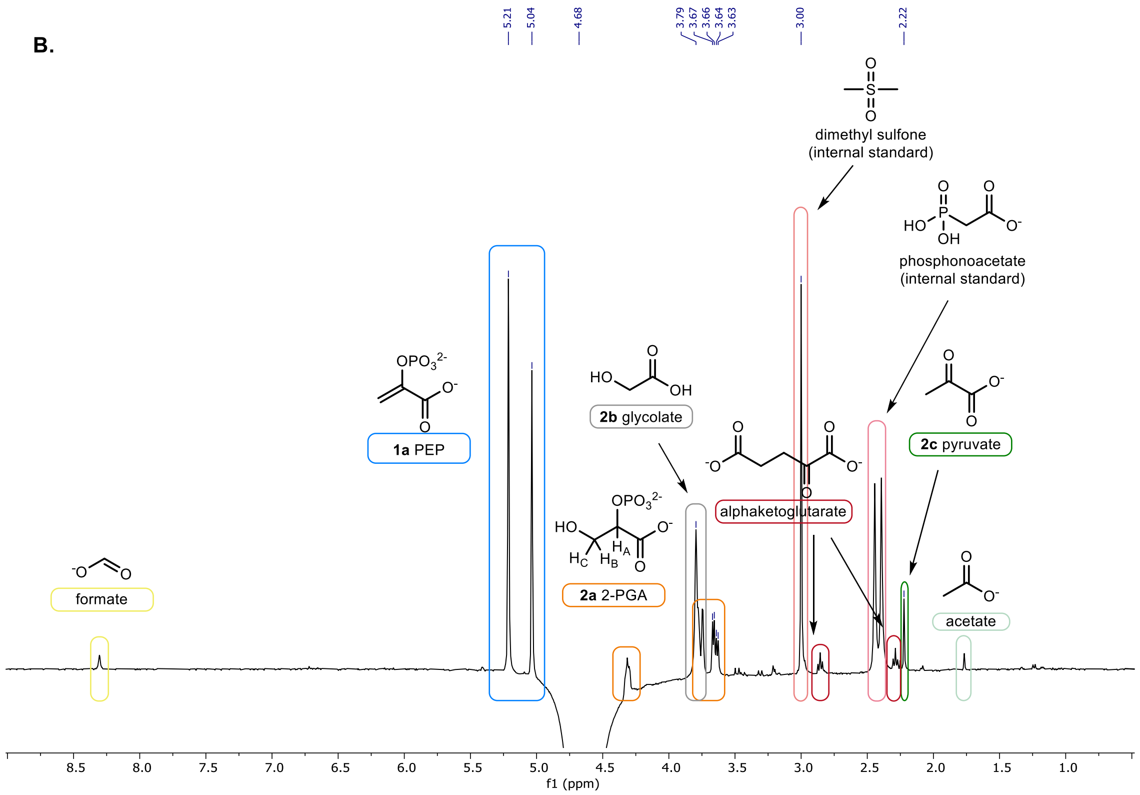

Figure S14. (A) Typical ^{1}H NMR spectrum of the reaction mixture for a reaction which was run at room temperature. Example of Table S4-Entry 2 (pH 13-14 due to the addition of the thiolate/phosphate solution to precipitate metals). (B) ^{1}H NMR spectrum of Table S4-Entry 21 after removal of metals using a Chelex ion exchange resin. The same products were detected compared to the work-up with the thiolate/phosphate solution, but the removal of the metals is usually less efficient resulting in a significant broadening of the signals which makes sometimes the quantification more difficult.

E. Optimization – Variation of the different parameters

Three aqueous stock solutions of **PEP** (48 mM), $Na_4O_7P_2$ (2 equiv) and $NaHCO_3$ (5 equiv) were freshly prepared for the following screening as follows: (1) **PEP** (48 mM, 40 mg), $Na_4O_7P_2$ (2 equiv, 102 mg) and $NaHCO_3$ (5 equiv, 81 mg) were dissolved in 4 mL of MQ H_2O for entries 1-4 (see Table S5); (2) **PEP** (48 mM, 30 mg), and $NaHCO_3$ (5 equiv, 61 mg) were dissolved in 3 mL of MQ H_2O for entries 5-7 (see Table S5); (3) **PEP** (48 mM, 30 mg), and $Na_4O_7P_2$ (2 equiv, 77 mg) were dissolved in 3 mL of MQ H_2O for entries 8-10 (see Table S5). After the addition of H_2O, gas bubbles were observed, and the mixture was vortexed until the dissolution of the remaining $Na_4O_7P_2$ powder (for stock solutions 1, 3). Then, Eppendorf vials were charged with the metal catalyst (x equiv, see Table S5), and 1 mL of the freshly prepared stock solution was added to the associated reaction (i.e., stock 1: entries 1-4 / stock 2: entries 5-7 / stock 3: entries 8-10). For entry 11, a stock solution of **PEP** (9.6 mM), $Na_4O_7P_2$ (10 equiv, 25 mg) and $NaHCO_3$ (25 equiv, 20 mg) was prepared in 1 mL of MQ H_2O. Then, the Eppendorf vial was charged with the metal catalyst (5 equiv), and 1 mL of the freshly prepared stock solution was added to the reaction. To perform the reaction under air without evaporation of the solution under heating, a needle was introduced into the lid of the Eppendorf vial (Fig. S10A). Samples were placed on a pre-tempered thermoshaker, and incubated for a reaction time of 16 h at 75 °C with a speed of 800 rpm. After the indicated time, the **GP** for NMR sample preparation in the presence of metal catalysts was applied. All NMR samples were analyzed by ^1H qNMR (d1 = 30 s, 8 scans).

Table S5. Optimization table with product quantification by ^1H qNMR[a].

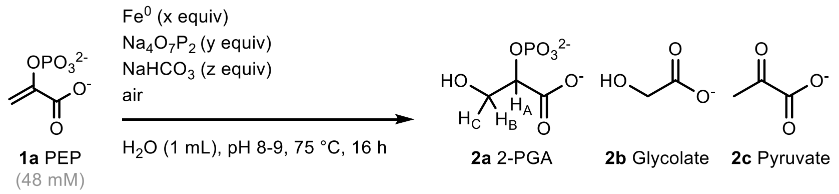

Entry	Fe^0 (x equiv)	$Na_4O_7P_2$ (y equiv)	$NaHCO_3$ (z equiv)	Int. **1a** (1 H, 5.21 ppm)[b]	Yield **1a** (%)	Int. **2a** (1 H, H_B, 3.77-3.74 ppm)	Yield **2a** (%)	Int. **2b** (2 H, 3.8 ppm)	Yield **2b** (%)	Int. **2c** (3 H, 2.22 ppm)	Yield **2c** (%)	pH[c]	PEP (mM)	DMS (mM)[a]
1[d]	0.5	2	5	10.0	59.9	1.7	10.2	4.1	12.3	2.1	4.2	9.2	47.8	1.964
2[d]	1	2	5	8.5	50.9	2.6	15.6	4.1	12.3	1.2	2.4	9.4	47.8	1.964
3[e]	2	2	5	6.7	40.1	4.1	24.6	4.1	12.3	1.8	3.6	9.4	47.8	1.964
4[e]	5	2	5	6.1	36.5	4.2	25.2	4.8	14.4	1.6	3.2	9.6	47.8	1.964
5[e]	1	0	5	13.3	79.7	traces	traces	1.2	3.6	2.8	5.6	9.2	47.8	1.964
6[d]	1	1	5	8.2	49.1	2.4	14.4	4.3	12.9	1.4	2.8	9.3	47.8	1.964
7[f]	1	5	5	9.3	55.7	2.9	17.4	3.6	10.8	1.3	2.6	9.8	47.8	1.964
8[f]	1	2	0	2.3	13.8	1.3	7.8	8.4	25.2	7.8	15.6	9.3	47.8	1.964
9[d]	1	2	2	6.4	38.3	2.7	16.2	5.2	15.6	3.2	6.4	9.3	47.8	1.964
10[f]	1	2	10	8.9	53.3	2.8	16.8	2.5	7.5	2.6	5.2	9.2	47.8	1.964
11[g]	5	10	25	0.9	26.9	0.7	21.0	1.8	26.9	0.3	3.0	-	9.6	1.963

[a] The yields were determined relative to dimethyl sulfone used as an internal standard (integral set to 6 H, concentration inside the NMR tube reported in the last column).

[b] The proton of **PEP** at 5.21 ppm was chosen for quantification to minimize the loss of integration due to the ^1H water suppression method (for details, see Fig. S11).

[c] The pH was measured at the end of the reaction before the precipitation of metals.

[d] No precipitates were observed at the end of the reaction; the solution was yellowish (see Fig. S10B). Sometimes trace amounts of white precipitates can be observed at the end of the reaction.

[e] Some brown-black precipitates at the end of the reaction; the solution was yellowish (see Fig. S10B).

[f] Some white precipitates at the end of the reaction; the solution was yellowish (see Fig. S10B).

[g] The concentration of PEP was decreased from 47.8 mM to 9.6 mM without changing the concentration of the other parameters (i.e., Fe^0 (48 mM), $NaHCO_3$ (240 mM) and $Na_4O_7P_2$ (96 mM)).

Comments regarding the possible role of bicarbonate:

- It can be preferentially adsorbed on the surface of iron complexes which would limit the adsorption of other species such as PEP whose hydrolysis to pyruvate would be favored (see the differences between the mass balances in the absence compared to in the presence of 10 equivalents of bicarbonate).
- It limits the oxidative decarboxylation of glycolate formation (see Fig. S20) following Le Chatelier principle.
- It is known to form several complexes with iron in solution which could also influence the reactivity of the iron promoter and therefore the product distribution.

F. Buffer and pH effects

An aqueous stock solution of **PEP** (48 mM), $Na_4O_7P_2$ (2 equiv), and $NaHCO_3$ (5 equiv) was freshly prepared for 7 reactions as follows: PEP (48 mM, 70 mg), $Na_4O_7P_2$ (2 equiv, 179 mg) and $NaHCO_3$ (5 equiv, 141 mg) were dissolved in 3.5 mL of MQ H_2O. After the addition of H_2O, gas bubbles were observed, and the mixture was vortexed until the dissolution of the remaining $Na_4O_7P_2$ powder. Then, Eppendorf vials were charged with the metal catalyst (1 equiv), and 0.5 mL of the freshly prepared stock solution was added in each reaction in addition to 0.5 mL of a 1 M buffer solution (see Table S6). To perform the reaction under air without evaporation of the solution under heating, a needle was introduced into the lid of the Eppendorf vial (Fig. S10A). Samples were placed on a pre-tempered thermoshaker, and incubated for a reaction time of 16 h at 75 °C with a speed of 800 rpm. After the indicated time, the **GP** for NMR sample preparation in the presence of metal catalysts was applied. All NMR samples were quantified by ^1H qNMR (d1 = 30 s, 8 scans) and, entries 4 and 6 were quantified by ^{31}P qNMR (d1 = 30 s, 16 scans).

Table S6. Buffer and pH variation (n.d. = not detected / n.c. = observed but not calculated).

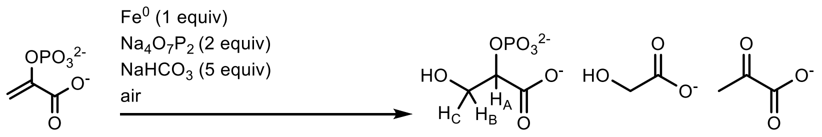

1a PEP (48 mM) · **2a** 2-PGA · **2b** Glycolate · **2c** Pyruvate

En-try	Buffer	pH	Int. **1a** (1 H, 5.21 ppm[b])	Yield **1a** (%)	Int. **2a** (1 H, H_B, 3.77-3.74 ppm)	Yield **2a** (%)	Int. **2b** (2 H, 3.8 ppm)	Yield **2b** (%)	Int. **2c** (3 H, 2.22 ppm)	Yield **2c** (%)	**PEP** (mM)	**DMS** (mM)[a]
**1**[c]	Acetate	5	n.d.	n.d.	n.d.	n.d.	0.9	2.7	31.7	62.6	48.3	1.961
**2**[d]	Phosphate	6	traces	traces	traces	traces	1.0	3.0	28.6	57.1	48.3	1.961
**3**[d]	MOPS	7	traces	traces	n.c.	n.c.	n.c.	n.c.	30.3	59.8	48.3	1.961
**4**[c,e]	Tris	8	1.2	23.9	0.1	2.0	n.c.	n.c.	n.c.	n.c.	48.3	- [e]
**5**[d]	Phosphate	8	4.3	25.5	2.4	14.2	4.4	13.0	3.9	7.7	48.3	1.961
**6**[c,e]	CHES	9	2.0	40.7	0.1	2.0	n.c.	n.c.	n.c.	n.c.	48.3	- [e]
**7**[c]	CAPS	10	5.5	32.6	0.3	1.8	0.4	1.2	2.5	4.9	48.3	1.961

[a] The yields were determined relative to dimethyl sulfone used as an internal standard (integral set to 6 H, concentration inside the NMR tube reported in the last column).

[b] The proton of **PEP** at 5.21 ppm was chosen for quantification to minimize the loss of integration due to the ^1H water suppression method (for details, see Fig. S11).

[c] Some white precipitates at the end of the reaction; the solution was yellowish (see Fig. S10B).

[d] No precipitates were observed at the end of the reaction; the solution was yellowish (see Fig. S10B). Sometimes trace amounts of white precipitates can be observed at the end of the reaction.

[e] The products of entries 4 and 6 were quantified by ^{31}P qNMR (d1 = 30 sec, 16 scans) because the ^1H characteristic peaks of buffers were overlapping with the ^1H characteristic peaks of the products or the one of DMS standard. The yields were determined relative to phosphonoacetate used as an internal standard (integral set to 1 P, 6.6 mM concentration inside the NMR tube).

G. Reaction Profile, Mass balance, and Reaction Network

An aqueous stock solution of **PEP** (48 mM), $Na_4O_7P_2$ (2 equiv), and $NaHCO_3$ (5 equiv) was freshly prepared for 14 reactions as follows: **PEP** (48 mM, 140 mg), $Na_4O_7P_2$ (2 equiv, 357 mg) and $NaHCO_3$ (5 equiv, 283 mg) were dissolved in 14 mL of MQ H_2O. After the addition of H_2O, gas bubbles were observed, and the mixture was vortexed until the dissolution of the remaining $Na_4O_7P_2$ powder. Then, Eppendorf vials were charged with the metal catalyst (1 equiv), and 1 mL of the freshly prepared stock solution was added to each reaction. To perform the reaction under air without evaporation of the solution under heating, a needle was introduced into the lid of the Eppendorf vial (Fig. S10A). Samples were placed on a pre-tempered thermoshaker and incubated for different reaction times at 75 °C (see Table S7) with a speed of 800 rpm. After the indicated time, the **GP** for NMR sample preparation in the presence of metal catalysts was applied. All NMR samples were analyzed by ^1H qNMR (d1 = 30 s, 8 scans) and ^{31}P NMR (d1 = 30 s, 128 scans).

Table S7. Time variation with product quantification by ^1H qNMR[a].

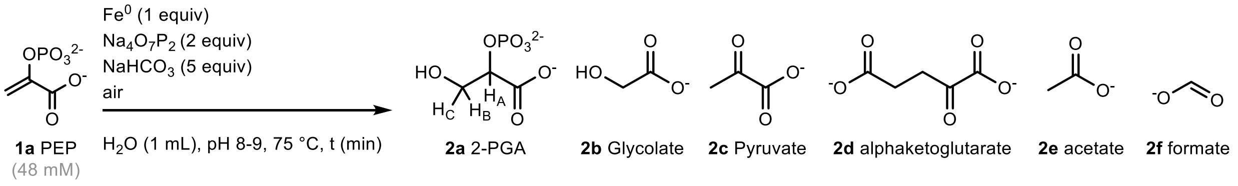

Entry	Time (min)	Int. **1a** (1 H, 5.21 ppm[b])	Yield **1a** (%)	Int. **2a** (1 H, H_B, 3.77-3.74 ppm)	Yield **2a** (%)	Int. **2b** (2 H, 3.8 ppm)	Yield **2b** (%)	Int. **2c** (3 H, 2.22 ppm)	Yield **2c** (%)	Int. **2d** (2 H, 2.85 ppm)	Yield **2d** (%)	Int. **2e** (3 H, 1.76 ppm)	Yield **2e** (%)	Int. **2f** (1 H, 8.3 ppm)	Yield **2f** (%)	**PEP** (mM)	**DMS** (mM)[a]
**1**	0	15.60	93.2	0.0	0.0	0.0	0.0	0.1	0.1	0.0	0.0	0.1	0.1	0.0	0.0	47.9	1.961
**2**	30	13.90	83.0	0.0	0.0	2.1	6.2	0.2	0.3	0.2	0.5	0.1	0.1	0.0	0.2	47.9	1.961
**3**	60	11.60	69.3	0.5	2.7	4.4	13.1	0.4	0.8	0.4	1.0	0.1	0.1	0.1	0.3	47.9	1.961
**4**	120	12.40	74.0	0.7	3.9	2.8	8.2	0.5	1.1	0.3	0.7	0.1	0.1	0.1	0.3	47.9	1.961
**5**	180	11.40	68.1	0.9	5.5	3.1	9.2	0.7	1.3	0.3	0.8	0.1	0.1	0.0	0.2	47.9	1.961
**6**	240	11.70	69.9	1.4	8.6	2.9	8.6	0.8	1.6	0.3	0.8	0.0	0.1	0.0	0.2	47.9	1.961
**7**	360	10.50	62.7	1.9	11.6	3.0	8.9	1.0	2.0	0.3	0.8	0.1	0.1	0.0	0.2	47.9	1.961
**8**	480	9.40	56.1	2.5	14.9	3.7	10.9	1.1	2.1	0.3	1.0	0.1	0.1	0.1	0.8	47.9	1.961
**9**	600	9.20	54.9	2.7	16.3	3.0	9.0	1.2	2.4	0.3	0.9	0.1	0.1	0.1	0.4	47.9	1.961
**10**	750	8.90	53.1	2.5	14.7	3.8	11.4	1.2	2.3	0.4	1.0	0.1	0.1	0.2	1.1	47.9	1.961
**11**	960	8.20	49.0	3.3	19.5	3.4	10.0	1.5	2.9	0.3	0.9	0.1	0.2	0.2	1.0	47.9	1.961
**12**	1440	8.30	49.6	3.2	19.3	3.3	10.0	1.7	3.4	0.3	0.8	0.1	0.2	0.1	0.8	47.9	1.961
**13**	2040	8.20	49.0	2.5	15.2	4.3	12.9	1.9	3.9	0.4	1.1	0.1	0.2	0.2	1.4	47.9	1.961
**14**	2880	8.00	47.8	2.9	17.1	4.1	12.4	2.4	4.7	0.3	1.0	0.1	0.3	0.2	1.4	47.9	1.961

[a] The yields were determined relative to dimethyl sulfone used as an internal standard (integral set to 6 H, concentration inside the NMR tube reported in the last column).

[b] The proton of **PEP** at 5.21 ppm was chosen for quantification to minimize the loss of integration due to the ^1H water suppression method (for details, see Fig. S11).

Comments regarding the mass balance:

- The water suppression method can affect the integration of some products of the reaction which affects the quantification of the products, especially the integration of **PEP** peaks (for details, see Fig. S11).
- As represented in Fig. S15A, the side product glycolate **2b** can oxidize to glyoxylate (due to oxidative conditions) which can subsequently oxidize to oxalate (identified by mass spectroscopy, Fig. S16D) which can decarboxylate to CO_2,[3] accompanied by a loss of a certain percentage of the mass balance.
- At the end of the reaction (e.g. Table S7-entry 13), some precipitates can be obtained which, after isolation and resuspension in H_2O followed by precipitation of metals with the addition of the thiolate/phosphate solution, contain the same reaction mixture (Fig. S15D).
- The system being heterogeneous, some of the mass can be adsorbed on the surface of the metallic iron/iron oxides which are removed by centrifugation before precipitation of the soluble iron ions in solution. This might be partly responsible for the missing mass balance.

A. Reaction network showing 3 main pathways

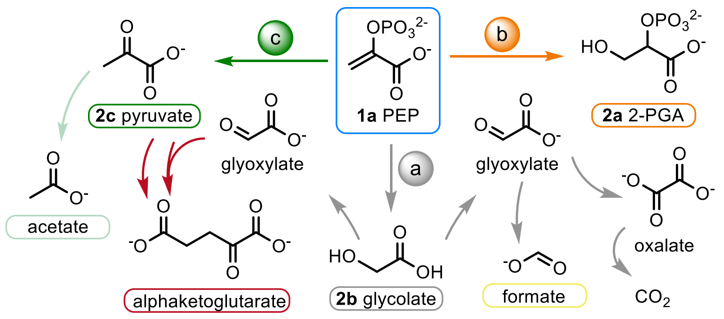

B. Reaction progress

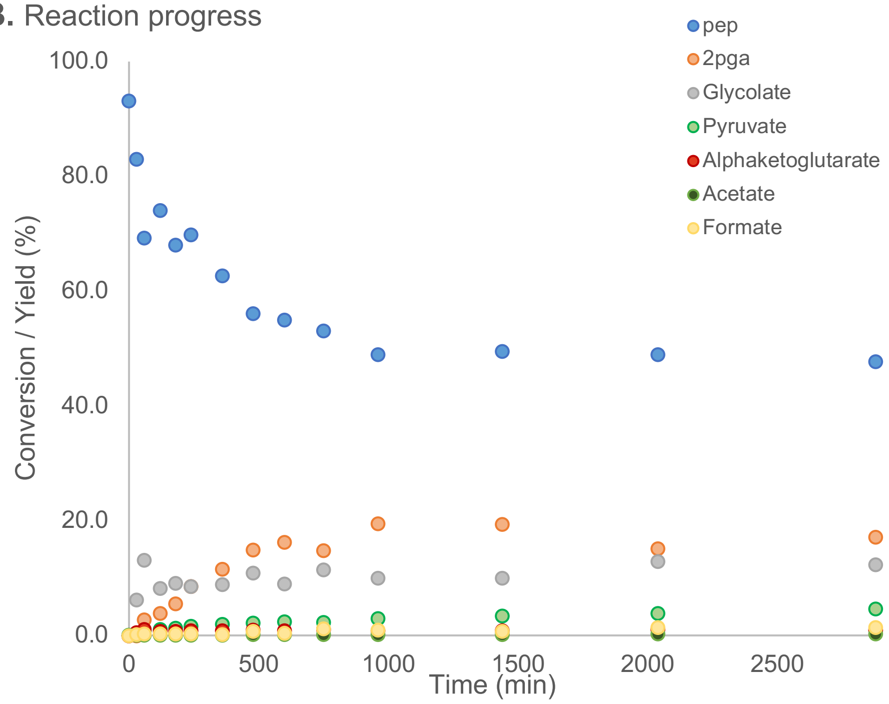

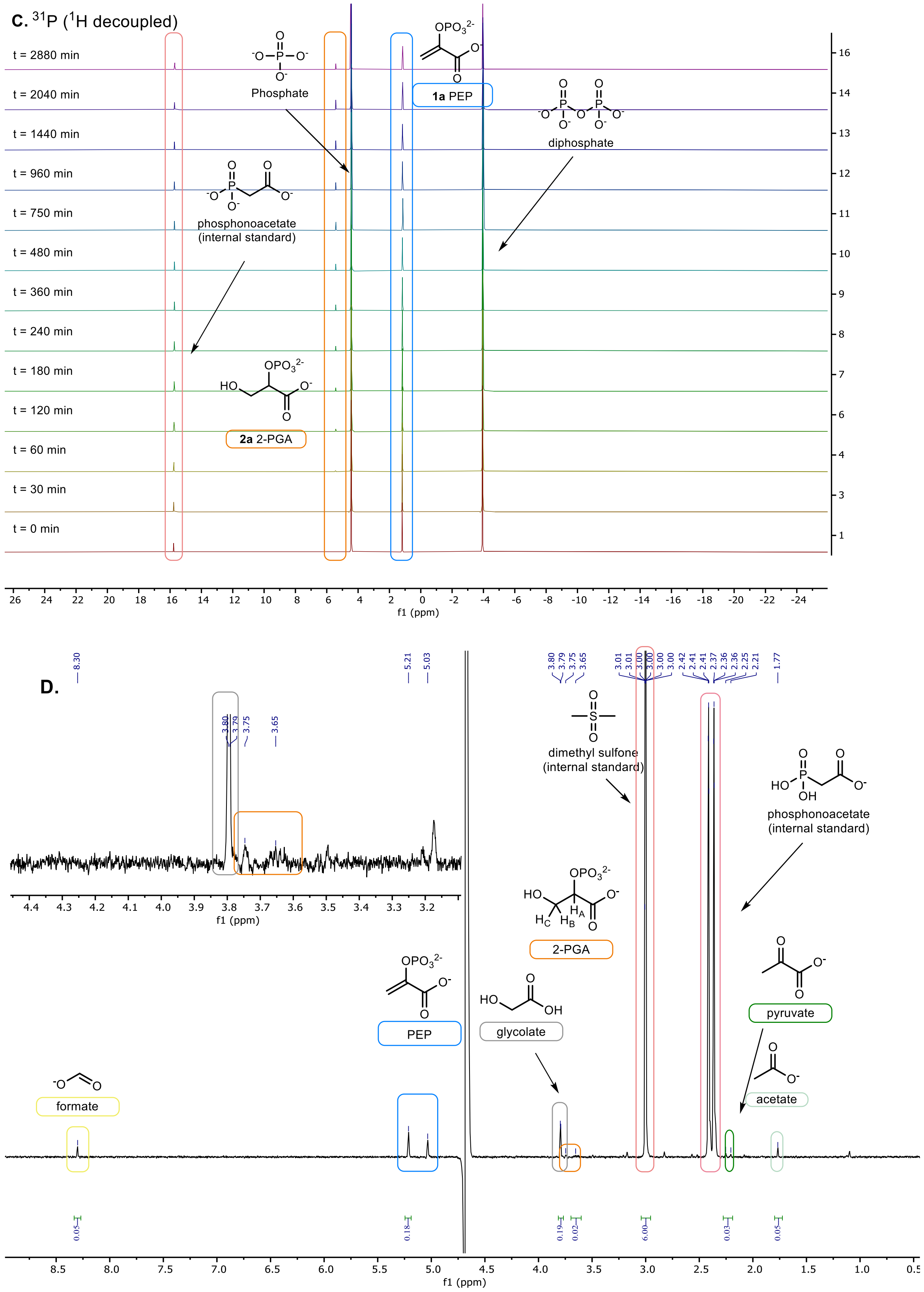

Figure S15. Reaction network with evolution profile of the main species identified by ^{1}H and ^{31}P NMR. (A) Identification of 3 main pathways (a,b, and c). (B) Reaction progress with quantification of the species by ^{1}H qNMR. (C) Reaction progress followed by ^{31}P NMR (peak of the internal standard phosphonoacetate set at 15.7 ppm) showing the formation of **2-PGA** (peak at approx. 5.5 ppm) and the decrease of **PEP** (peak at approx. 1.2 ppm) without formation of additional species (solution at pH 13-14 due to the addition of the thiolate/phosphate solution to precipitate metals which also explains the presence of a huge peak of phosphate (peak at approx. 4.5 ppm)). (D) ^{1}H NMR spectrum of the reaction mixture obtained after resuspension in H_2O for 2 hours of the precipitates obtained at the end of the reaction (in this case, Table S7-entry 13) and after subsequent precipitation of the metals by addition of the thiolate/phosphate solution.

Identification of the reaction products by mass spectroscopy

The reaction mixture was also analyzed by mass spectroscopy (HRMS). The products of the reaction network described in Fig. S15A (except formate and acetate) were also identified by HRMS (Fig. S16).

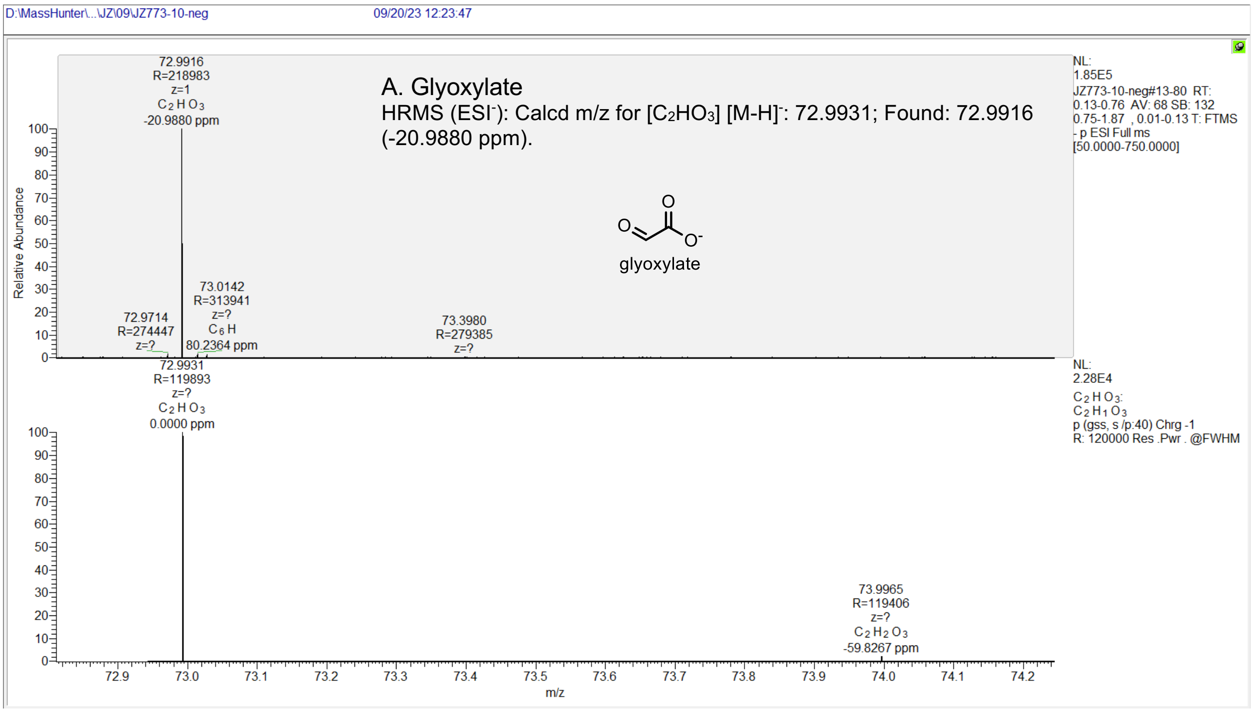

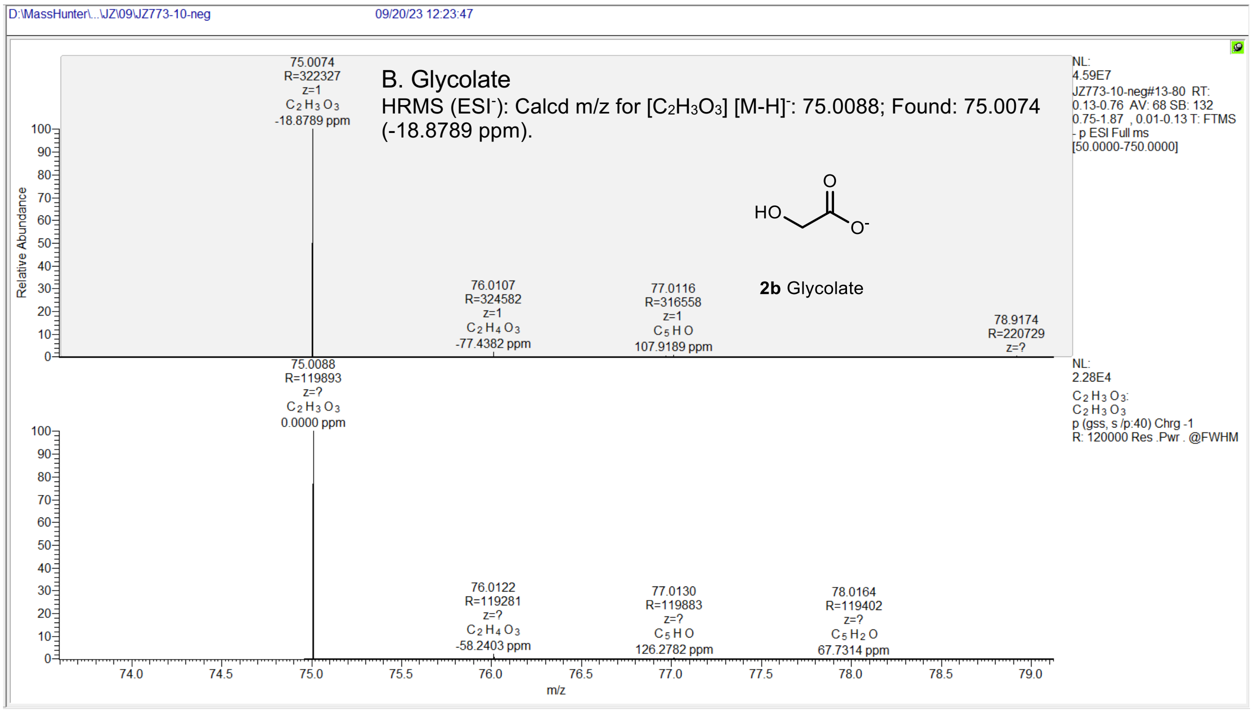

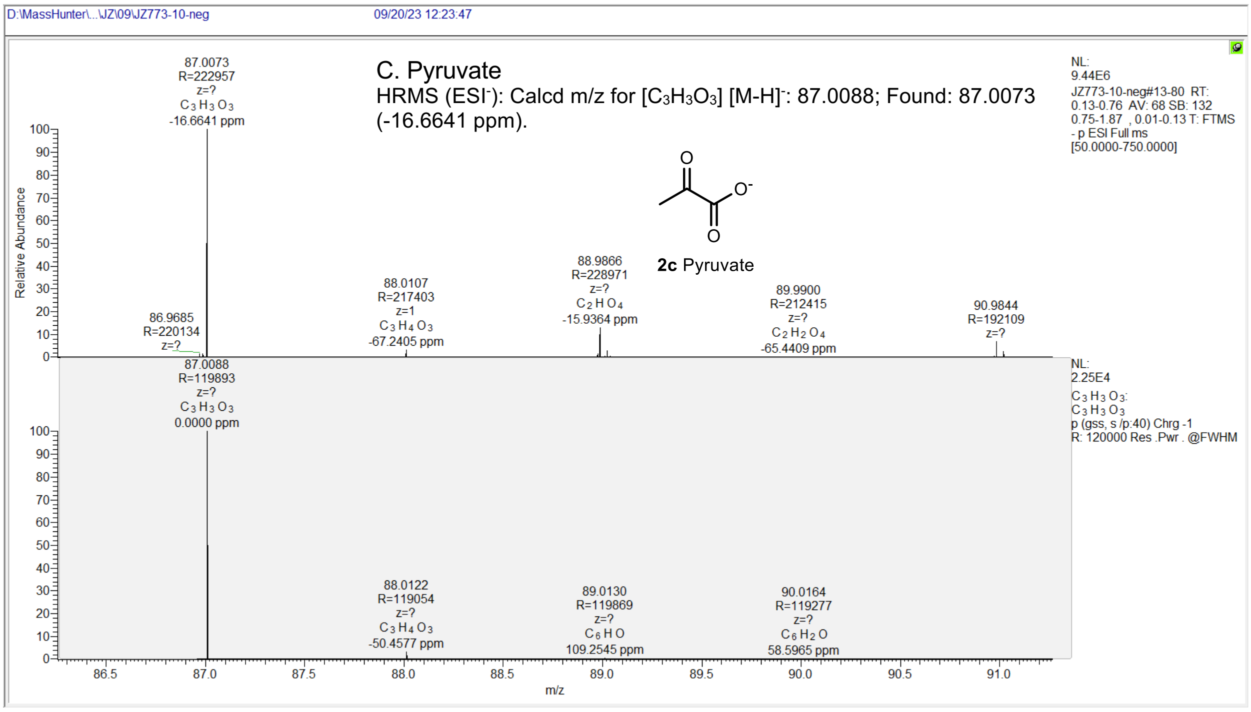
D:\MassHunter\...\JZ\09\JZ773-10-neg
09/20/23 12:23:47
C. Pyruvate
HRMS (ESI⁻): Calcd m/z for [$C_3H_3O_3$] [M-H]⁻: 87.0088; Found: 87.0073 (-16.6641 ppm).
2c Pyruvate
87.0073 R=222957 z=? $C_3H_3O_3$ -16.6641 ppm
86.9685 R=220134 z=?
88.0107 R=217403 z=1 $C_3H_4O_3$ -67.2405 ppm
88.9866 R=228971 z=? C_2HO_4 -15.9364 ppm
89.9900 R=212415 z=? $C_2H_2O_4$ -65.4409 ppm
90.9844 R=192109 z=?
87.0088 R=119893 z=? $C_3H_3O_3$ 0.0000 ppm
88.0122 R=119054 z=? $C_3H_4O_3$ -50.4577 ppm
89.0130 R=119869 z=? C_6HO 109.2545 ppm
90.0164 R=119277 z=? C_6H_2O 58.5965 ppm
NL: 9.44E6 JZ773-10-neg#13-80 RT: 0.13-0.76 AV: 68 SB: 132 0.75-1.87 , 0.01-0.13 T: FTMS - p ESI Full ms [50.0000-750.0000]
NL: 2.25E4 $C_3H_3O_3$: $C_3H_3O_3$ p (gss, s /p:40) Chrg -1 R: 120000 Res .Pwr . @FWHM
Relative Abundance
m/z

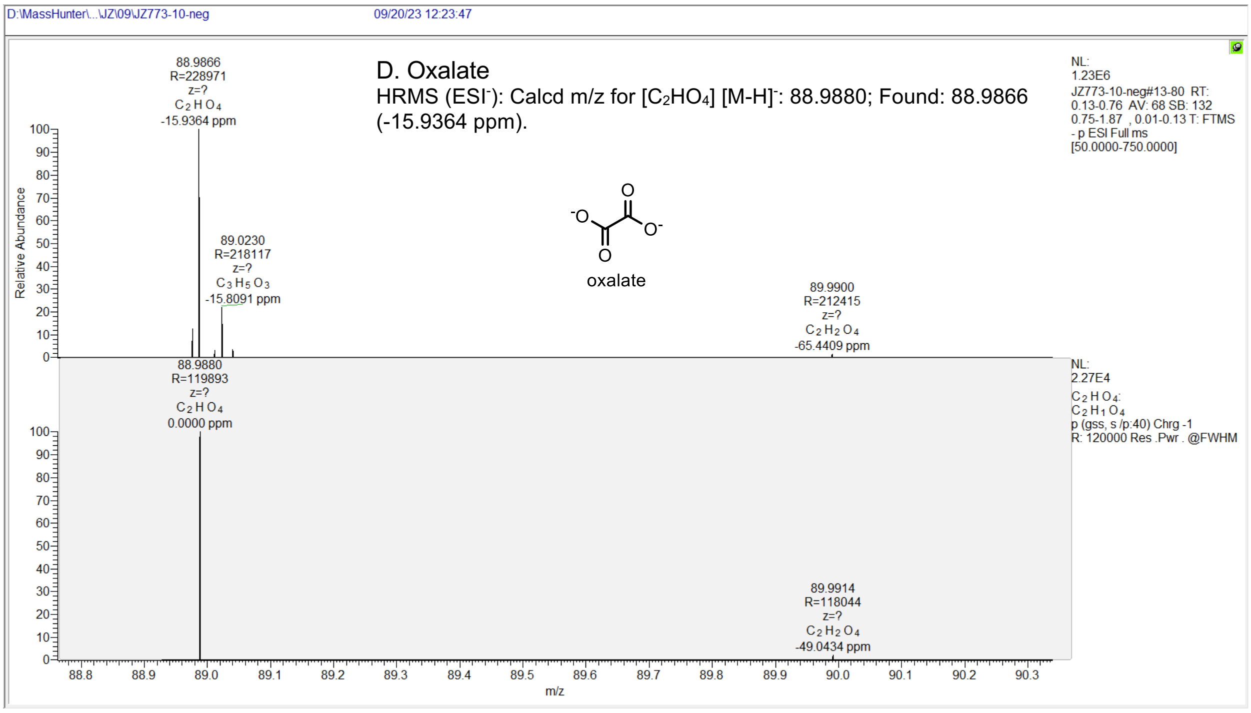
D:\MassHunter\...\JZ\09\JZ773-10-neg
09/20/23 12:23:47
D. Oxalate
HRMS (ESI⁻): Calcd m/z for [C_2HO_4] [M-H]⁻: 88.9880; Found: 88.9866 (-15.9364 ppm).
oxalate
88.9866 R=228971 z=? C_2HO_4 -15.9364 ppm
89.0230 R=218117 z=? $C_3H_5O_3$ -15.8091 ppm
89.9900 R=212415 z=? $C_2H_2O_4$ -65.4409 ppm
88.9880 R=119893 z=? C_2HO_4 0.0000 ppm
89.9914 R=118044 z=? $C_2H_2O_4$ -49.0434 ppm
NL: 1.23E6 JZ773-10-neg#13-80 RT: 0.13-0.76 AV: 68 SB: 132 0.75-1.87 , 0.01-0.13 T: FTMS - p ESI Full ms [50.0000-750.0000]
NL: 2.27E4 C_2HO_4: $C_2H_1O_4$ p (gss, s /p:40) Chrg -1 R: 120000 Res .Pwr . @FWHM
Relative Abundance
m/z

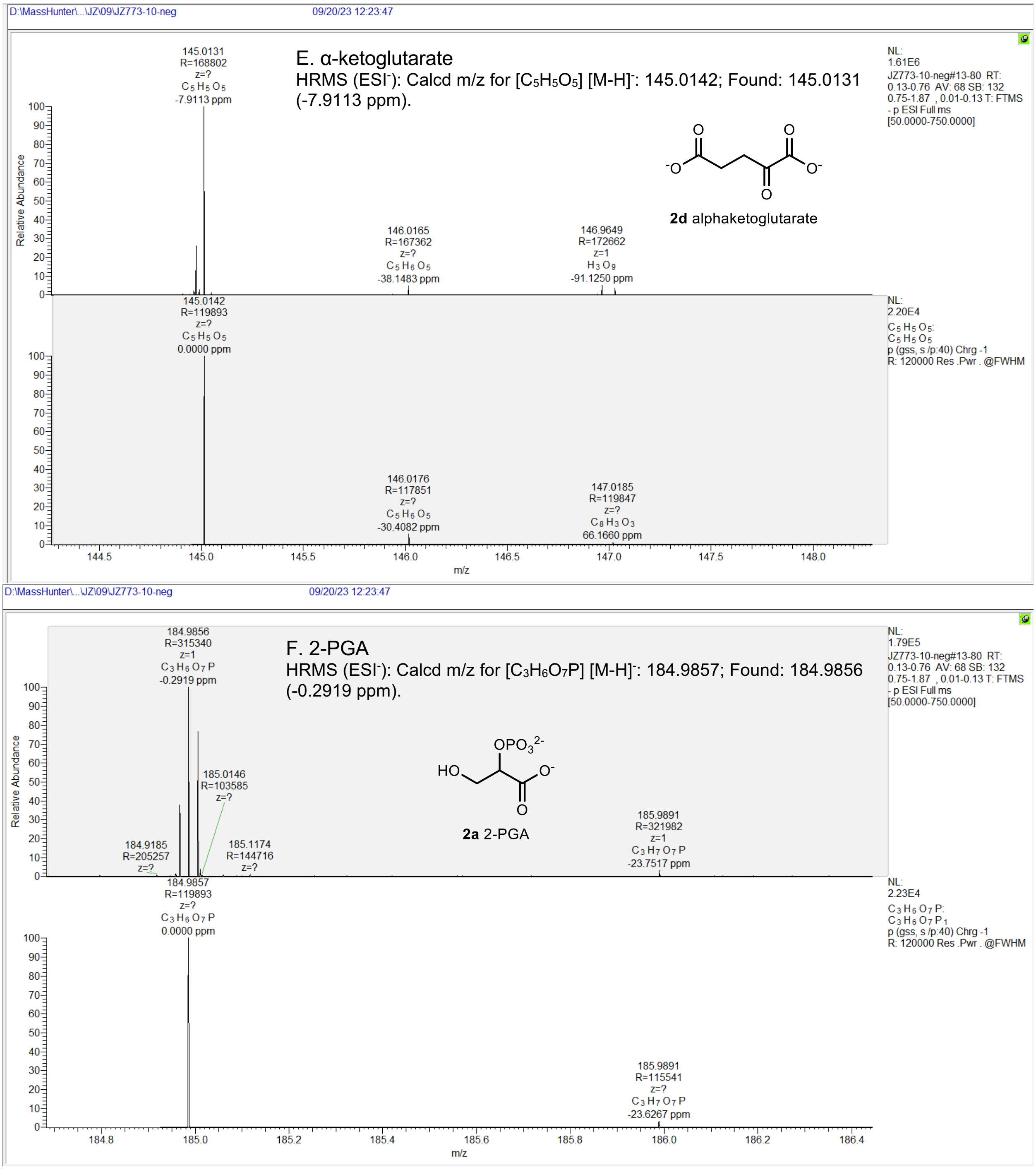

Figure S16. Identification of the reaction mixture by mass spectroscopy (HRMS). (A) Glyoxylate, (B) glycolate, (C) pyruvate, (D) oxalate, (E) α-ketoglutarate, and (F) 2-PGA were detected.

H. Investigating the formation of the side products of the reaction

1) Stability and decomposition of the products of the reaction

The main products of the reaction (**2a**, **2b,** and **2c**) were subjected to the standard reaction conditions: substrate (48 mM), Fe^0 (1 equiv), $Na_4O_7P_2$ (2 equiv), $NaHCO_3$ (5 equiv), 75 °C, 16 h with a speed of 800 rpm. After the indicated time, the **GP** for NMR sample preparation in the presence of metal catalysts was applied. For glycolate (subsection b) and pyruvate (subsection c), the internal standard phosphonoacetate was not added in the preparation of the NMR tubes (total volume in the NMR tube: 550 µL instead of 600 µL). NMR samples were analyzed qualitatively by a standard water suppression sequence (noesygppr1d, 400 MHz, ns = 16).

a) Study of **2-PGA** (**2a**) stability

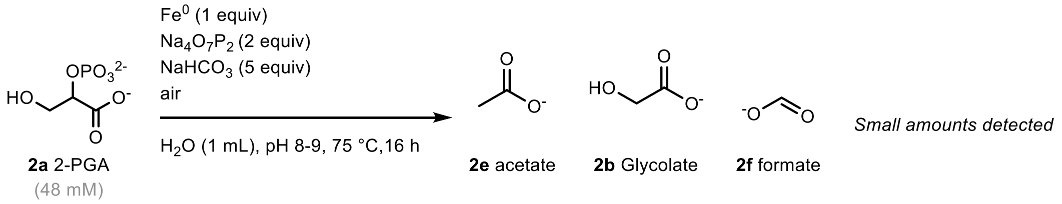

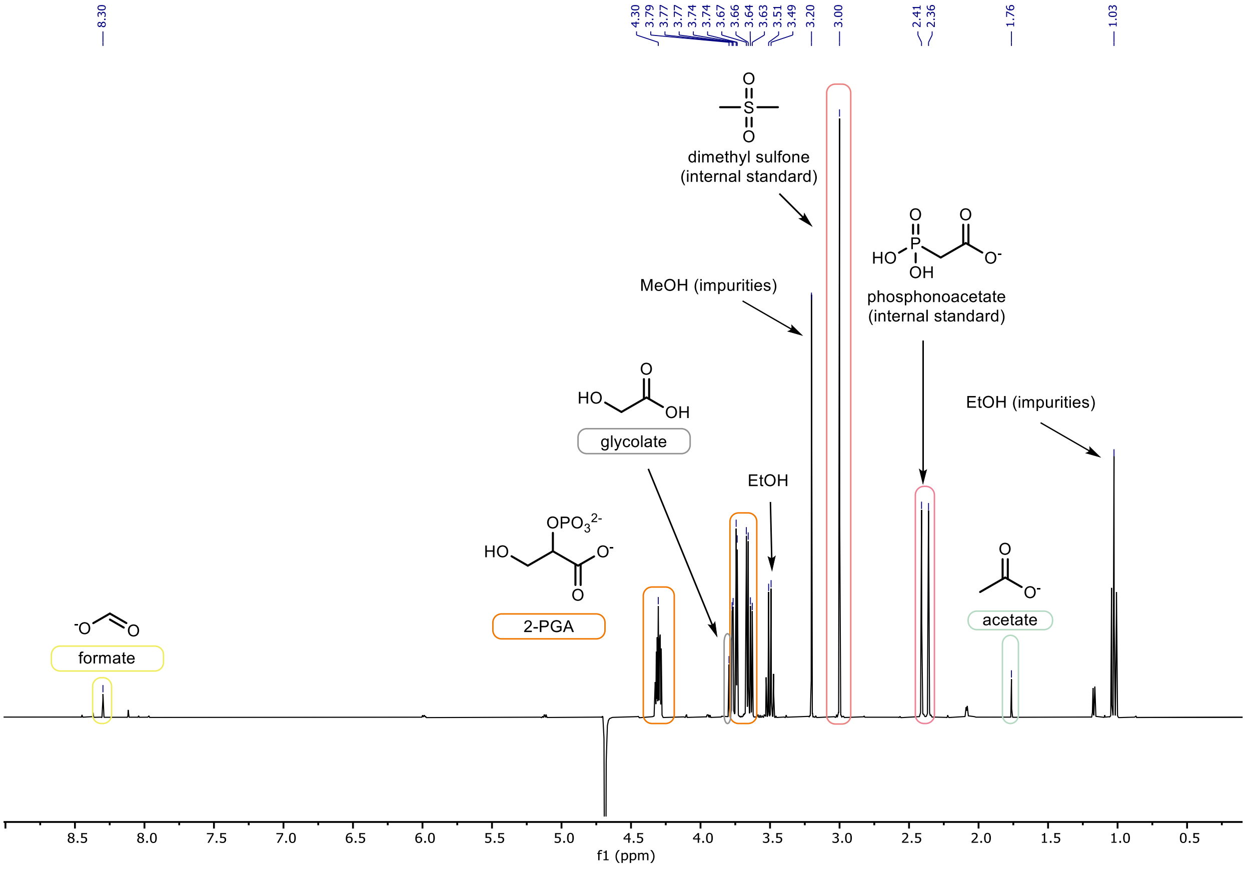

Figure S17. ^{1}H NMR study of **2-PGA** stability under our standard conditions. **2-PGA** is stable in our standard conditions (pH 8-9) with only small degradations to glycolate, acetate, and formate.

b) Study of **Glycolate** (**2b**) stability and its oxidation to glyoxylate

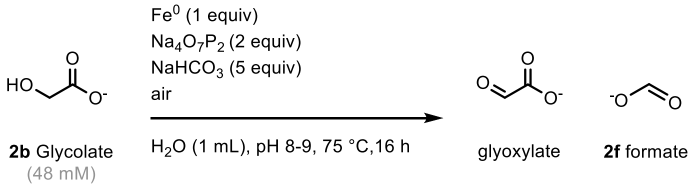

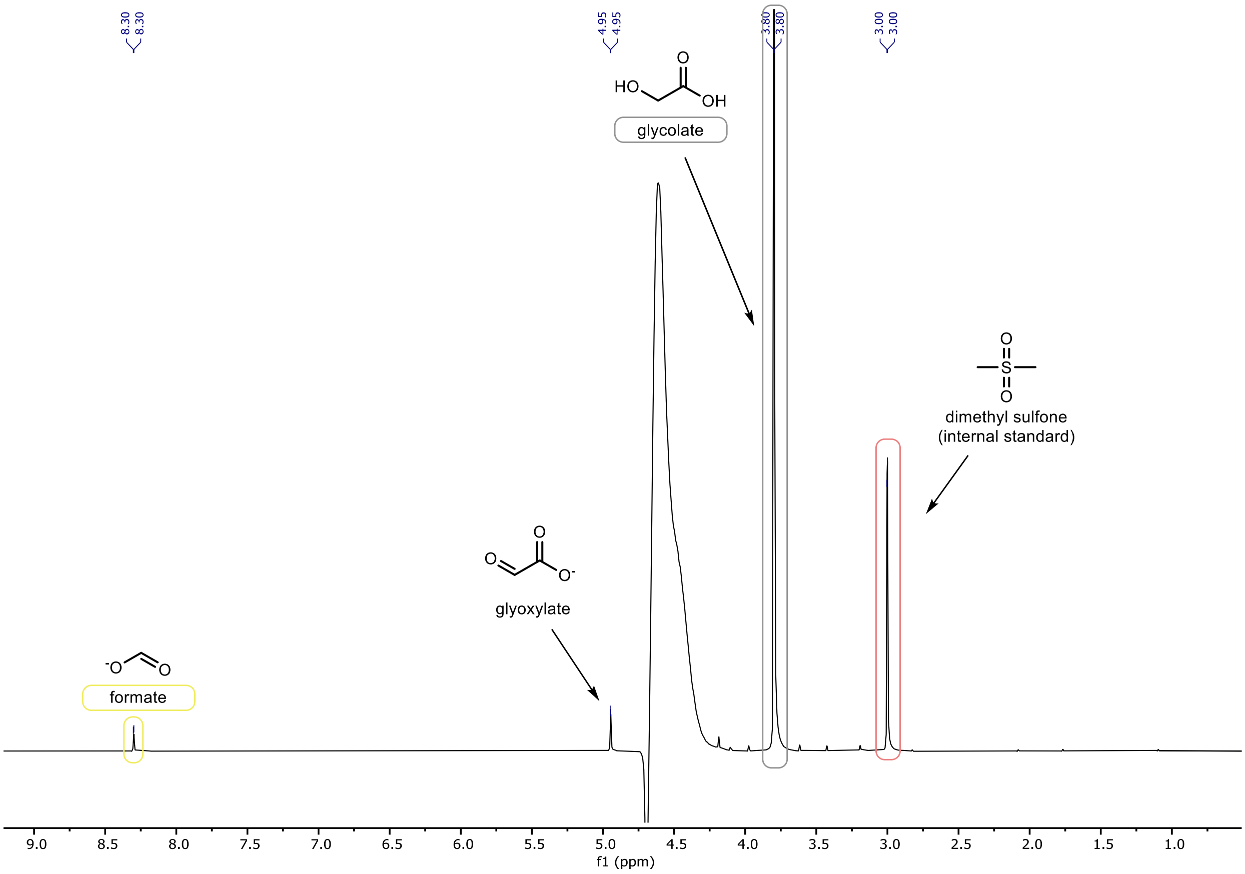

Figure S18. ^{1}H NMR study of glycolate (**2b**) stability under our standard conditions. **2b** is stable in our standard conditions (pH 8-9) with the detection of glyoxylate, the oxidized product, along with a small decomposition to formate.

c) Study of **Pyruvate** (**2c**) stability

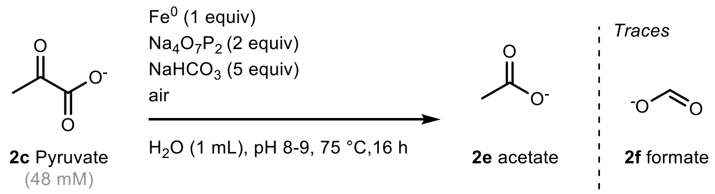

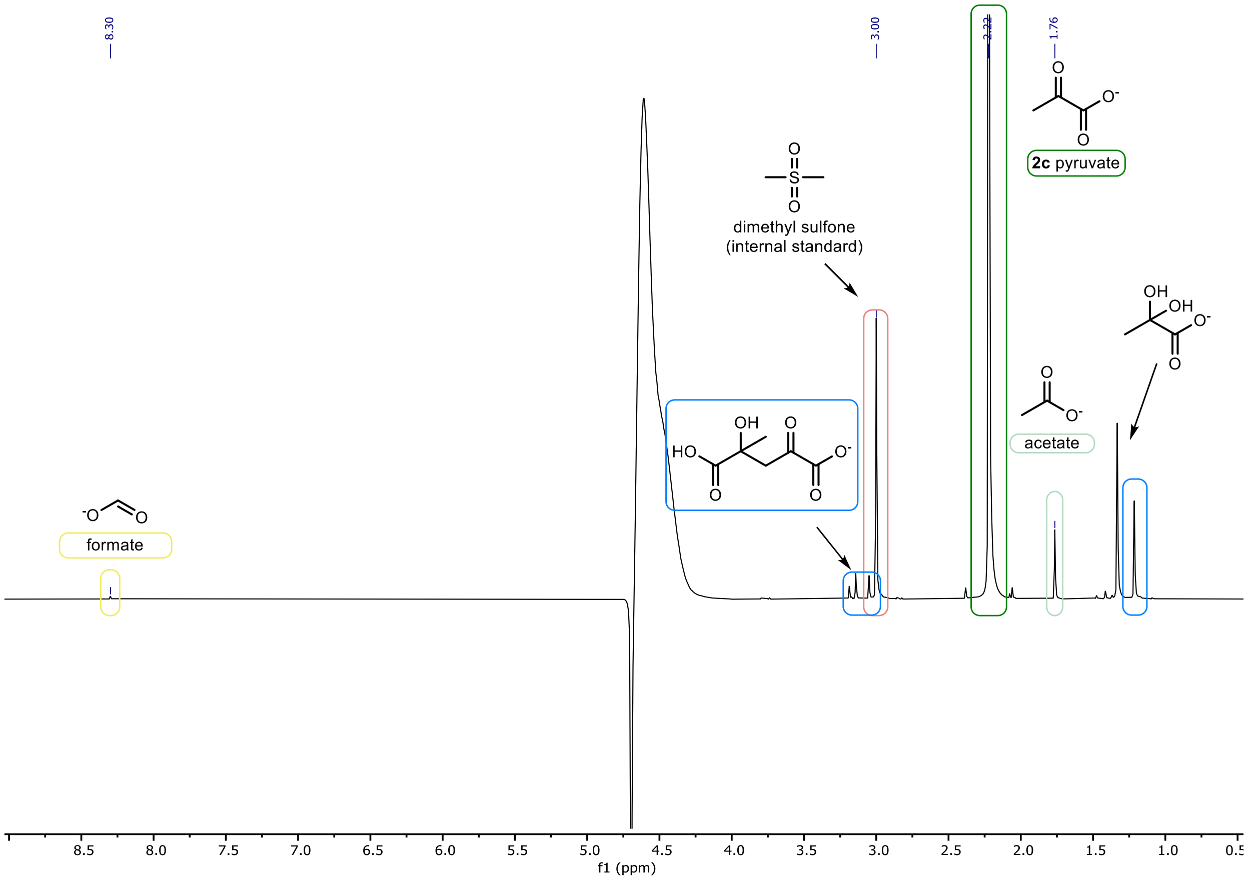

Figure S19. ^{1}H NMR study of pyruvate (**2c**) stability under our standard conditions. **2c** is stable in our standard conditions (pH 8-9) with detection of the formation of parapyruvate (in blue) and the decomposition of pyruvate to acetate and small amounts of formate.

2) Study of α-ketoglutarate formation

The formation of α-ketoglutarate (**2d**) was already reported in the literature in a series of reactions starting from glyoxylate and pyruvate in the presence of a reducing agent.[3,4] In our standard conditions, glycolate can be oxidized to glyoxylate (Fig. S18). To verify that the formation of **2d** goes through the same mechanism, we performed two reactions, one starting from sodium pyruvate (5.3 mg, 48 mM) and glyoxylic acid monohydrate (5.5 mg, 60 mM, 1.2 equiv) (based on what is reported in the literature, Fig. 20A), and another one starting from sodium pyruvate (5.3 mg, 48 mM) and glycolic acid (3.7 mg, 48 mM, 1 equiv) (Fig. 20B), both in our standard conditions: Fe^0 (1 equiv), $Na_4O_7P_2$ (2 equiv), $NaHCO_3$ (5 equiv), 75 °C, 16 h with a speed of 800 rpm. After the indicated time, the **GP** for NMR sample preparation in the presence of metal catalysts was applied. NMR samples were analyzed by a standard water suppression pulse sequence (noesygppr1d, 400 MHz, d1 = 30 s, ns = 8).

A.

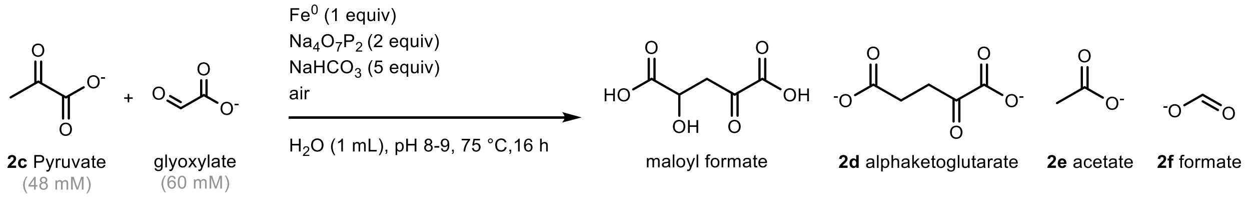

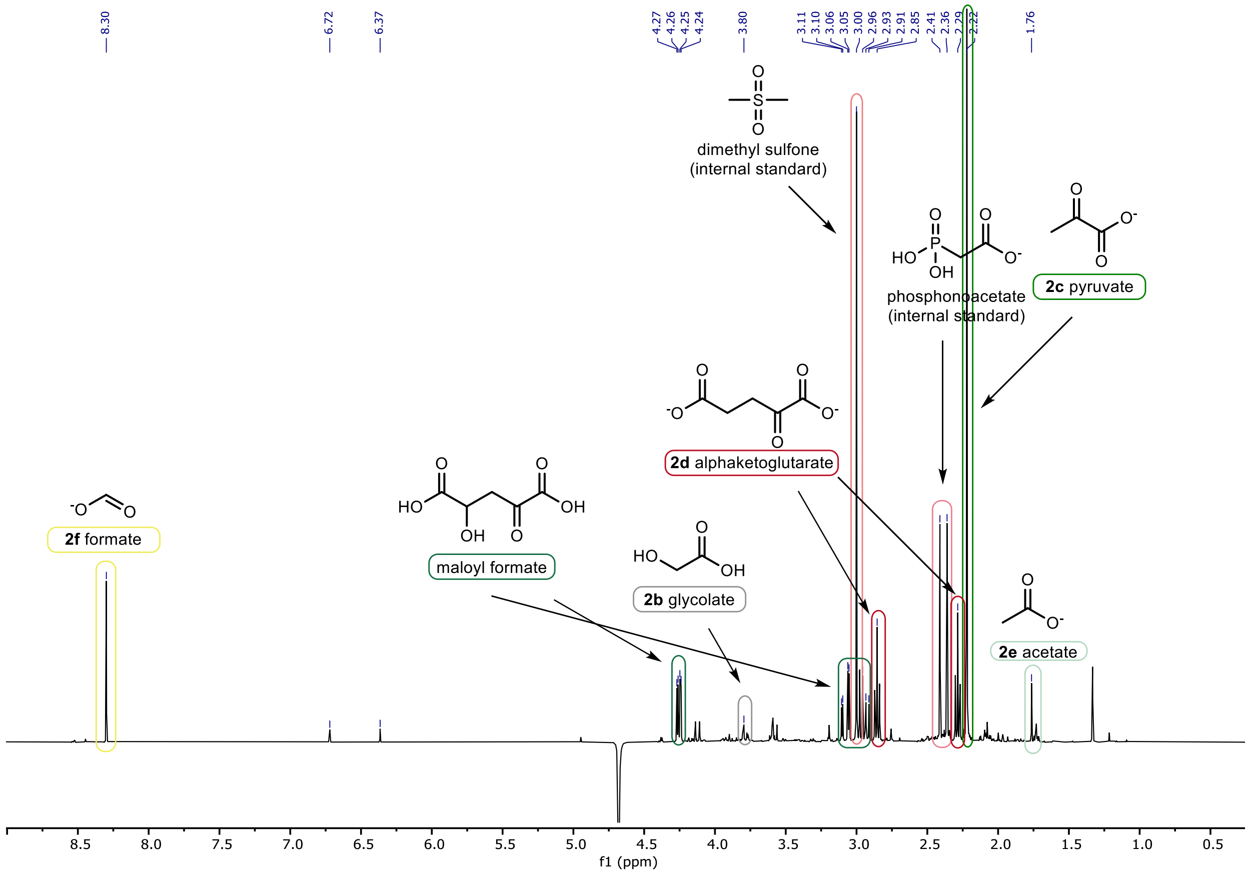

B.

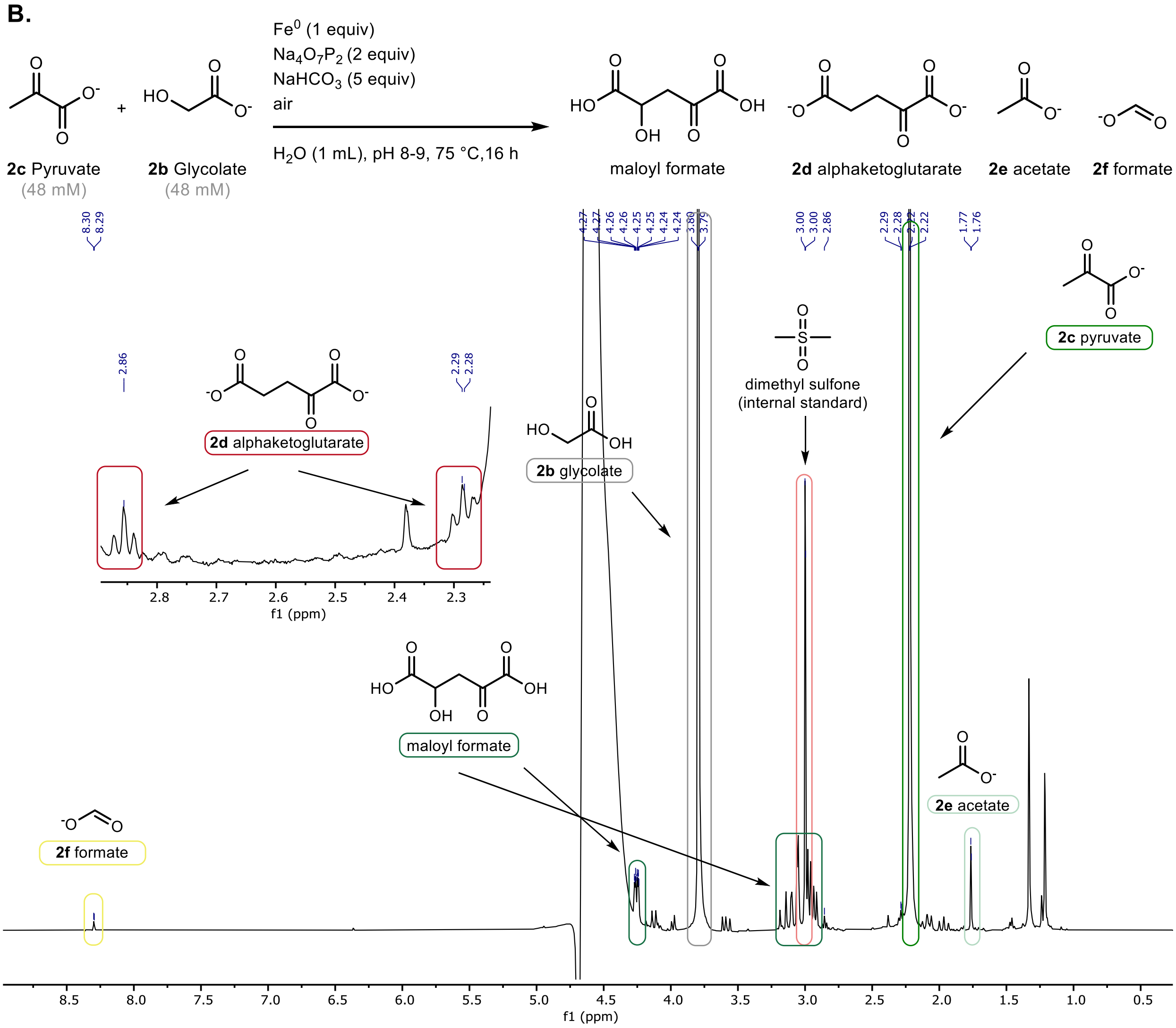

C.

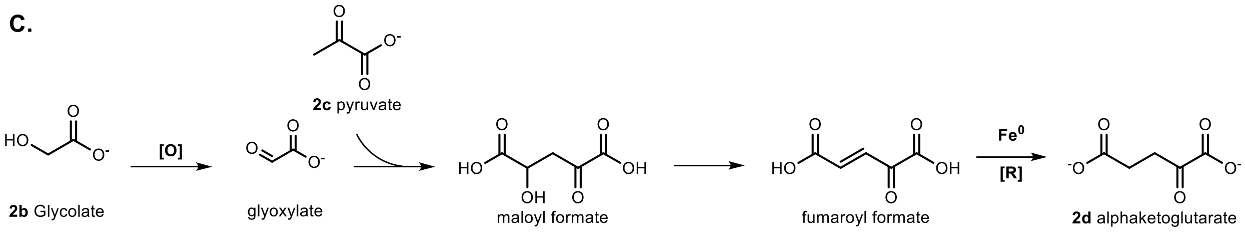

Figure S20. Study of α-ketoglutarate (**2d**) formation. (A) Reaction of pyruvate (**2c**) and glyoxylate with the associated ^1H NMR analysis of the reaction mixture (pH 13-14 due to the addition of the thiolate/phosphate solution to precipitate metals). (B) Reaction of **2c** and glycolate **2b** with the associated ^1H NMR analysis of the reaction mixture (pH 13-14 due to the addition of the thiolate/phosphate solution to precipitate metals). (C) Proposed mechanism for the formation of **2d** from **2b** and **2c** in our system. Without the presence of Fe^0 in the reaction mixture, the products of the reaction were maloyl formate and fumaroyl formate as previously reported at pH 9.5 when mixing pyruvate and glyoxylate in H_2O.[3,4]

3) Study of glycolate formation

a) Study of a model reaction using H_2O_2 to study the formation of glycolate (**2b**) starting from **PEP**

The formation of **2b** was previously reported from the degradation of **PEP** in oxidative conditions.[5] To study the formation of **2b**, we used H_2O_2 (30% w/w; 5.4 µL, 1 equiv) instead of Fe^0 in our standard conditions: **PEP** (48 mM), $Na_4O_7P_2$ (2 equiv), $NaHCO_3$ (5 equiv), at 75 °C for a reaction time of 2 h. An aqueous stock solution of **PEP** (48 mM), $Na_4O_7P_2$ (2 equiv) and $NaHCO_3$ (5 equiv) was freshly prepared for 18 reactions as follows: **PEP** (48 mM, 100 mg), $Na_4O_7P_2$ (2 equiv, 255 mg) and $NaHCO_3$ (5 equiv, 202 mg) were dissolved in 9.95 mL of MQ H_2O. After the addition of H_2O, gas bubbles were observed, and the mixture was vortexed until the dissolution of the remaining $Na_4O_7P_2$ powder. Then, H_2O_2 (30% w/w; 54 µL, 1 equiv) was added to the reaction mixture and 0.5 mL of the freshly prepared stock solution was added in 18 different Eppendorf vials to run them in parallel at 75°C. To perform the reaction under air without evaporation of the solution under heating, a needle was introduced into the lid of the Eppendorf vial (Fig. S10A). Samples were placed on a pre-tempered thermoshaker and incubated for different reaction times at 75 °C (see below) with a speed of 800 rpm. After the indicated time, 500 µL of the reaction mixture was transferred to an NMR tube, 50 µL of a 23.6 mM stock solution of **DMS** (dimethyl sulfone in D_2O), and, 50 µL of a 77.8 mM stock solution of phosphonoacetate as internal standards were added. NMR samples were analyzed by a standard water suppression sequence (noesygppr1d, 400 MHz, ns = 16).

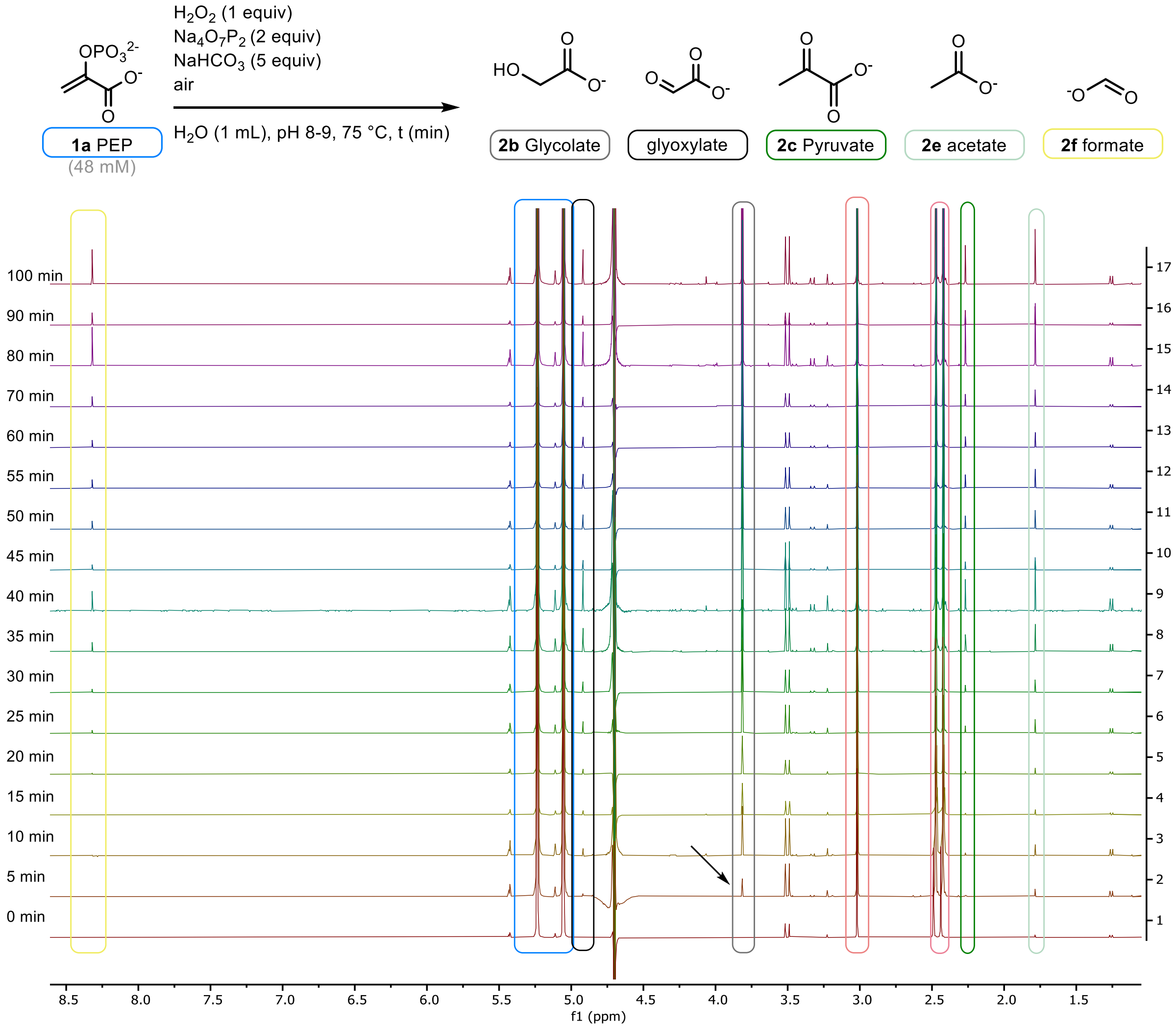

Figure S21. Study of glycolate (**2b**) formation. ^1H NMR spectra of the reaction mixture after the indicated time at 75 °C. DMS (internal standard) at 3.0 ppm (pink) and phosphonoacetate (internal standard) at approx. 2.5 ppm (doublet, pink). **PEP** (blue) decomposes over time to glycolate (**2b**, grey) which was detected after 5 min at 75 °C, followed by the formation of acetate (light green), glyoxylate (dark), pyruvate (green), and formate (yellow).

b) Study of hydroxypyruvate decomposition and proposed mechanism for the formation of **2b** from **PEP**

The epoxidation of alkenes by H_2O_2 was previously reported in concentrated $NaHCO_3$ solution.[6] We hypothesized that **2b** could be obtained through alkene epoxidation followed by the subsequent decomposition of the in-situ generated active intermediate hydroxypyruvate (Fig. S22B). To verify this hypothesis, lithium β-hydroxypyruvate hydrate (48 mM) was subjected to our standard conditions: $Na_4O_7P_2$ (2 equiv), $NaHCO_3$ (5 equiv), with or without Fe^0 (1 equiv) or with H_2O_2 (30% w/w; 5.4 µL, 1 equiv) at 75 °C for a reaction time of 2 h with a speed of 800 rpm. When the reaction was run in the presence of Fe^0, the **GP** for NMR sample preparation in the presence of metal catalysts was applied. NMR samples were analyzed by a standard water suppression sequence (noesygppr1d, 400 MHz, ns = 16).

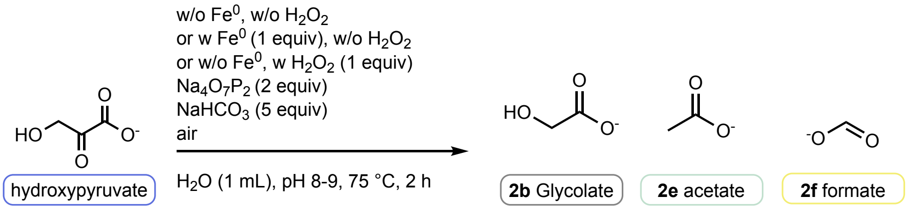

A. ^{1}H NMR of the reaction of hydroxypyruvate in our standard conditions

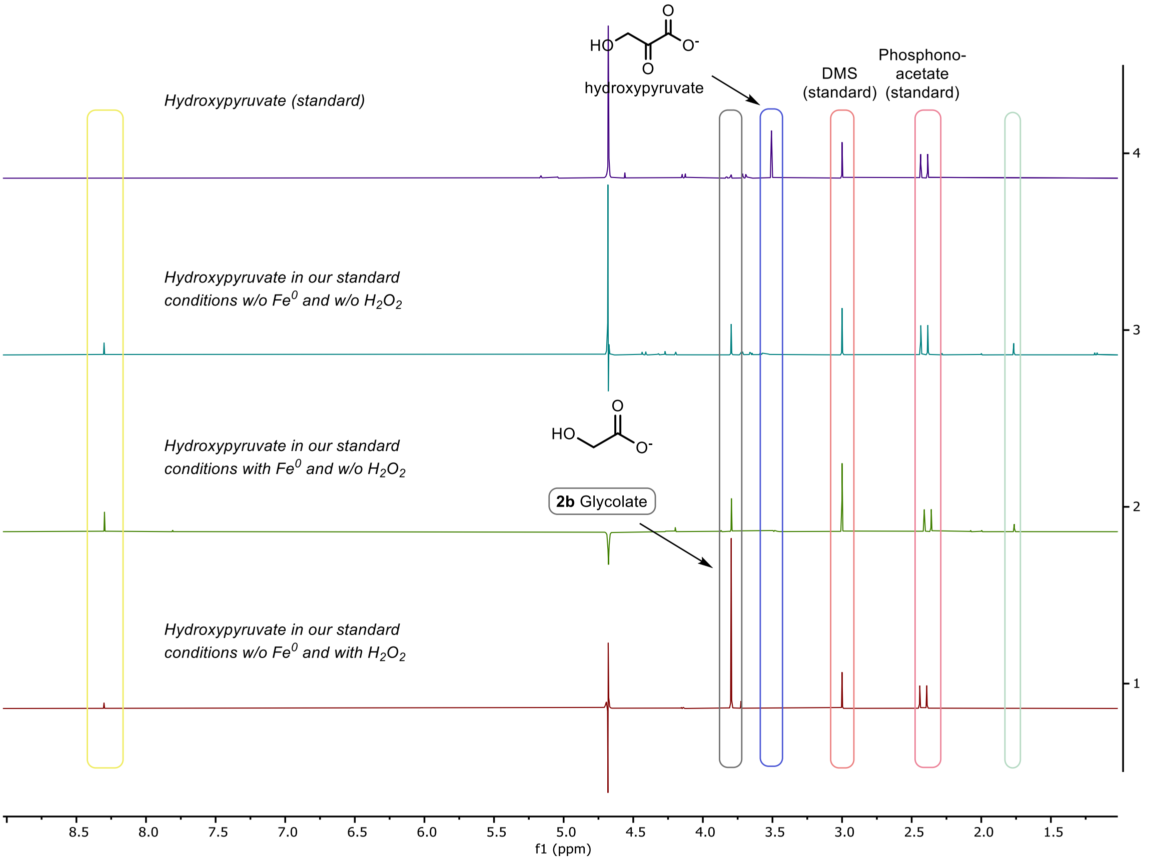

B. Proposed mechanism for the formation of glycolate (**2b**) from **PEP**

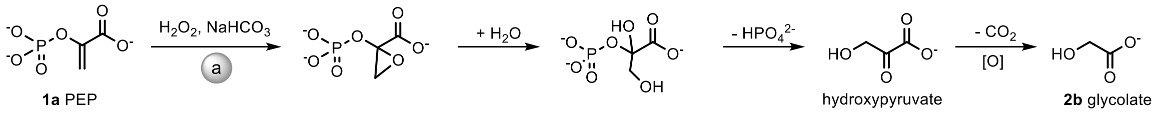

Figure S22. Study of hydroxypyruvate decomposition. (A) ^{1}H NMR study of hydroxypyruvate decomposition. Top: hydroxypyruvate in aqueous solution (pH 8-9) and, hydroxypyruvate in our standard conditions with or without Fe^0 and H_2O_2 after 2 h at 75 °C. DMS (internal standard) at 3 ppm (pink) and phosphonoacetate (internal standard) at approx. 2.5 ppm (doublet, pink). Hydroxypyruvate (blue) decomposes to glycolate (**2b**, grey) with or without Fe^0 or H_2O_2 after 2 h at 75 °C. (B) Proposed mechanism for the formation of glycolate (**2b**) from **PEP** in oxidative conditions. **2b** could have been formed through an alkene epoxidation[6] followed by the hydrolysis of the epoxide with subsequent dephosphorylation generating in-situ the intermediate hydroxypyruvate which decomposes (through oxidative decarboxylation) in these conditions into **2b**.

V. Investigating the hydration of PEP to 2PGA

A. Study of the promoter for the hydration reaction

We evaluated Fe(II)/Fe(III) species in different conditions (Table S8). An aqueous stock solution of **PEP** (48 mM), $Na_4O_7P_2$ (2 equiv), and $NaHCO_3$ (5 equiv) was freshly prepared for each reaction as follows: **PEP** (48 mM, 10 mg), $Na_4O_7P_2$ (2 equiv, 25 mg) and $NaHCO_3$ (5 equiv, 20 mg) were dissolved in 1 mL of MQ H_2O. After the addition of H_2O, gas bubbles were observed, and the mixture was vortexed until the dissolution of the remaining $Na_4O_7P_2$ powder. Then, Eppendorf vials were charged with the metal catalyst (1 equiv), a reducing agent Zn^0 (1 equiv, for entries 2, 5, and 11), and, 1 mL of the freshly prepared stock solution was added in each reaction (except for entries 7 and 8, see footnotes "d" and "e" of Table S8 for procedure) in addition to H_2O_2 (30% w/w; 1 equiv, see Table S8, entries 3 and 6). To perform the reaction under air without evaporation of the solution under heating, a needle was introduced into the lid of the Eppendorf vial (Fig. S10A). Samples were placed on a pre-tempered thermoshaker, and incubated for a reaction time of 16 h at 75 °C with a speed of 800 rpm. After the indicated time, the **GP** for NMR sample preparation in the presence of metal catalysts was applied. All NMR samples were analyzed by a standard water suppression sequence (noesygppr1d, 400 MHz, ns = 16), and samples containing **2-PGA** (see table below) were subsequently analyzed by ^1H qNMR (d1 = 30 s, 8 scans).

Table S8. Investigation of the active promoter (n.d. = not detected / n.c. = observed but not calculated).

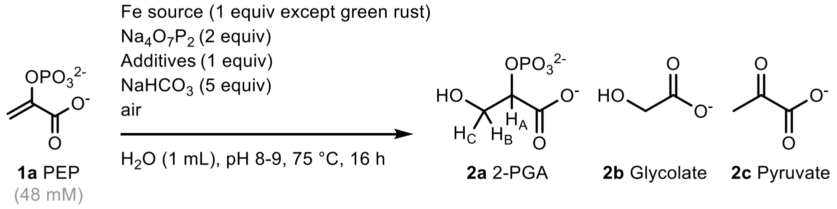

Entry	Metals	Additives	Int. **1a** (1 H, 5.21 ppm[b])	Yield **1a** (%)	Int. **2a** (1 H, H_B, 3.77-3.74 ppm)	Yield **2a** (%)	Int. **2b** (2 H, 3.8 ppm)	Yield **2b** (%)	Int. **2c** (3 H, 2.22 ppm)	Yield **2c** (%)	**PEP** (mM)	**DMS** (mM)[a]
1	$FeCl_3$	/	n.c.	n.c.	n.d.	n.d.	n.d.	n.d.	n.c.	n.c.	47.9	1.965
2[c]	$FeCl_3$	Zn^0	8.0	47.8	0.8	4.8	6.7	20.0	1.6	3.2	47.9	1.965
3[c]	$FeCl_3$	H_2O_2	n.c.	n.c.	n.d.	n.d.	n.c.	n.c.	n.c.	n.c.	47.9	1.963
4	$FeCl_2$	/	10.8	64.6	1.4	8.4	3.5	10.5	2.7	5.4	47.9	1.965
5	$FeCl_2$	Zn^0	8.4	50.2	1.9	11.4	4.3	12.9	2.1	4.2	47.9	1.965
6	$FeCl_2$	H_2O_2	6.2	37.1	1.3	7.8	6.6	19.7	1.9	3.8	47.9	1.965
7[d]	green Rust	/	5.5	32.9	0.6	3.6	3.3	9.9	14.0	27.9	47.8	1.963
8[e]	green Rust	/	10.2	70.1	1.4	9.6	1.7	5.8	0.4	0.9	47.8	1.804
9	α-FeOOH	/	n.c.	n.c.	n.d.	n.d.	n.d.	n.d.	n.c.	n.c.	47.9	1.963
10	Fe_3O_4	/	n.c.	n.c.	n.d.	n.d.	n.d.	n.d.	n.c.	n.c.	47.9	1.963
11	Fe^0	Zn^0	4.0	23.9	3.4	20.4	4.9	14.7	0.4	0.8	47.8	1.963

[a] The yields were determined relative to dimethyl sulfone used as an internal standard (integral set to 6 H, concentration inside the NMR tube reported in the last column).

[b] The proton of **PEP** at 5.21 ppm was chosen for quantification to minimize the loss of integration due to the ^1H water suppression method (for details, see Fig. S11).

[c] 0.5 equiv of $FeCl_3$ was used instead of 1 equiv.

[d] The green rust was prepared inside a glovebag following a procedure described in ref S7 [7] under a constant positive pressure of nitrogen using deoxygenated MQ H_2O. MQ H2O was degassed inside the glovebag by bubbling nitrogen vigorously for 3 h. A 100 µl solution containing 135 mM $FeSO_4.7H_2O$ and 67.5 mM $Fe_2(SO_4)_3$ was prepared using 100 µL of deoxygenated MQ H_2O. 100 µL of a deoxygenated 5 M NaOH solution was added dropwise forming a dark green precipitate and the final pH was approx. 7. Then, 800 µL of an aqueous stock solution of **PEP**, $Na_4O_7P_2$, and $NaHCO_3$ was transferred to the green rust mixture outside the glovebag, and the reaction was run for a reaction time of 16 h at 75 °C on a pre-tempered thermoshaker. After the indicated time, the **GP** for NMR sample preparation in the presence of metal catalysts was applied.

[e] The preparation of the green rust was adapted from the procedure described in ref S7 [7] outside the glovebag in the open air. A 100 µl solution containing 135 mM $FeSO_4·7H_2O$ and 67.5 mM $Fe_2(SO_4)_3$ was prepared using 100 µL of MQ H_2O. 100 µL of a 5 M NaOH solution was added dropwise forming a dark green precipitate and the final pH was approx. 7. Then, 800 µL of an aqueous stock solution of **PEP**, $Na_4O_7P_2$, and $NaHCO_3$ was transferred to the green rust mixture and the reaction was run for a reaction time of 16 h at 75 °C. After the indicated time, the **GP** for NMR sample preparation in the presence of metal catalysts was applied.

B. Control experiments to investigate the mechanistic requirements of the hydration reaction

1) Experiments under an inert atmosphere

We performed experiments in a glovebag under nitrogen with degassed solvents starting from different iron oxidation states (Table S9). $FeCl_2$ was opened, kept, and weighed in a glove box in a screw-cap vial. The vial was transferred into a glovebag under constant positive pressure of nitrogen. Other reagents or metal catalysts were weighed under air in vials flushed with nitrogen and introduced into the glovebag. Green rust was prepared inside the glovebag following a procedure described in ref S7[7]. MQ H_2O was degassed inside the glovebag by bubbling nitrogen vigorously for 3 h. The reaction was set up and run inside the glovebag in our standard conditions: **PEP** (48 mM), metal catalyst (1 equiv), $Na_4O_7P_2$ (2 equiv), and $NaHCO_3$ (5 equiv). Samples were placed on a pre-tempered thermoshaker inside the glovebag and incubated for a reaction time of 16 h at 75 °C with a speed of 800 rpm. After the indicated time, the **GP** for NMR sample preparation in the presence of metal catalysts was applied. All NMR samples were analyzed by a standard water suppression sequence (noesygppr1d, 400 MHz, ns = 16).

Table S9. Hydration reaction under an inert atmosphere analyzed by ^{1}H qNMR.

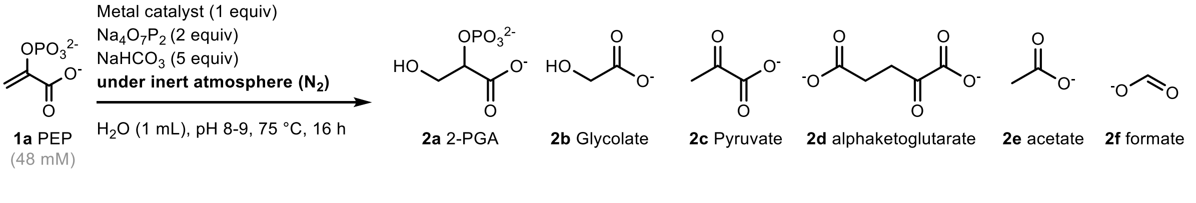

Entry	Metals	PEP	2-PGA	Glycolate	Pyruvate	α-ketoglutarate	Acetate	Formate
1	$FeCl_3$	Yes	No	No	Yes	No	Yes	No
2	$FeCl_2$	Yes	Traces	Traces	Yes	No	Yes	Traces
3	Fe^0	Yes	Traces	Yes	Yes	No	Yes	Traces
4[a]	Green Rust	Yes	No	No	Yes	No	Yes	Traces

[a] The green rust was prepared inside a glovebag following a procedure described in ref S7 [7] under a constant positive pressure of nitrogen using deoxygenated MQ H_2O. MQ H_2O was degassed inside the glovebag by bubbling nitrogen vigorously for 3 h. A 100 µl solution containing 135 mM $FeSO_4.7H_2O$ and 67.5 mM $Fe_2(SO_4)_3$ was prepared using 100 µL of deoxygenated MQ H_2O. 100 µL of a deoxygenated 5 M NaOH solution was added dropwise forming a dark green precipitate and the final pH was approx. 7. Then, 800 µL of an aqueous stock solution of **PEP**, $Na_4O_7P_2$, and $NaHCO_3$ was transferred to the green rust mixture, and the reaction was run for a reaction time of 16 h at 75 °C on a pre-tempered thermoshaker with a speed of 800 rpm. After the indicated time, the **GP** for NMR sample preparation in the presence of metal catalysts was applied.

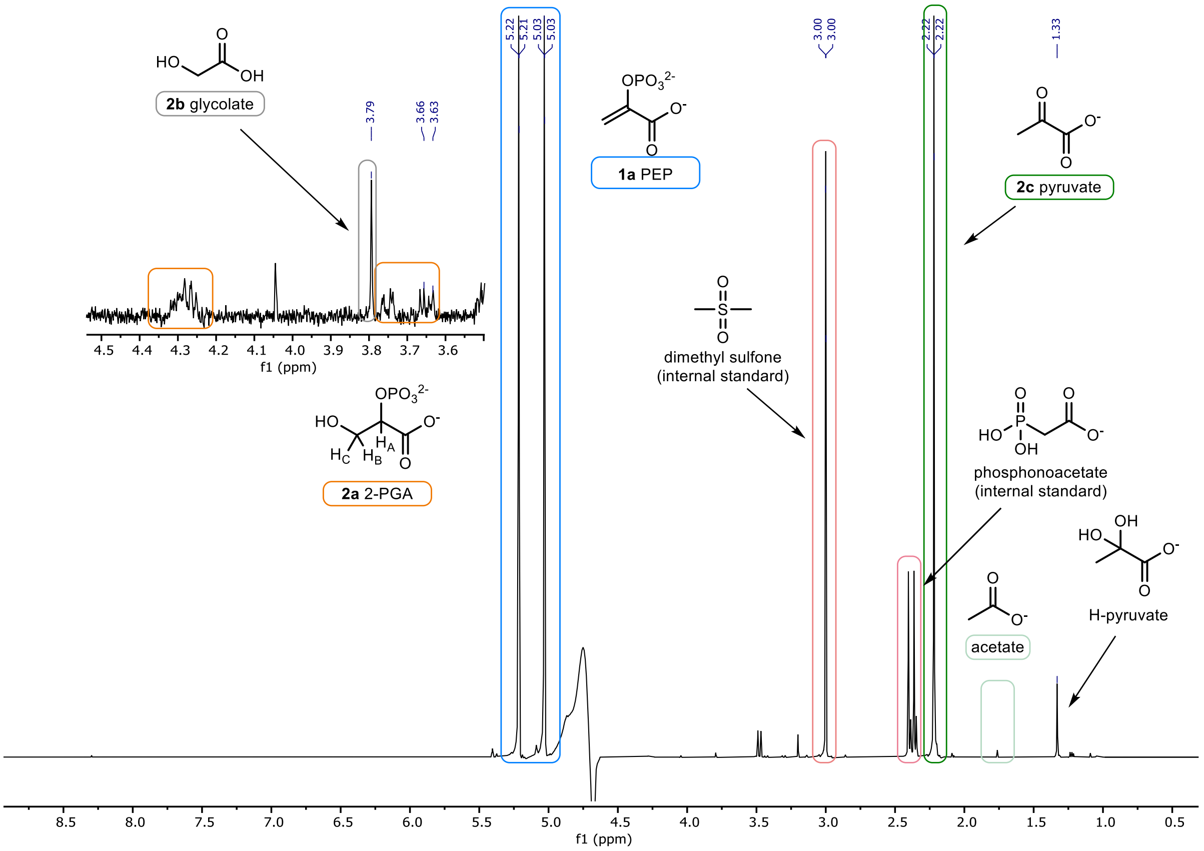

Figure S23. Typical ^1H NMR spectrum (400 MHz, ns = 16, d1 = 2 s) for the experiments performed under inert atmosphere (pH 13-14 due to the addition of the thiolate/phosphate solution to precipitate metals). In this case, the ^1H NMR spectrum corresponds to Table S9-entry 3.

2) Radical trapping experiments

We performed radical trapping experiments using 2,2,6,6-Tetramethylpiperidinyloxy (TEMPO), 2-propanol, or tert-butanol in our standard conditions in the presence of Fe^0 (Fig. S24). An aqueous stock solution of **PEP** (48 mM), $Na_4O_7P_2$ (2 equiv), and $NaHCO_3$ (5 equiv) was freshly prepared for each reaction as follows: **PEP** (48 mM, 10 mg), $Na_4O_7P_2$ (2 equiv, 25 mg) and $NaHCO_3$ (5 equiv, 20 mg) were dissolved in 1 mL of MQ H_2O. After the addition of H_2O, gas bubbles were observed, and the mixture was vortexed until the dissolution of the remaining $Na_4O_7P_2$ powder. Then, Eppendorf vials were charged with the metal catalyst (1 equiv), the radical trapping reagent (1 equiv), and 1 mL of the freshly prepared stock solution was added. To perform the reaction under air without evaporation of the solution under heating, a needle was introduced into the lid of the Eppendorf vial (Fig. S10A). Samples were placed on a pre-tempered thermoshaker, and incubated for a reaction time of 16 h at 75 °C with a speed of 800 rpm. After the indicated time, the **GP** for NMR sample preparation in the presence of metal catalysts was applied. All NMR samples were analyzed by ^1H qNMR (d1 = 30 s, 8 scans). The radical trapping experiment using TEMPO was subsequently analyzed by mass spectroscopy to detect any potential TEMPO adduct (Fig. S24B).

Table S10. Radical trapping experiments analyzed by ^1H qNMR[a]

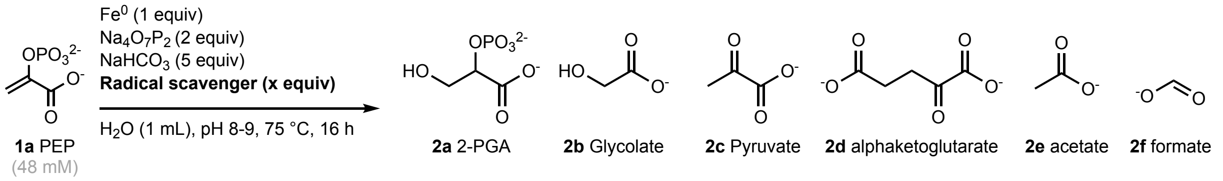

Entry	Reagent (x equiv)	Int. **1a** (1 H, 5.21 ppm[b])	Yield **1a** (%)	Int. **2a** (1 H, H_B, 3.77-3.74 ppm)	Yield **2a** (%)	Int. **2b** (2 H, 3.8 ppm)	Yield **2b** (%)	Int. **2c** (3 H, 2.22 ppm)	Yield **2c** (%)	**PEP** (mM)	**DMS** (mM)[a]
**1**[c]	TEMPO (1)	13.5	80.8	0.1	0.6	2.8	8.4	1.4	2.8	47.8	1.961
**2**[d]	TEMPO (1)	10.7	64.1	0.3	1.8	8.7	26.0	2.1	4.2	47.8	1.963
**3**	2-propanol (2)	10.1	60.5	2.5	15.0	2.1	6.3	2.3	4.6	47.8	1.963
**4**	tert-butanol (2)	11.6	69.4	1.3	7.8	8.0	23.9	0.9	1.8	47.8	1.963

[a] The yields were determined relative to dimethyl sulfone used as an internal standard (integral set to 6 H, concentration inside the NMR tube reported in the last column).

[b] The proton of **PEP** at 5.21 ppm was chosen for quantification to minimize the loss of integration due to the ^1H water suppression method (for details, see Fig. S11).

[c] The reaction was also run using $FeCl_2$ instead of Fe^0 showing a similar result without quantification.

[d] TEMPO (1 equiv) was added after 6 h of reaction.

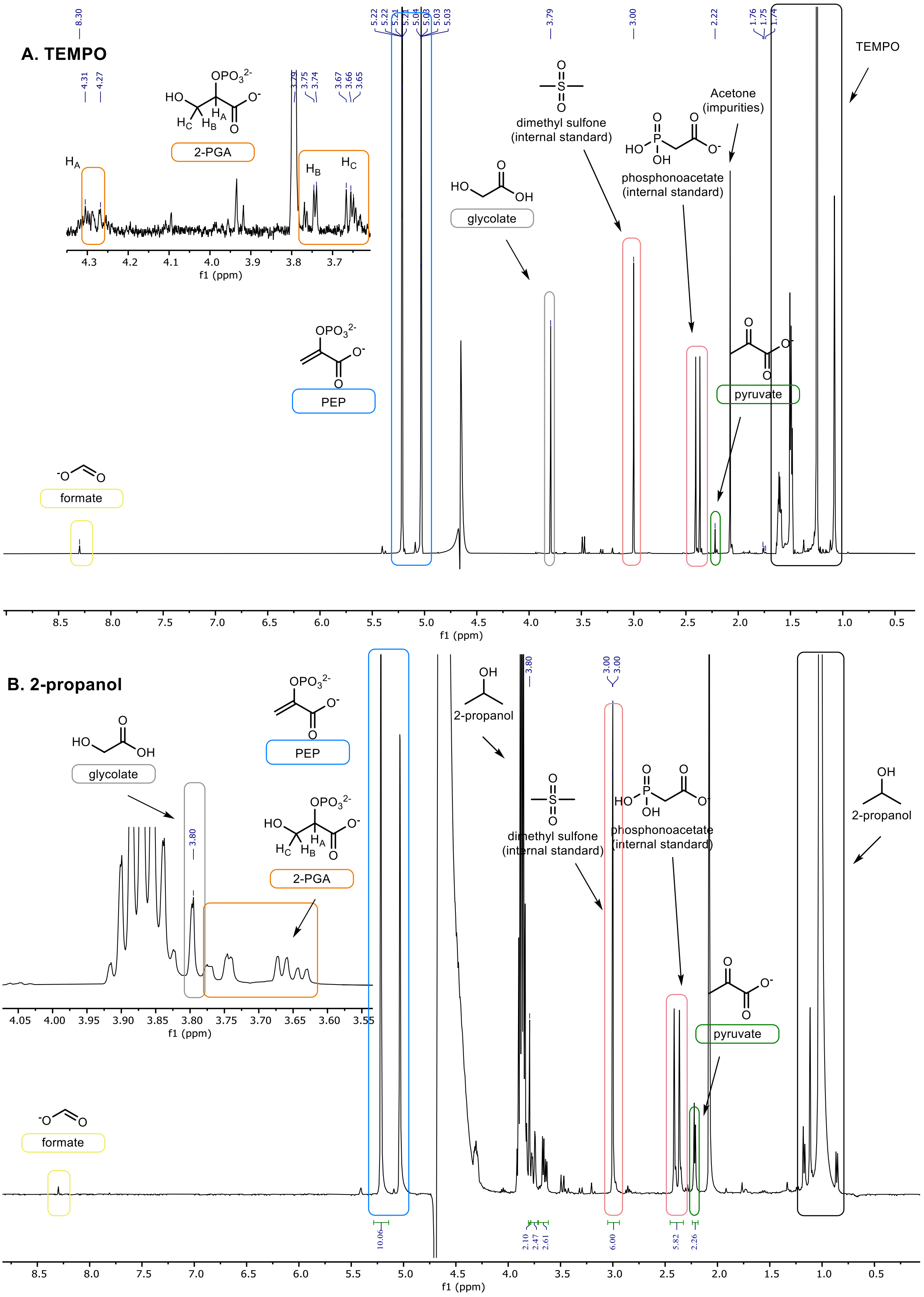
A. TEMPO
2-PGA
glycolate
PEP
formate
dimethyl sulfone
(internal standard)
phosphonoacetate
(internal standard)
Acetone
(impurities)
pyruvate
TEMPO
f1 (ppm)
B. 2-propanol
glycolate
PEP
2-PGA
formate
2-propanol
dimethyl sulfone
(internal standard)
phosphonoacetate
(internal standard)
pyruvate
2-propanol
f1 (ppm)

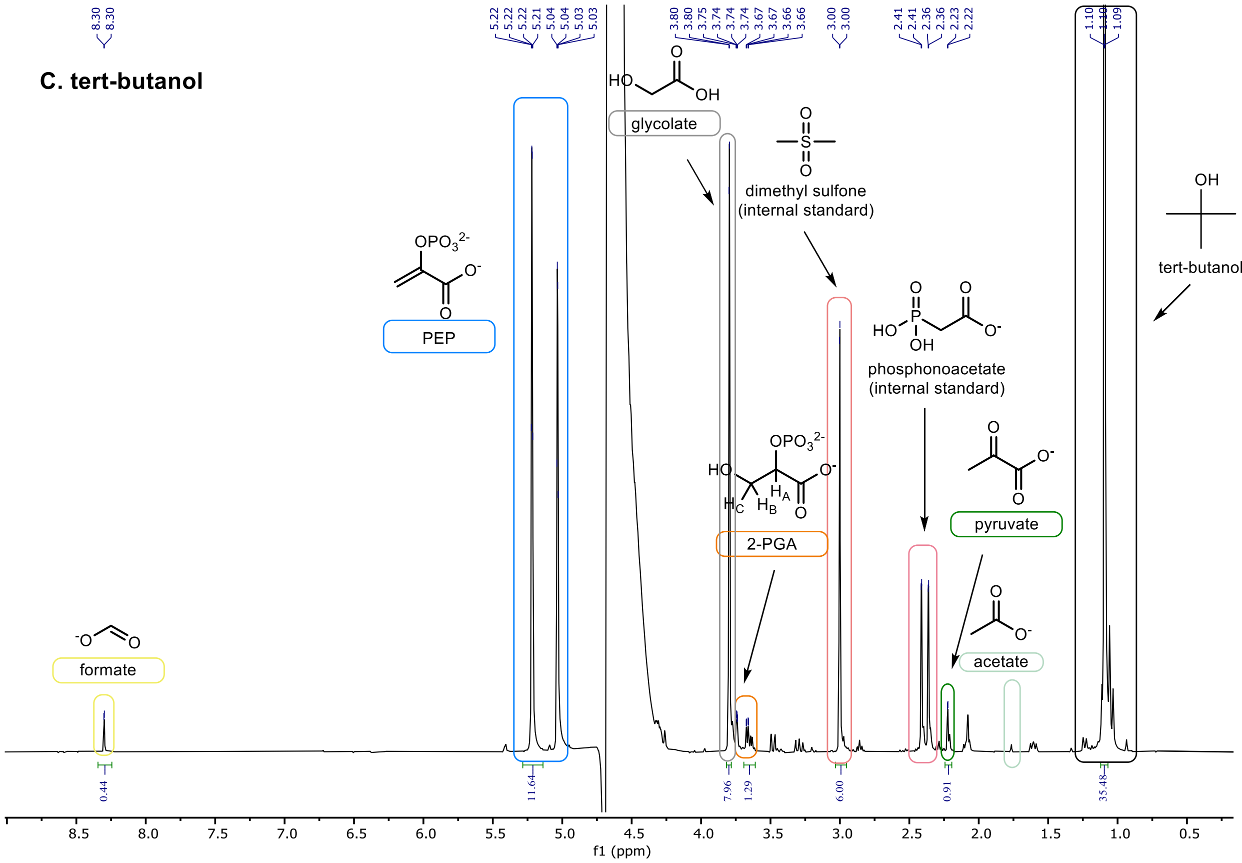

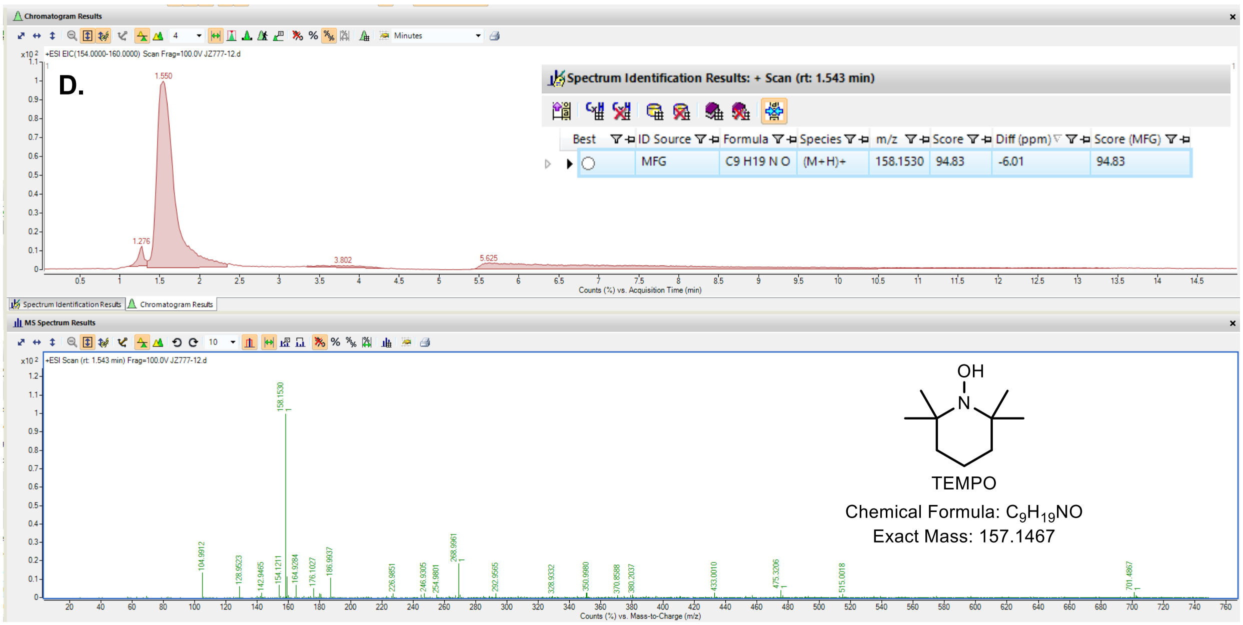

Figure S24. Radical trapping experiments. (A) ^{1}H NMR spectrum of the reaction mixture obtained when TEMPO was used. (B) ^{1}H NMR spectrum of the reaction mixture obtained when 2-propanol was used. (C) ^{1}H NMR spectrum of the reaction mixture obtained when tert-butanol was used. (D) QTOF analysis of the radical trapping experiment in which only the mass of TEMPO without any TEMPO adducts.

VI. Scope of the reaction

A. General procedure for the hydration of other biological substrates

An aqueous stock solution of the substrate (48 mM), $Na_4O_7P_2$ (2 equiv, 25.5 mg) and $NaHCO_3$ (5 equiv, 20 mg) was freshly prepared in 1 mL of MQ H_2O for each reaction. The mixture was vortexed until the dissolution of the remaining $Na_4O_7P_2$ powder. Then, Eppendorf vials were charged with Fe^0 (1 equiv, 2.7 mg), and 1 mL of the freshly prepared stock solution was added. To perform the reaction under air without evaporation of the solution under heating, a needle was introduced into the lid of the Eppendorf vial (see Fig. S10A). Samples were placed on a pre-tempered thermoshaker, and incubated for different reaction times at different temperatures with a speed of 800 rpm. After the indicated time, the **GP** for NMR sample preparation in the presence of metal catalysts was applied. The products were quantified by quantitative ^{1}H NMR (d1 = 30 s, 8 scans), using 500 µL of the reaction mixture and, if not noted otherwise, 50 µL of a 23.6 mM **DMS** stock solution in D_2O (total volume of NMR sample: 550 µL).

B. Hydration of fumarate (1b) to malate (3a)

Samples were prepared according to the general procedure **VI.A**.

Table S11. Hydration of fumarate to malate analyzed by ^{1}H qNMR[a].

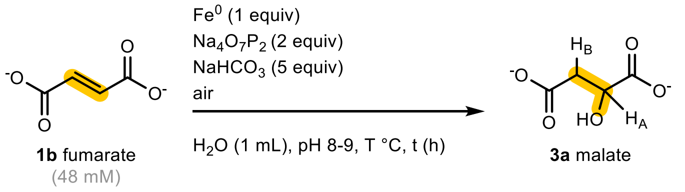

Entry	Temperature (T°, in °C)	Time (t, in hours)	Int. **1b** (2 H, 6.36 ppm)	Yield **1b** (%)	Int. **3a** (1 H, H_B, 2.49-2.54 ppm)	Yield **3a** (%)	**1b** (mM)	**DMS** (mM)[a]
**1**	25	144	11.8	35.4	1.2	7.2	47.8	2.144
**2**	40	16	24.5	72.1	3.4	20.0	47.8	2.144
**3**[b]	75	16	24.8	74.2	3.2	19.1	47.8	1.961

[a] The yields were determined using 500 µL of the reaction mixture and, if not noted otherwise, 50 µL of a 23.6 mM DMS stock solution in D_2O (total volume of NMR sample: 550 µL). Yields were calculated relative to dimethyl sulfone used as an internal standard (integral set to 6 H, concentration inside the NMR tube reported in the last column).
[b] The products were quantified by quantitative ^{1}H NMR (d1 = 30 s, 8 scans), using 500 µL of the reaction mixture and, 50 µL of a 23.6 mM DMS stock solution in D_2O, and 50 µL of a 77.8 mM phosphonoacetate stock solution in D_2O (total volume of NMR sample: 600 µL). Integral of **DMS** set to 6 H, concentration inside the NMR tube reported in the last column.

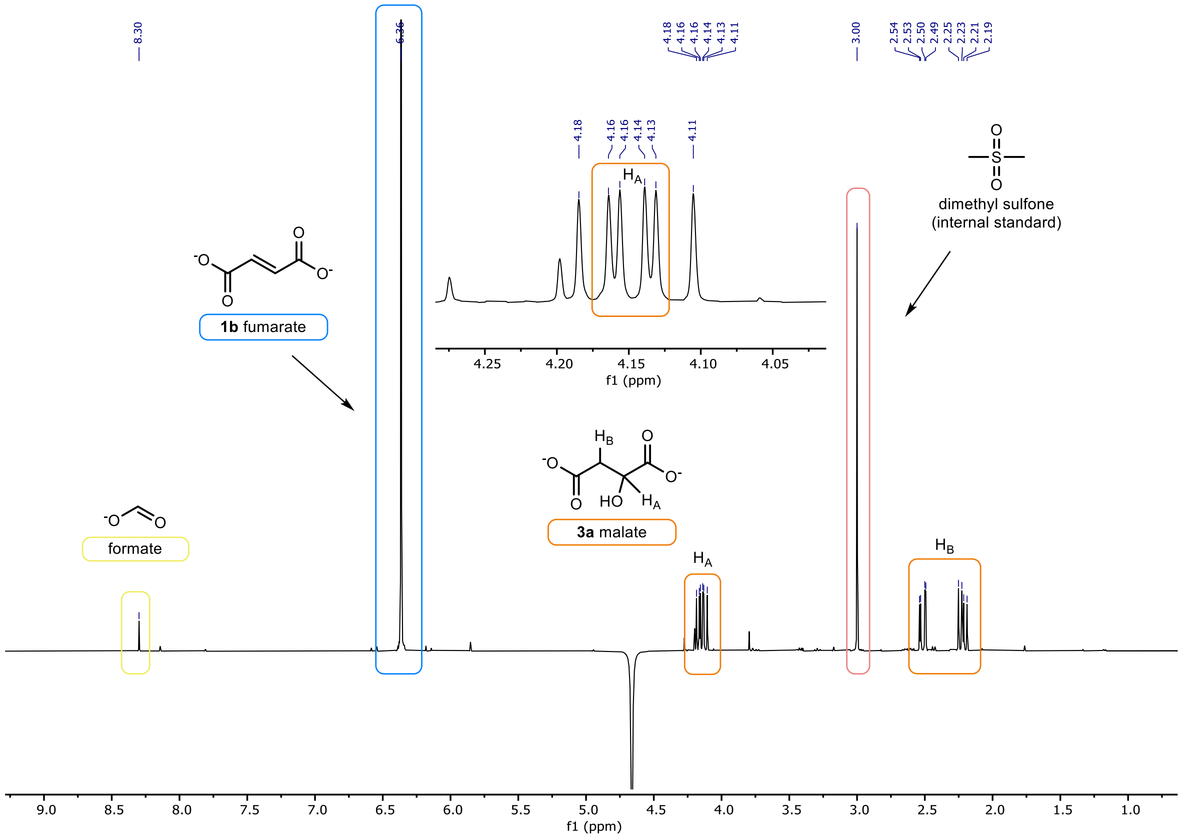

Figure S25. Typical ^{1}H NMR spectrum (400 MHz, ns = 8, d1 = 30 s) for the experiments where malate **3a** was observed (pH 13-14 due to the addition of the thiolate/phosphate solution to precipitate metals). In this case, the ^{1}H NMR spectrum corresponds to Table S10-entry 2.

C. Hydration of cis-aconitate (1c) to citrate (4a) and isocitrate (4b + 4b')

1) Study of the hydration of cis-aconitate (1c) over time

The hydration of aconitate can occur on two possible sites for the O-H insertion. We studied the mechanism by following the reaction over time. To do so, a series of identical reactions were quenched at different times and examined by ^{1}H NMR. An aqueous stock solution of cis-aconitic acid (50.4 mg, 48 mM), $Na_4O_7P_2$ (2 equiv, 153 mg) and $NaHCO_3$ (5 equiv, 121 mg) was freshly prepared in 6 mL of MQ H_2O. The mixture was vortexed until dissolution of the remaining $Na_4O_7P_2$ powder. Then, Eppendorf vials were charged with Fe^0 (1 equiv), and 1 mL of the freshly prepared stock solution was added. To perform the reaction under air without evaporation of the solution under heating, a needle was introduced into the lid of the Eppendorf vial (see Fig. S10A). Samples were placed on a pre-tempered thermoshaker and incubated at 75 °C for different reaction times (Fig. S26A) with a speed of 800 rpm. After the indicated time, the **GP** for NMR sample preparation in the presence of metal catalysts was applied. All NMR samples were analyzed by a standard water suppression sequence (noesygppr1d, 400 MHz, ns = 16).

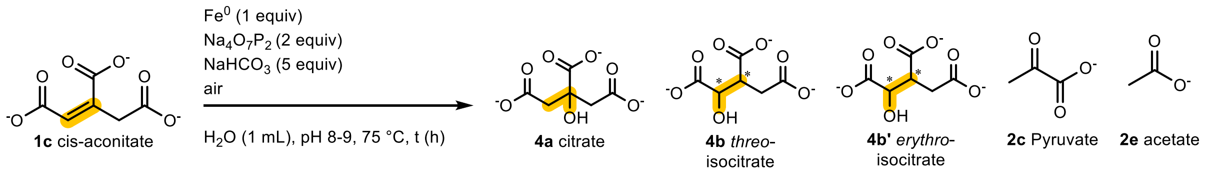

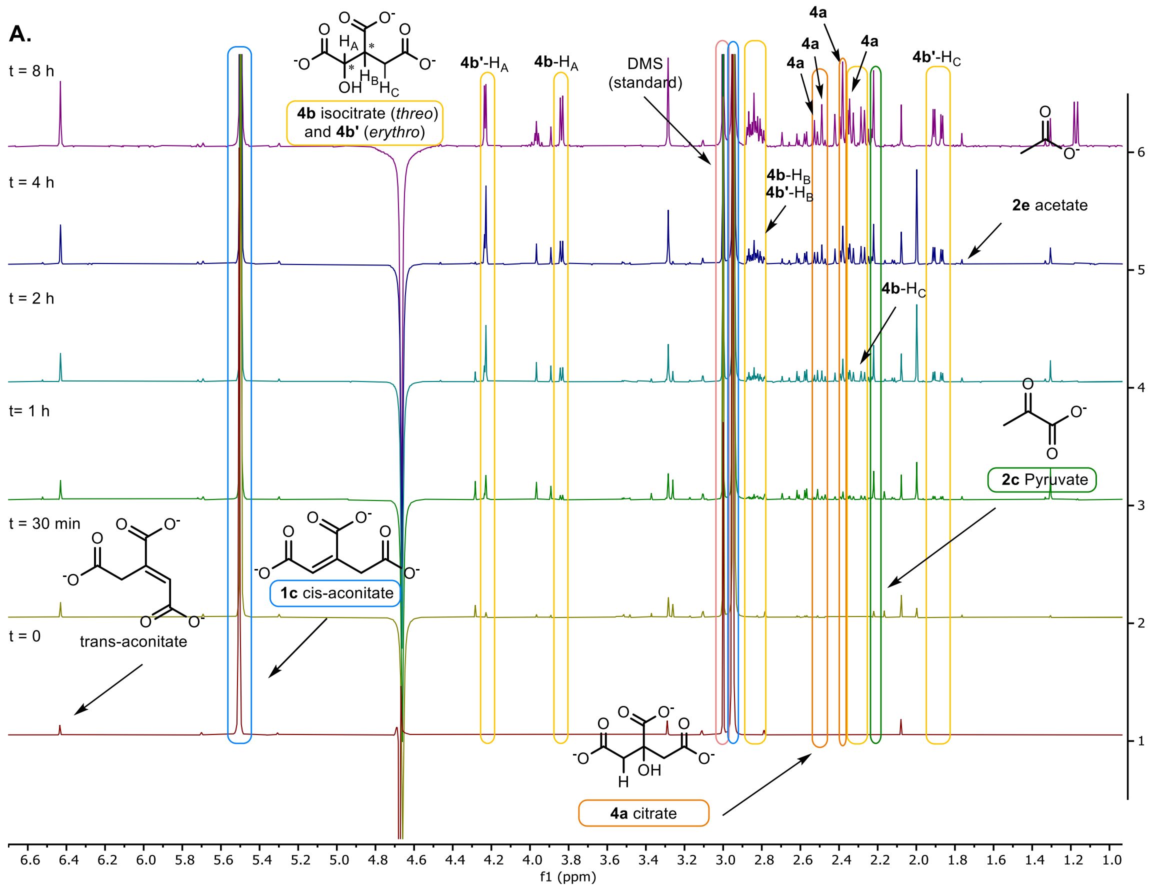

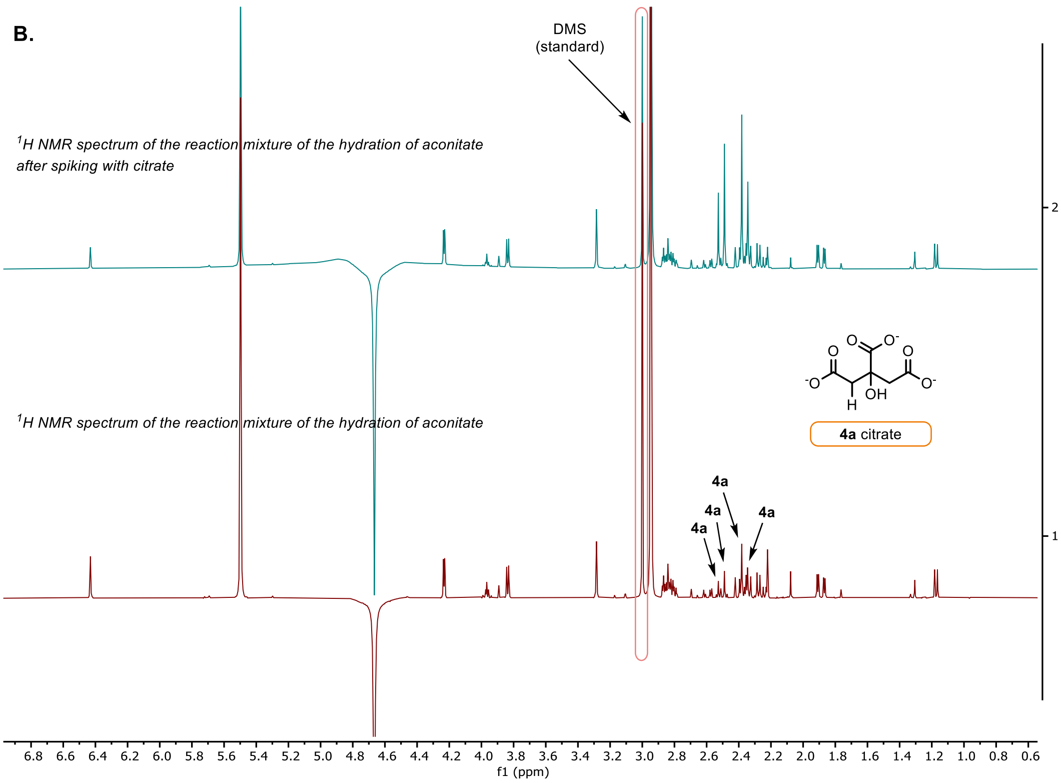

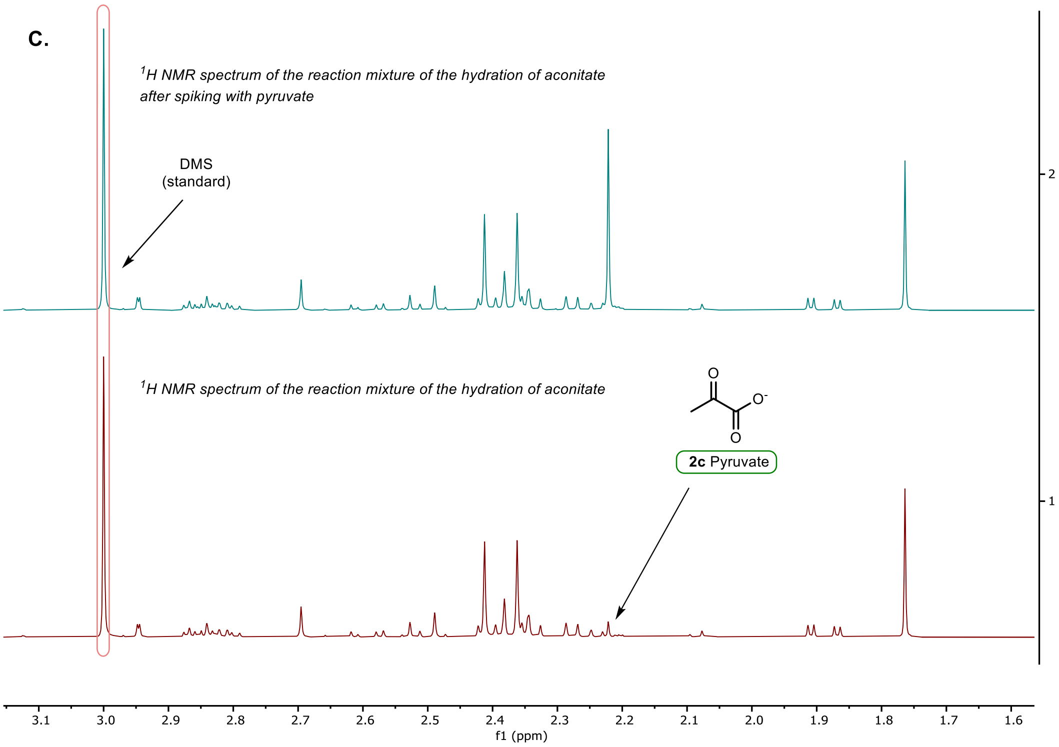

Figure S26. Study of the hydration of aconitate (**1c**). (A) Stacked ^{1}H NMR spectra showing progress of the hydration of **1c** to **4a** and a mixture of diastereoisomers of isocitrate **4b** (**4b**: *threo*-isocitrate, **4b'**: *erythro*-isocitrate). The assignment of peaks related to **4a** and **4b** were verified against the commercially available products (for details, see Fig. S8-S9) and previously reported assignments.[1] Both hydrated products are formed at the same time over the investigated time period (pH 13-14 due to the addition of the thiolate/phosphate solution to precipitate metals). (B) ^{1}H NMR of the reaction mixture (after a reaction time of 8 h) spiked with commercially available citrate **4a** (pH 13-14 due to the addition of the thiolate/phosphate solution to precipitate metals). (C) ^{1}H NMR of the reaction mixture (after a reaction time of 16 h) spiked with commercially available pyruvate **2c** (pH 13-14 due to the addition of the thiolate/phosphate solution to precipitate metals).

2) Hydration of cis-aconitate (1c)

Samples were prepared according to the general procedure **VI.A**.

Table S12. Hydration of aconitate analyzed by ^{1}H qNMR[a] (n.c. = observed but not calculated).

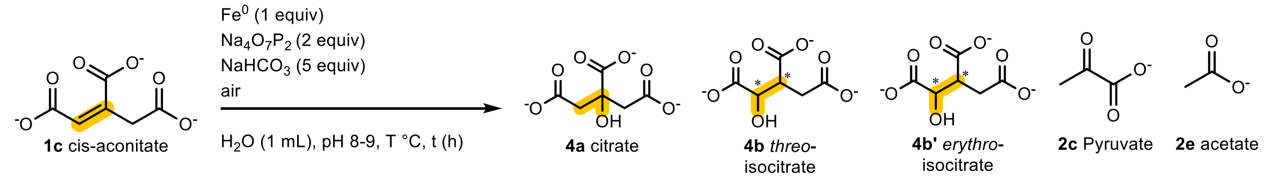

Entry	T° (°C)	Time (hours)	Int. **1c** (1 H, 5.5 ppm)	Yield **1c** (%)	[b]Int. **4a** (2 H, 2.53 and 2.49 ppm)	Yield **4a** (%)	Int. **4b** (1 H_A, 3.84 ppm)	Yield **4b** (%)	Int. **4b'** (1 H_A, 4.23 ppm)	Yield **4b'** (%)	Int. **2c** (3 H, 2.22 ppm)	Yield **2c** (%)	**1c** (mM)	**DMS** (mM)[a]
**1**	25	144	7.7	45.2	[c]n.c	[c]n.c	0.8	4.7	1.3	7.6	1.5	2.9	48.8	2.144
**2**	40	16	11.5	68.3	0.9	2.7	1.2	7.1	2.0	11.9	1.4	2.8	48.2	2.144
**3**[d]	75	16	5.7	30.5	1.2	3.6	1.1	6.7	1.4	8.5	0.7	1.3	47.1	1.961

[a] The yields were determined using 500 µL of the reaction mixture and, if not noted otherwise, 50 µL of a 23.6 mM DMS stock solution in D_2O (total volume of NMR sample: 550 µL). Yields were calculated relative to dimethyl sulfone used as an internal standard (integral set to 6 H, concentration inside the NMR tube reported in the last column).

[b] Quantification of citrate (**4a**) by integration of one doublet (2 H) of the doublet of doublet (2 x CH_2, 4 H) which corresponds to the two peaks at 2.53 ppm and 2.49 ppm, the other signal of the second proton being overlapped with other signals.

[c] Quantification of citrate (**4a**) was not possible in this case because of the low intensity of its characteristic peaks and a possible overlapping with other peaks.

[d] The products were quantified by quantitative ^{1}H NMR (d1 = 30 s, 8 scans), using 500 µL of the reaction mixture and, 50 µL of a 23.6 mM DMS stock solution in D_2O, and 50 µL of a 77.8 mM phosphonoacetate stock solution in D_2O (total volume of NMR sample: 600 µL). Integral set to 6 H, concentration inside the NMR tube reported in the last column).

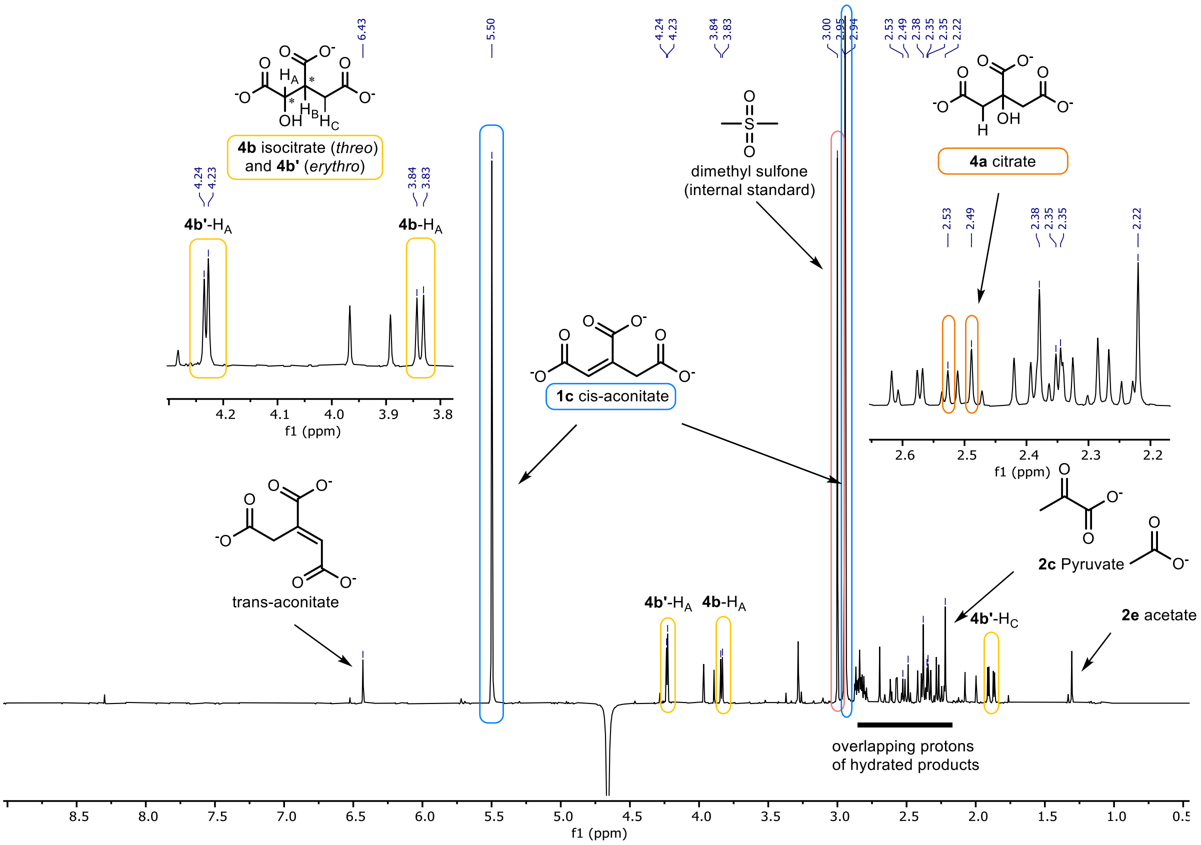

Figure S27. Typical ^{1}H NM spectrum (400 MHz, ns = 8, d1 = 30 s) for the experiments where citrate (**4a**) and isocitrate diastereoisomers (**4b** and **4b'**), pyruvate (**2c**) and acetate (**2e**) were observed with zooms on the peaks which were used for quantification of each species (pH 13-14 due to the addition of the thiolate/phosphate solution to precipitate metals). In this case, the ^{1}H NMR spectrum corresponds to Table S11-entry 2 with a zoom on the 3.8-4.3 ppm region.

3) Study of the retro-aldol reaction of citrate (4a)

The formation of pyruvate (**2c**) being observed in the reaction mixture after aconitate (**1c**) was subjected to our reaction conditions, we investigated whether the formation of **2c** could occur through the retro-aldol reaction of citrate into acetate and oxaloacetate with subsequent decarboxylation of the latter to **2c**. Therefore, **4a** and **4b** (Table S13-entry 6), the hydration products of **1c**, were subjected to our standard conditions (Table S13, Fig. S28A-B). Moreover, in the case of **4a**, the reaction was also run the presence of Zn^0 (5 equiv) to trap oxaloacetate via its reduction to malate (**3a**, Table S13 - entries 2 and 4, Fig. S28C).

Samples were prepared according to the general procedure **VI.A**. For entries 2 and 4, Zn^0 (5 equiv) was added to the reaction mixture.

Table S13. Retro-aldol reaction of citrate and isocitrate[b] (n.d. = not detected / n.c. = observed but not calculated).

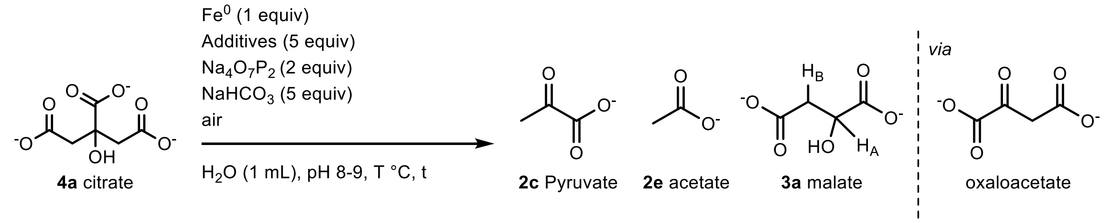

Entry	T° (°C)	Time (hours)	Additives	Int. **4a** (2 H, 2.53 and 2.49 ppm)	Yield **4a** (%)	Int. **2c** (3 H, 2.22 ppm)	Yield **2c** (%)	Int. **2e** (3 H, 1.76 ppm)	Yield **2e** (%)	Int. **3a** (1 H, H_A, 4.14-4.16 ppm)	Yield **3a** (%)	**4a** (mM)	**DMS** (mM)[a]
**1**	25	72	/	25.7	77.2	0.6	1.2	0.1	0.2	0.1	0.6	47.6	2.142
**2**	25	72	Zn	26.3	79.0	1.0	2.0	0.1	0.2	0.3	1.8	47.6	2.142
**3**	40	16	/	25.5	76.6	0.8	1.6	0.1	0.2	traces	traces	47.6	2.142
**4**	40	16	Zn	26.7	80.2	2.3	4.6	0.1	0.2	0.2	1.2	47.6	2.142
**5**	75	16	/	n.c.	n.c.	n.c.	n.c.	n.c.	n.c.	n.d.	n.d.	47.6	2.142
**6**[b]	75	16	/	n.c.	n.c.	n.d.	n.d.	n.d.	n.d.	n.d.	n.d.	47.6	2.142

[a] The yields were determined using 500 µL of the reaction mixture and, if not noted otherwise, 50 µL of a 23.6 mM DMS stock solution in D_2O (total volume of NMR sample: 550 µL). Yields were calculated relative to dimethyl sulfone used as an internal standard (integral set to 6 H, concentration inside the NMR tube reported in the last column).

[b] The reaction was run from isocitrate (**4b**) as a control experiment.

A.

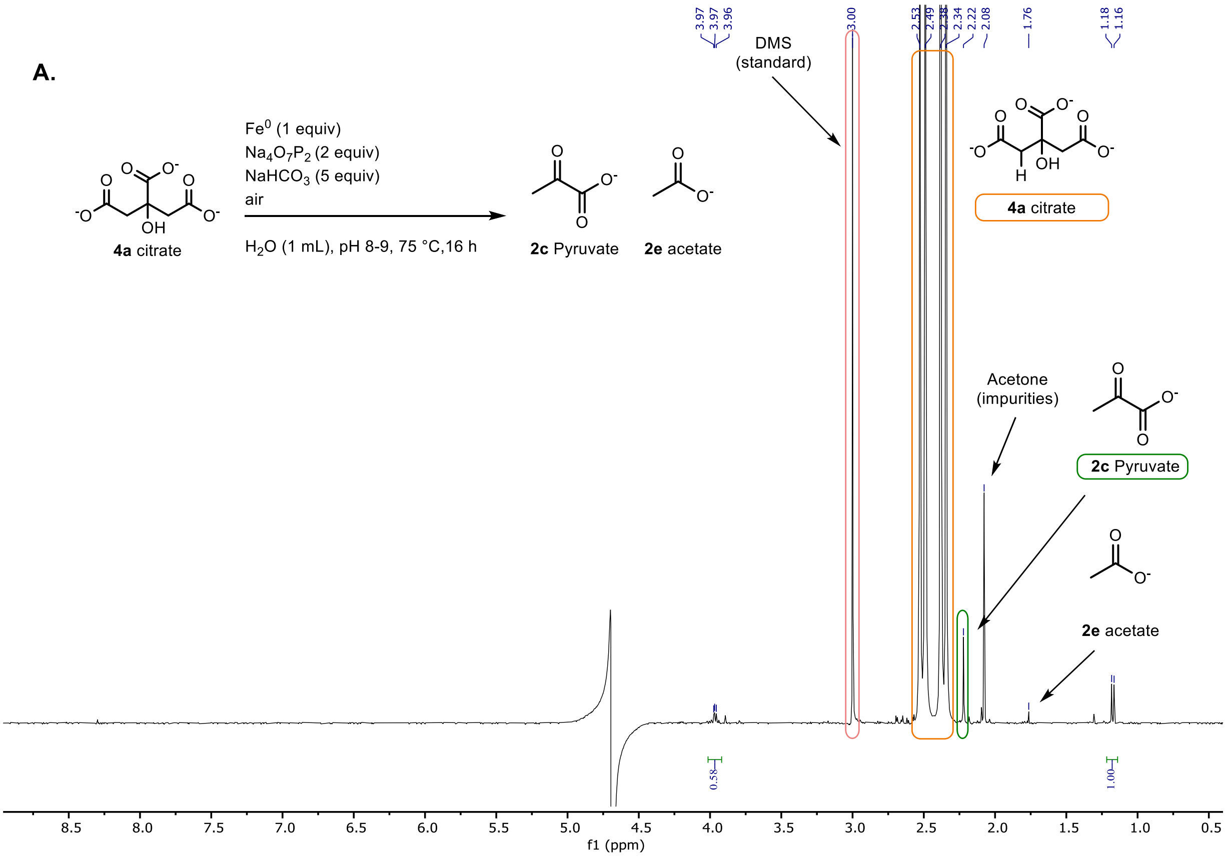

4a citrate
Fe0 (1 equiv)
Na4O7P2 (2 equiv)
NaHCO3 (5 equiv)
air
H2O (1 mL), pH 8-9, 75 °C,16 h
2c Pyruvate
2e acetate
DMS
(standard)
Acetone
(impurities)
f1 (ppm)

B.

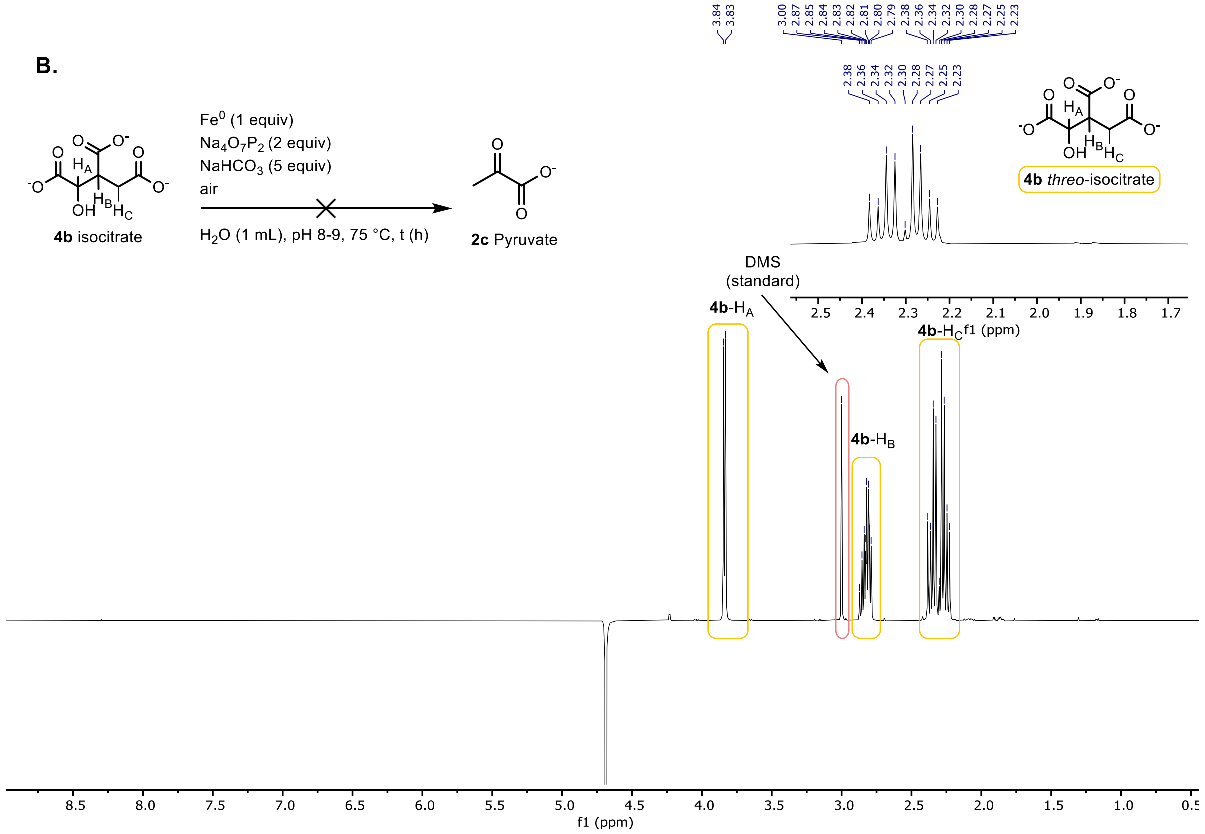

4b isocitrate
Fe0 (1 equiv)
Na4O7P2 (2 equiv)
NaHCO3 (5 equiv)
air
H2O (1 mL), pH 8-9, 75 °C, t (h)
2c Pyruvate
4b threo-isocitrate
DMS
(standard)
4b-HA
4b-HB
4b-HC
f1 (ppm)

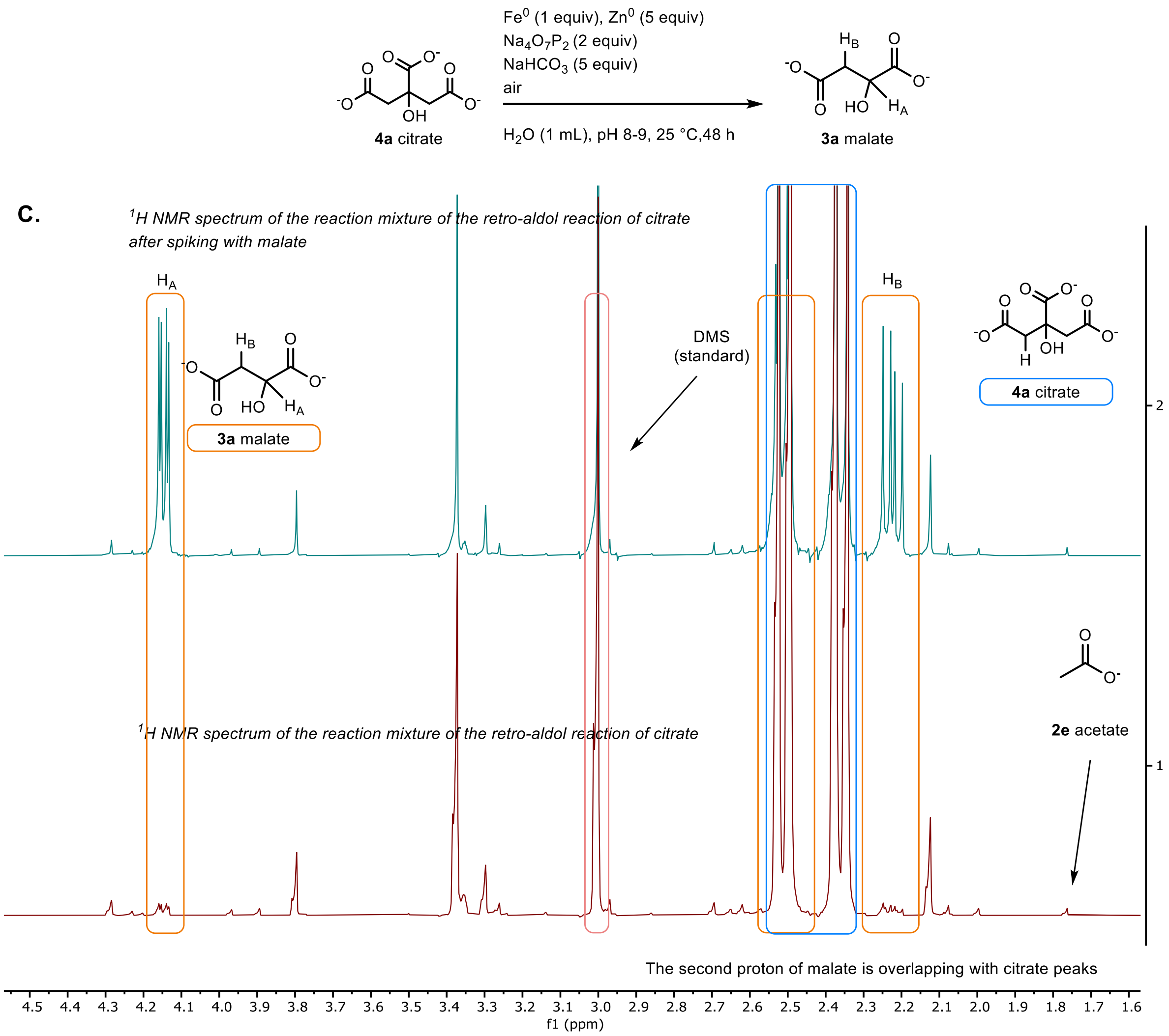

Figure S28. (A) ^{1}H NMR spectrum (noesygppr1d, 400 MHz, ns = 16) of the reaction mixture obtained when citrate (**4a**) was subjected to our standard conditions in which pyruvate (**2c**) and acetate (**2e**) were observed (pH 13-14 due to the addition of the thiolate/phosphate solution to precipitate metals). Example of Table S13-Entry 5. (B) ^{1}H NM spectrum (noesygppr1d, 400 MHz, ns = 16) of the reaction mixture obtained when isocitrate (**4b**) was subjected to our standard conditions. In this case, we did not observe the formation of pyruvate (**2c**). Example of Table S13-Entry 6. (C) ^{1}H NMR of the reaction mixture (after a reaction time of 72 h at 25 °C, Table S13-Entry 2) spiked with commercially available malate **3a** (pH 13-14 due to the addition of the thiolate/phosphate solution to precipitate metals).

D. Hydration of itaconate (1d) to (5a)

Samples were prepared according to the general procedure **VI.A**. Eppendorf vials were charged with Fe^0 (1 equiv, 2.7 mg) or H_2O_2 (30% w/w; 1 equiv, see Table S14). Samples were subsequently analyzed by mass spectroscopy to further confirm the formation of the products (**5a** and **5b**, Fig. S29C-D).

Table S14. Hydration of itaconate analyzed by ^{1}H qNMR[a] (n.d. = not detected / n.c. = observed but not calculated).

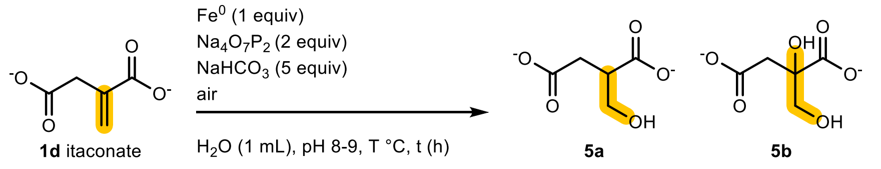

Entry	Fe^0/H_2O_2	T° (°C)	Time (t, in hours)	Int. **1d** (1 H, 5.85 ppm)	Yield **1d** (%)	Int. **5a**[8] (1 H, 2.22-2.28 ppm)	Yield **5a** (%)	Int. **5b** (1 H, 3.57-3.6 ppm)	Yield **5b** (%)	**1d** (mM)	**DSS** (mM)[a]
**1**	Fe^0	40	16	12.0	59.3	4.1	20.3	3.0	14.8	50.0	1.849
**2**[b]	Fe^0	75	16	13.9	60.3	4.5	20.5	0.9	4.1	47.7	1.849
**3**[c]	H_2O_2	75	16	n.c.	n.c.	n.d.	n.d.	n.c.	n.c.	47.8	1.849

[a] The yields were determined relative to sodium trimethylsilylpropanesulfonate (**DSS**) used as an internal standard instead of **DMS** because the **DMS** peak was overlapping with the $-CH_2$ group of itaconate. NMR sample preparation: 500 µL of the supernatant was transferred to an NMR tube, and 50 µL of a 20.3 mM stock solution of DSS (DSS in D_2O). Integral set to 9 H at 0 ppm and concentration inside the NMR tube reported in the last column.

[b] To precipitate metals, 150 µL of the thiolate/phosphate solution was added to 500 µL instead of 700 µL of the reaction mixture. The products were quantified by quantitative ^{1}H NMR (d1 = 30 s, 8 scans), using 400 µL of the reaction mixture and, 50 µL of a 20.3 mM stock solution of DSS (DSS in D_2O).

[c] When the reaction was run in the presence of H_2O_2 (1 equiv) instead of Fe^0, the formation of **5b** was detected by ^{1}H NMR (see Fig. S29B).

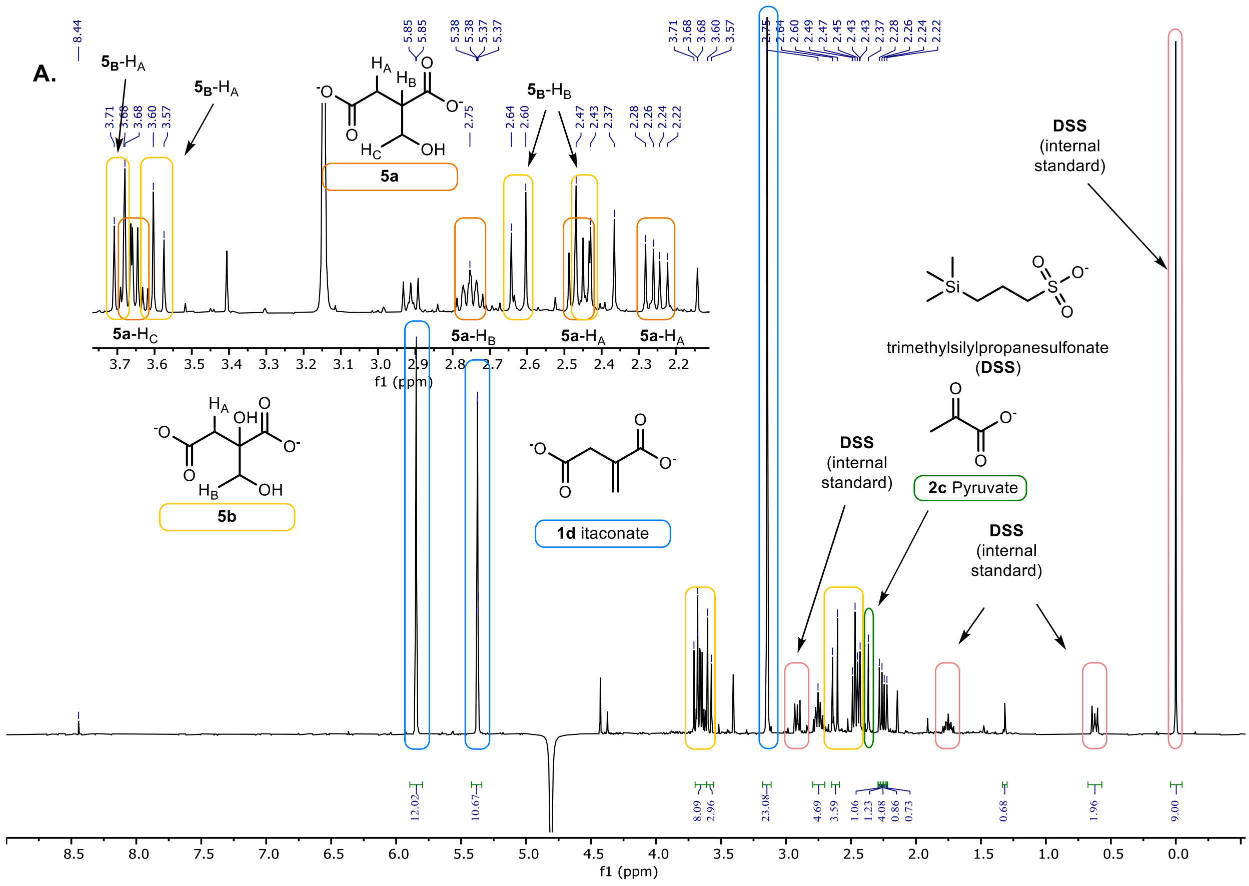

B.

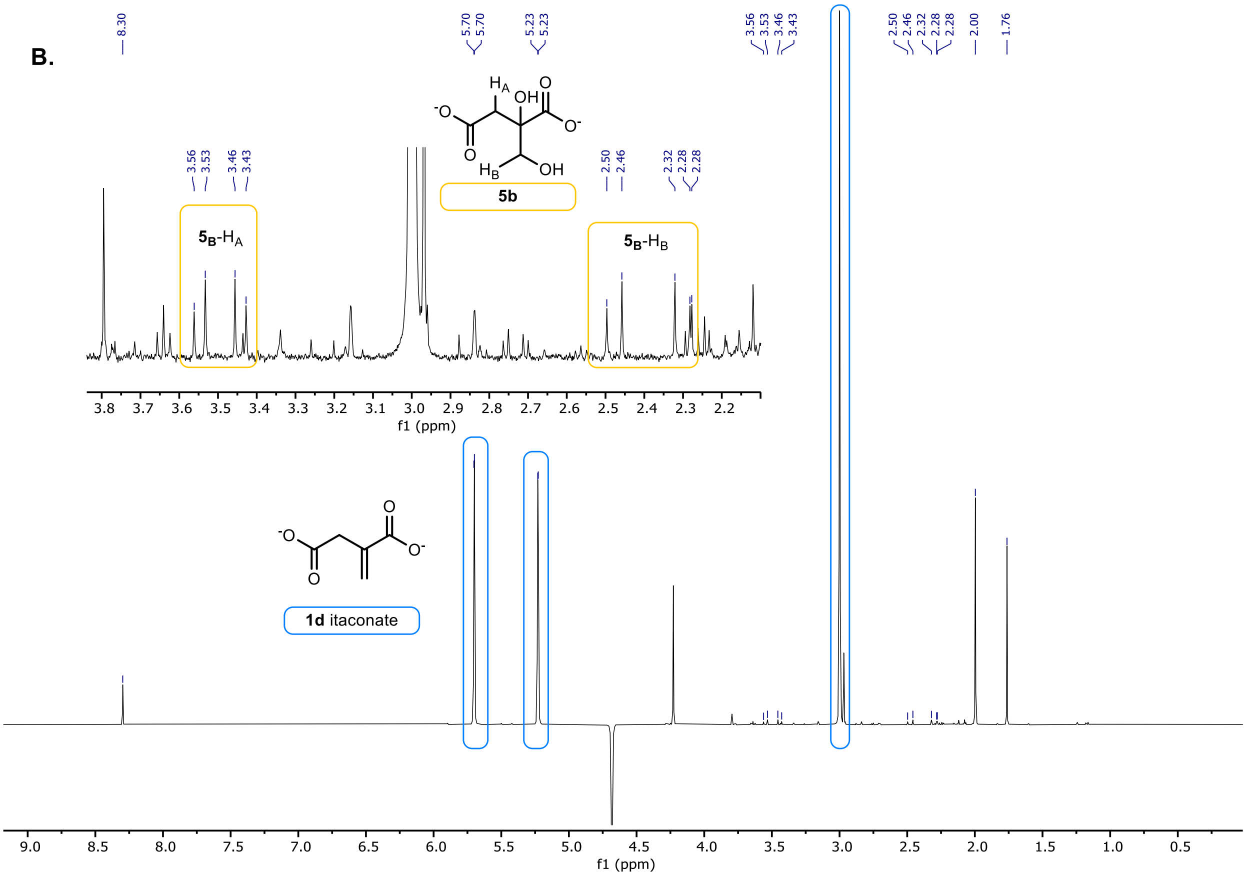

8.30
5.70
5.70
5.23
5.23
3.56
3.53
3.46
3.43
2.50
2.46
2.32
2.28
2.28
2.00
1.76
5b
5B-HA
5B-HB
1d itaconate
f1 (ppm)

C.

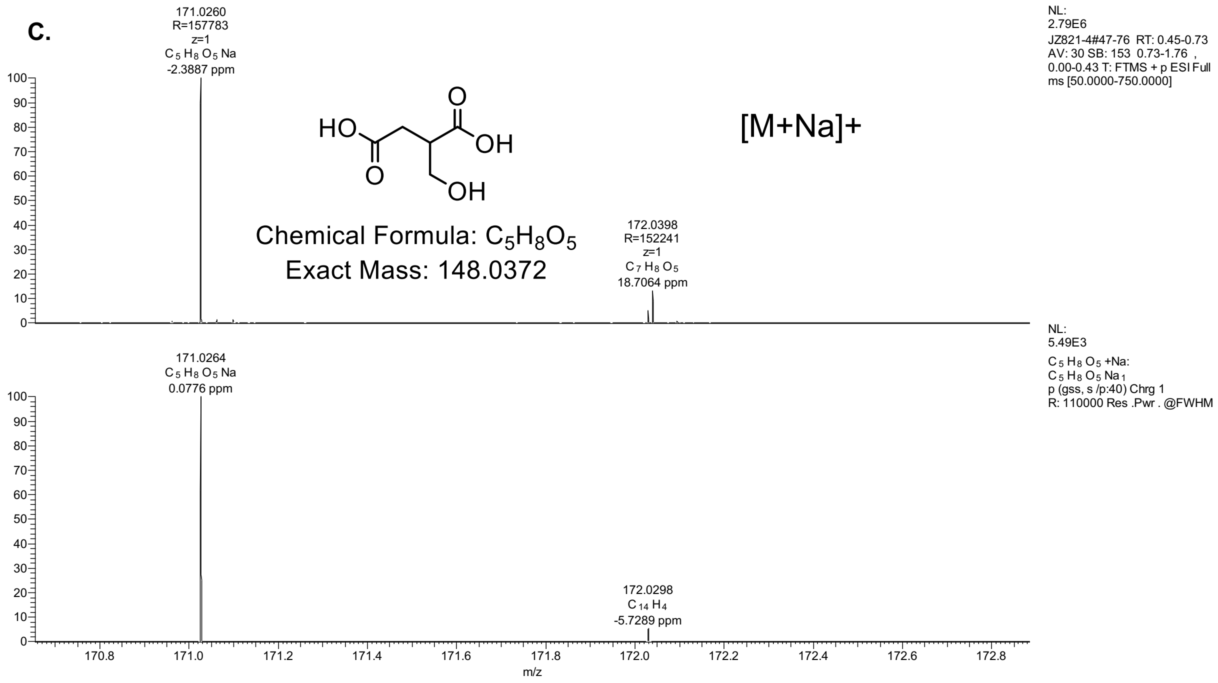

171.0260
R=157783
z=1
C5 H8 O5 Na
-2.3887 ppm
NL:
2.79E6
JZ821-4#47-76 RT: 0.45-0.73
AV: 30 SB: 153 0.73-1.76 ,
0.00-0.43 T: FTMS + p ESI Full
ms [50.0000-750.0000]
[M+Na]+
Chemical Formula: C5H8O5
Exact Mass: 148.0372
172.0398
R=152241
z=1
C7 H8 O5
18.7064 ppm
NL:
5.49E3
C5 H8 O5 +Na:
C5 H8 O5 Na1
p (gss, s /p:40) Chrg 1
R: 110000 Res .Pwr . @FWHM
171.0264
C5 H8 O5 Na
0.0776 ppm
172.0298
C14 H4
-5.7289 ppm
m/z

D.

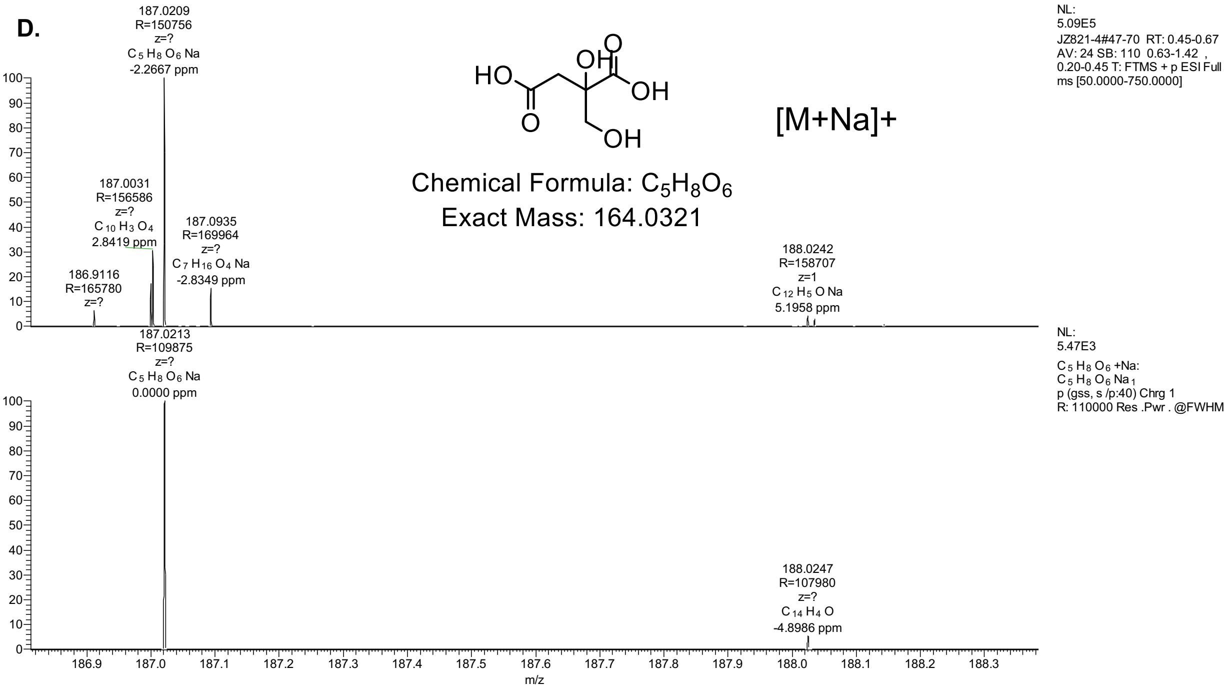

Figure S29. (A) Typical ^{1}H NMR spectrum (400 MHz, ns = 8, d1 = 30 s) for the experiments where **5a**[8] and **5b** were observed from the hydration of **1d** (pH 13-14 due to the addition of the thiolate/phosphate solution to precipitate metals). In this case, the ^{1}H NMR spectrum corresponds to Table S14-entry 1 with a zoom on the 2.2-3.8 ppm region. (B) ^{1}H NMR spectrum (400 MHz, ns = 16, d1 = 2 s) of Table S14-Entry 3 with a zoom on the 2.2-3.8 ppm region. In this case, only **5b** was observed. (C) Identification of **5a** by HRMS analysis. (D) Identification of **5b** by HRMS analysis.

E. Hydration of mesaconate (1e) to (6a)

Samples were prepared according to the general procedure **VI.A**. Samples were subsequently analyzed by mass spectroscopy to further confirm the formation of the products (**6a** and **6b**, Fig. S30B).

Table S15. Hydration of mesaconate analyzed by ^{1}H qNMR[a].

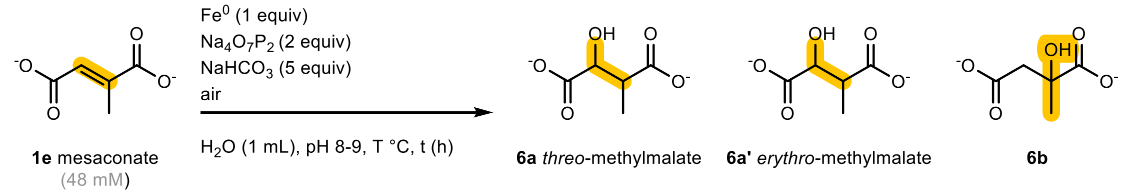

Entry	T° (°C)	Time (t, in hours)	Int. **1e** (1 H, 5.85 ppm)	Yield **1e** (%)	Int. **6a**[8] (3 H$_C$, 0.8 ppm)	Yield **6a** (%)	Int. **6a'**[8] (3 H$_C$, 0.95 ppm)	Yield **6a'** (%)	Int. **6b** (1 H$_A$, 2.22-2.3 ppm)	Yield **6b** (%)	**1e** (mM)	**DMS** (mM)[a]
**1**[b,c]	40	16	10.3	64.1	3.0	6.2	1.4	2.9	0.8	5.0	49.2	2.142
**2**[c]	75	16	15.4	79.0	3.6	6.2	1.8	3.1	0.9	4.6	55.8	2.142

[a] The yields were determined using 500 µL of the reaction mixture and, if not noted otherwise, 50 µL of a 23.6 mM DMS stock solution in D2O (total volume of NMR sample: 550 µL). Yields were calculated relative to dimethyl sulfone used as an internal standard (integral set to 6 H, concentration inside the NMR tube reported in the last column).

[b] To precipitate metals, 150 µL of the thiolate/phosphate solution was added to 500 µL instead of 700 µL of the reaction mixture.

[c] The products were quantified by quantitative ^{1}H NMR (d1 = 30 s, 8 scans), using 500 µL of the reaction mixture and, 50 µL of a 23.6 mM stock solution of DMS (DMS in D2O). The yields were determined relative to dimethyl sulfone used as an internal standard (integral set to 6 H, concentration inside the NMR tube reported in the last column). For the choice of protons to quantify the formation of the product, see Fig. S30.

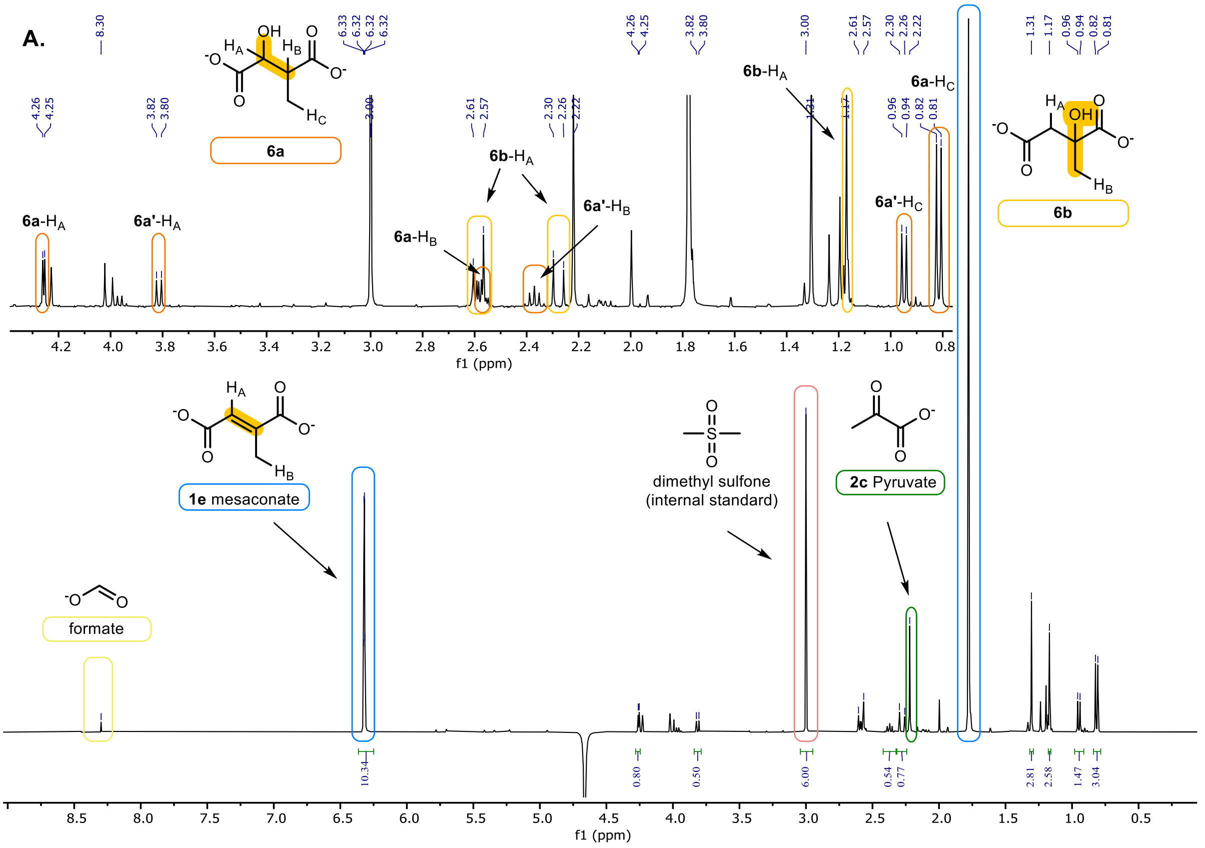

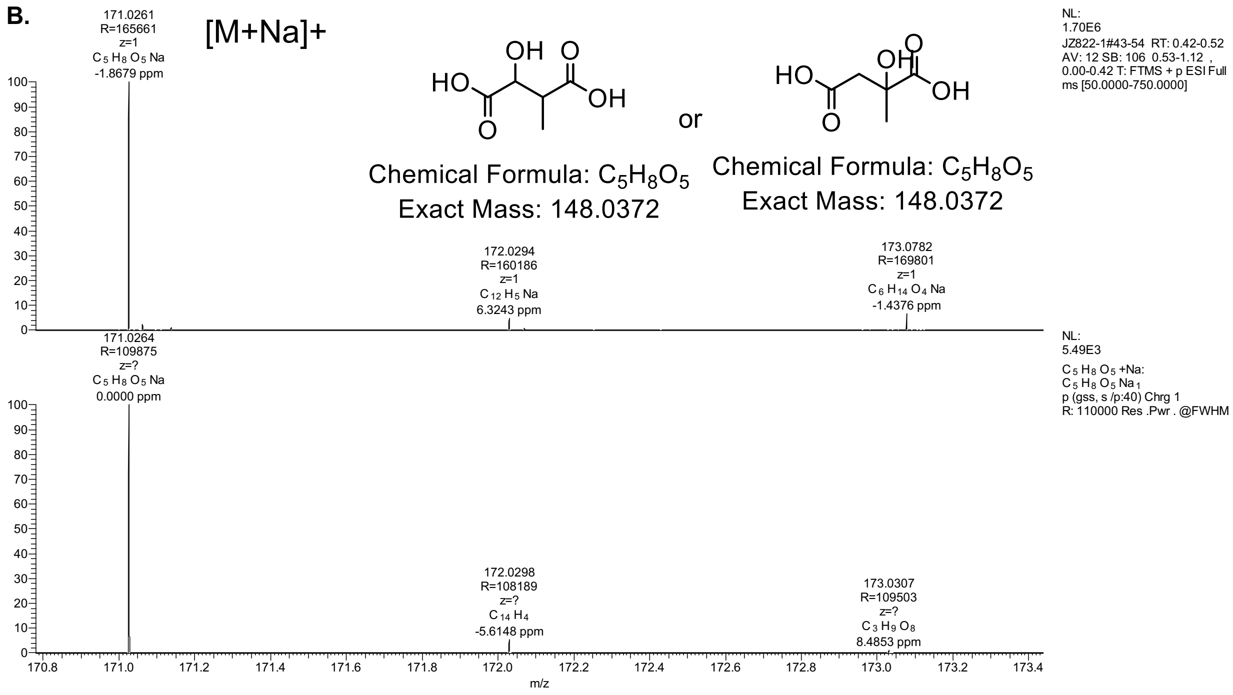

Figure S30. (A) Typical ^{1}H NMR spectrum (400 MHz, ns = 8, d1 = 30 s) for the experiments where **6a**[8] and **6b** were observed from the hydration of **1e** (pH 13-14 due to the addition of the thiolate/phosphate solution to precipitate metals). In this case, the ^{1}H NMR spectrum corresponds to Table S15-entry 1 with a zoom on the 0.8-4.3 ppm region. (B) Identification of isomers **6a** and/or **6b** by HRMS analysis.

F. Hydration of a non-conjugated alkene (1f)

Samples were prepared according to the general procedure **VI.A**.

Table S16. Hydration of **1f** analyzed by ^{1}H qNMR.

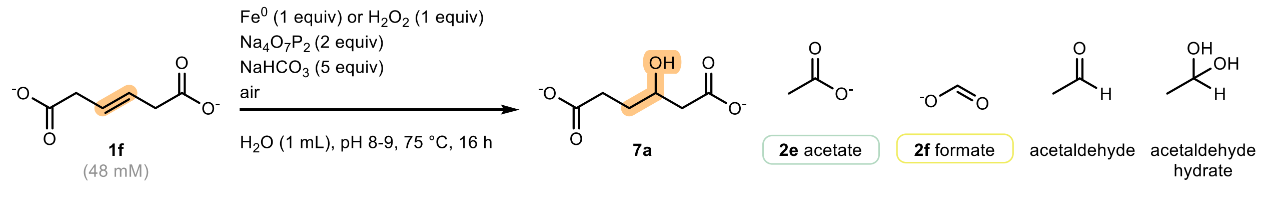

Entry	Reagent	7a	Observations	Standard	Conc. Standard (mM)
1	Fe^0	/	Formate, Acetate, Acetaldehyde, Acetaldehyde hydrate	DSS	1.849
2	H_2O_2	/	Formate, Acetate, Acetaldehyde	DSS	1.849

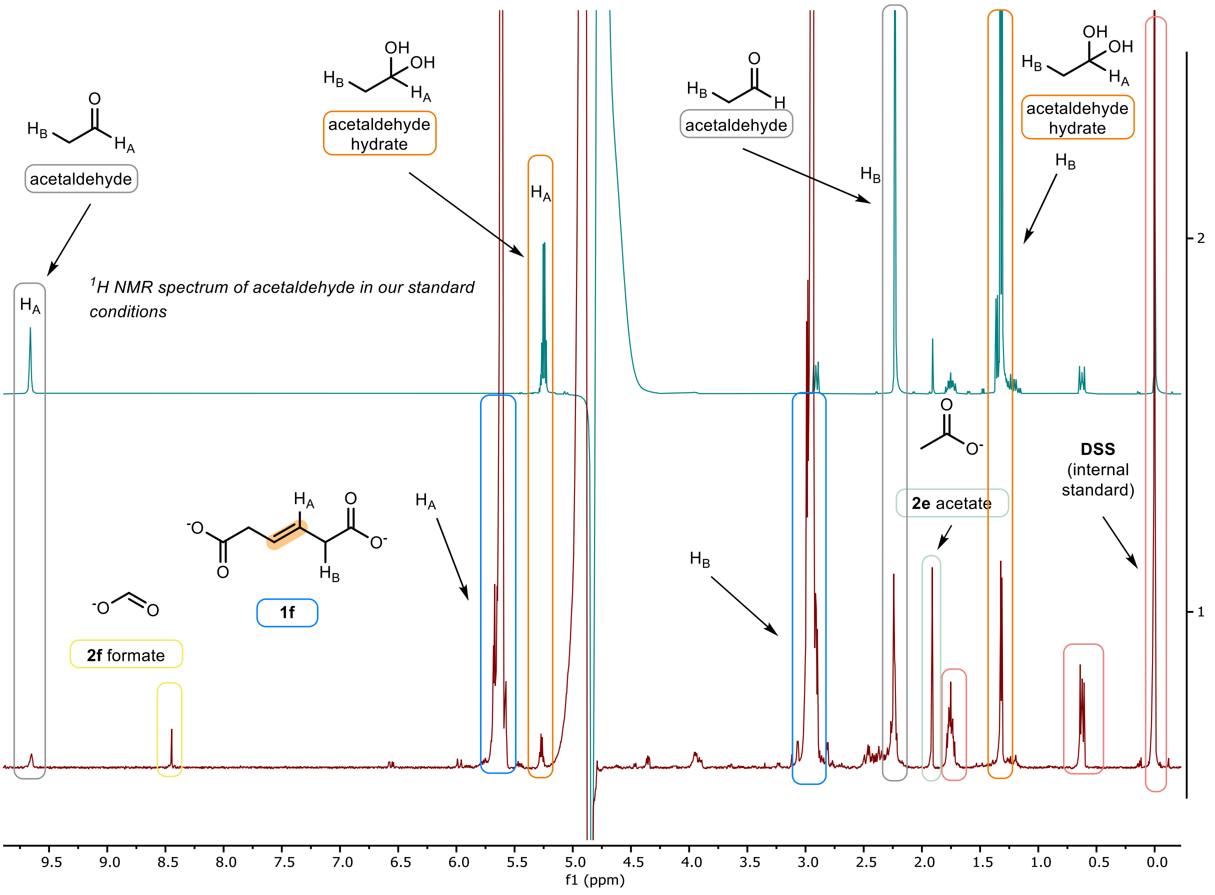

Figure S31. ^{1}H NMR spectrum (noesygppr1d, 400 MHz, ns = 16) of the reaction mixture obtained when **1f** was subjected to our standard conditions in which acetate (**2e**), formate (**2f**), acetaldehyde, and acetaldehyde hydrate were observed (pH 13-14 due to the addition of the thiolate/phosphate solution to precipitate metals). The spectrum is stacked with the commercially available product acetaldehyde in our standard conditions.

G. Hydration of all core metabolites (1a, 1b, and 1c) in one-pot

An aqueous stock solution of $Na_4O_7P_2$ (2 equiv, 25.5 mg) and $NaHCO_3$ (5 equiv, 20 mg) was freshly prepared in 1 mL of MQ H_2O for each reaction. The mixture was vortexed until the dissolution of the remaining $Na_4O_7P_2$ powder. Then, Eppendorf vials were charged with all substrates (**1a**, 10 mg; **1b**, 6.5 mg; **1c**, 8.6 mg, both 48 mM), Fe^0 (x equiv, see Table S17), and 1 mL of the freshly prepared stock solution was added. To perform the reaction under air without evaporation of the solution under heating, a needle was introduced into the lid of the Eppendorf vial (see Fig. S10A). Samples were placed on a pre-tempered thermoshaker, and incubated for 16 h at 75 °C with a speed of 800 rpm. After the indicated time, the **GP** for NMR sample preparation in the presence of metal catalysts was applied. The products were quantified by quantitative ^1H NMR (d1 = 30 s, 32 scans), using 500 µL of the reaction mixture, 50 µL of a 23.6 mM **DMS** stock solution in D_2O and 50 µL of a 77.8 mM phosphonoacetate stock solution in D_2O (total volume of NMR sample: 600 µL).

Table S17. One-pot hydration of **1a**, **1b**, and **1c** analyzed by ^1H qNMR (n.c. = observed but not detected).

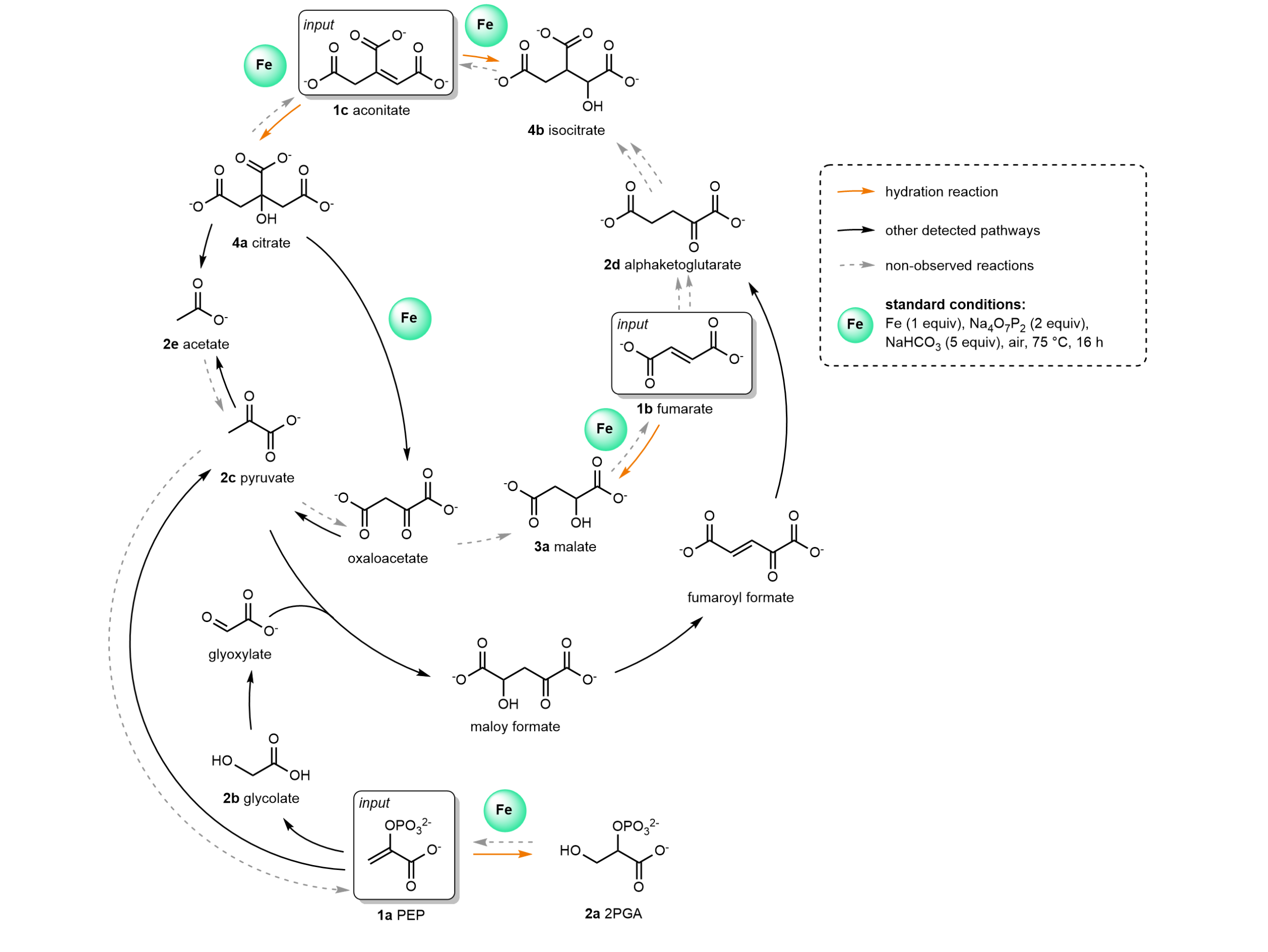

Entry	Fe⁰ (equiv)	/	Int. **2a** (1 H, H_B, 3.77-3.74 ppm)	Int. **2b** (2 H, 3.8 ppm)	Int. **2c** (3 H, 2.22 ppm)	Int. **3a** (1 H, H_A, 4.14-4.16 ppm)	[b]Int. **4a** (2 H, 2.53 and 2.49 ppm)	Int. **4b** (1 H_A, 3.84 ppm)	Int. **4b'** (1 H_A, 4.23 ppm)	**1a** (mM)	**1b** (mM)	**1c** (mM)	**DMS** (mM)
**1**[a]	1	Int.	0.8	3.7	9.4	0.8	0.3	0.5	0.4				
		C(mM)	1.57	3.63	6.15	1.57	0.29	0.98	0.79	47.8	47.8	47.7	1.963
		Yield (%)	4.8	11.1	n.c.	4.8	0.9	3.0	2.4				
**2**[c]	3	Int.	1.4	1.8	7.5	1.9	0.5	0.7	0.8				
		C(mM)	3.00	1.93	5.35	4.07	0.54	1.50	1.71	47.8	47.8	47.7	2.142
		Yield (%)	8.4	5.4	n.c.	11.4	1.5	4.2	4.8				

[a] Yields were calculated relative to dimethyl sulfone used as an internal standard (integral set to 6 H, concentration inside the NMR tube reported in the last column).

[b] To quantify the formation of **4a**, the deconvolution method "Auto Line Fitting" of the MestReNova software was used for the peaks of **4a** which were overlapping as represented in Fig. S32B.

[c] The yields were determined using 500 µL of the reaction mixture and 50 µL of a 23.6 mM DMS stock solution in D_2O (total volume of NMR sample: 550 µL). Yields were calculated relative to dimethyl sulfone used as an internal standard (integral set to 6 H, concentration inside the NMR tube reported in the last column).

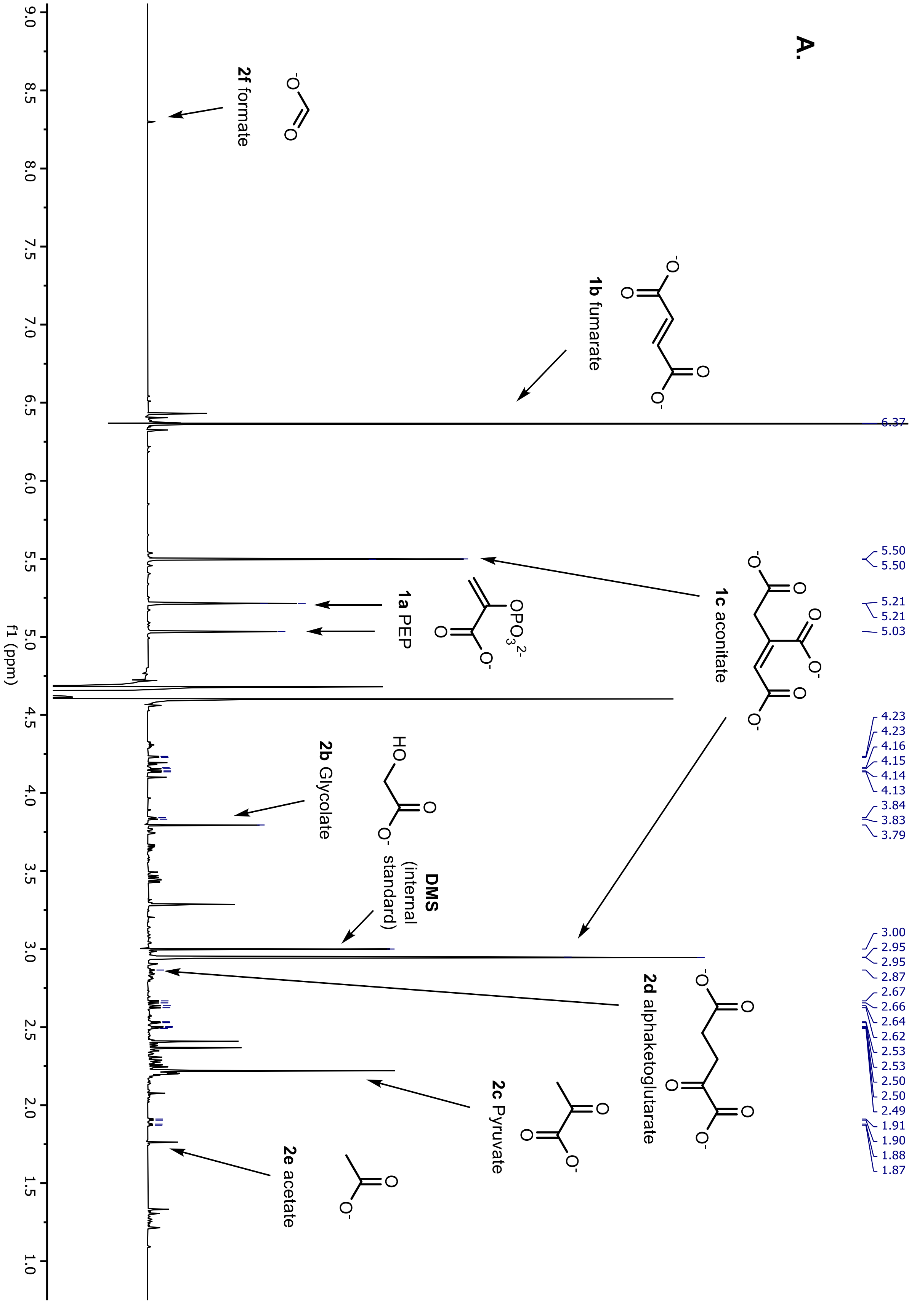

A.
2f formate
1b fumarate
1c aconitate
1a PEP
2b Glycolate
DMS (internal standard)
2d alphaketoglutarate
2c Pyruvate
2e acetate
f1 (ppm)

B.

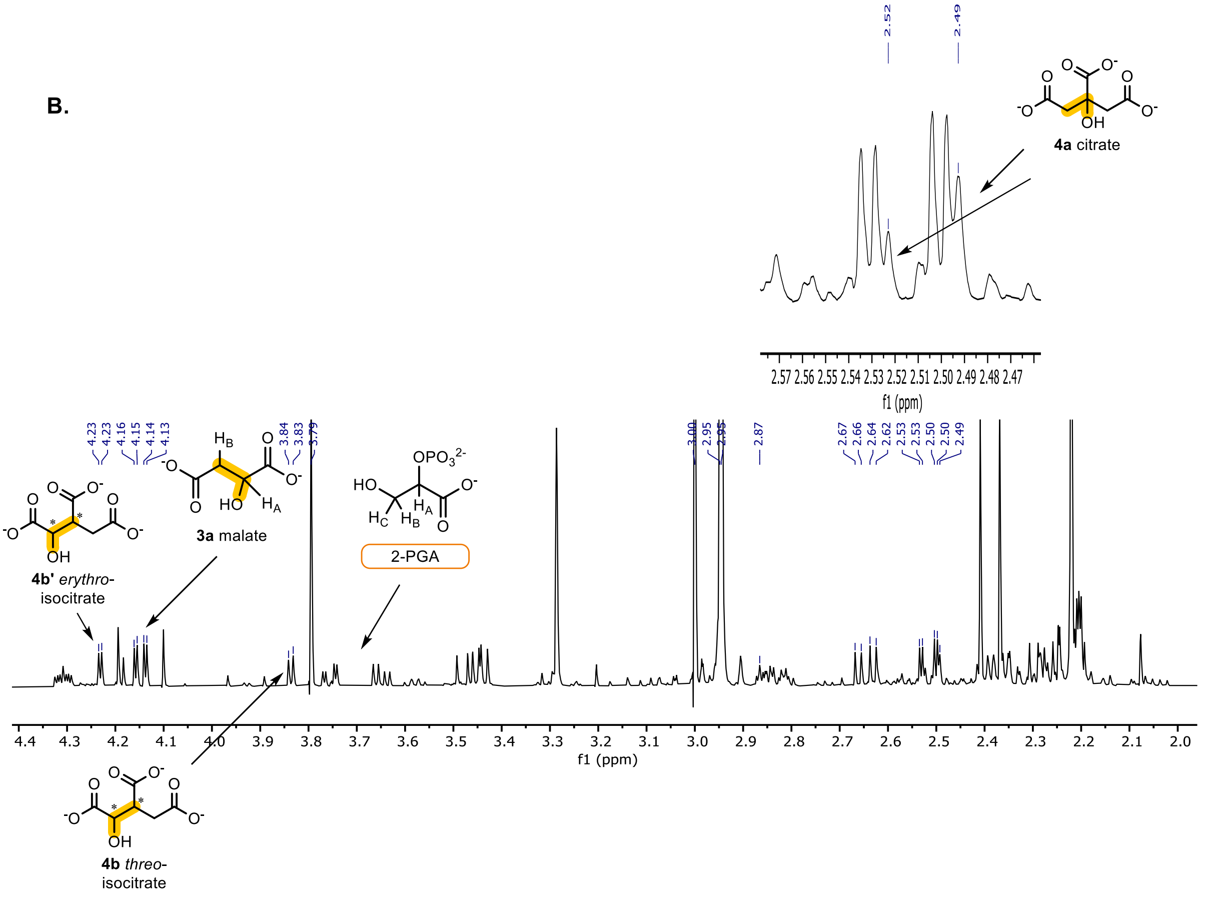

Figure S32. (A) ^{1}H NMR spectrum (500 MHz, ns = 32, d1 = 30 s) of the one-pot hydration of **1a**, **1b**, and **1c** (pH 13-14 due to the addition of the thiolate/phosphate solution to precipitate metals). (B) Zoom on the 2.0-4.4 ppm region showing which peaks were integrated for each product (i.e., **2-PGA**, **3a**, **4a**, **4b**, and **4b'**).

VII. Further investigations of the reaction mechanism

A. Investigation of kinetics of the reaction

1) General procedure for kinetics

An aqueous stock solution of **PEP** (48 mM), $Na_4O_7P_2$ (2 equiv), and $NaHCO_3$ (5 equiv) was freshly prepared for 12 reactions as follows: **PEP** (48 mM, 120 mg), $Na_4O_7P_2$ (2 equiv, 306 mg) and $NaHCO_3$ (5 equiv, 242 mg) were dissolved in 12 mL of MQ H_2O. After the addition of H_2O, gas bubbles were observed, and the mixture was vortexed until the dissolution of the remaining $Na_4O_7P_2$ powder. Then, Eppendorf vials were charged with the metal catalyst (x equiv, see below), and 1 mL of the freshly prepared stock solution was added to each reaction. To perform the reaction under air without evaporation of the solution under heating, a needle was introduced into the lid of the Eppendorf vial (Fig. S10A). Samples were placed on a pre-tempered thermoshaker and incubated for different reaction times at 75 °C (see Table S18) with a speed of 800 rpm. After the indicated time, the **GP** for NMR sample preparation in the presence of metal catalysts was applied. All NMR samples were analyzed by ^1H qNMR (d1 = 30 s, 8 scans).

2) Kinetics with variation of Fe^0 concentration

a) Kinetics using 1 equiv of Fe^0

Samples were prepared according to the general procedure **VII.A.1)** Eppendorf vials were charged with Fe^0 (1 equiv, 2.7 mg).

Table S18. Time variation with product quantification by ^1H qNMR.

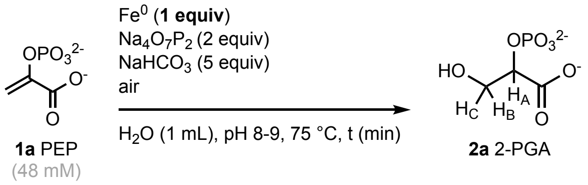

En-try	Time (min)	**1a** (1 H, 5.21 ppm[b])				moy	stdev	**2a** (1 H, HB, 3.77-3.74 ppm)				moy	stdev	**1a (mM)**	**DMS (mM)**
		Run 1		Run 2				Run 1		Run 2					
		Int.	Yield (%)	Int.	Yield (%)			Int.	Yield (%)	Int.	Yield (%)				
**1**	0	15.6	93.2	15.4	91.8	92.4	0.8	0.0	0.0	0.0	0.0	0.0	0.0	47.9	1.961
**2**	30	13.9	83.0	13.6	80.9	82.0	1.6	0.0	0.0	0.04	0.2	0.1	0.2	47.9	1.961
**3**	60	11.6	69.3	13.1	78.4	73.9	6.4	0.5	2.7	0.3	1.5	2.1	0.9	47.9	1.961
**4**	120	12.4	74.0	12.7	76.1	75.2	1.3	0.7	3.9	0.4	2.6	3.2	0.9	47.9	1.961
**5**	180	11.4	68.1	11.7	69.9	69.1	1.1	0.9	5.5	1.1	6.7	6.1	0.8	47.9	1.961
**6**	240	11.7	69.9	11.3	67.5	68.5	1.5	1.4	8.6	1.2	6.9	7.7	1.2	47.9	1.961
**7**	360	10.5	62.7	11.0	65.9	64.3	2.2	1.9	11.6	1.5	9.2	10.4	1.7	47.9	1.961
**8**	480	9.4	56.1	9.3	55.5	55.7	0.3	2.5	14.9	2.6	15.8	15.3	0.6	47.9	1.961
**9**	600	9.2	54.9	8.5	51.0	53.1	2.9	2.7	16.3	2.9	17.3	16.8	0.7	47.9	1.961
**10**	750	8.9	53.1	9.7	58.1	55.5	3.6	2.5	14.7	2.5	14.7	14.7	0.0	47.9	1.961
**11**	960	8.2	49.0	8.2	49.0	49.1	0.1	3.3	19.5	2.9	17.1	18.3	1.6	47.9	1.961
**12**	1440	8.3	49.6	8.5	51.0	50.3	0.9	3.2	19.3	2.3	13.8	16.6	3.9	47.9	1.961

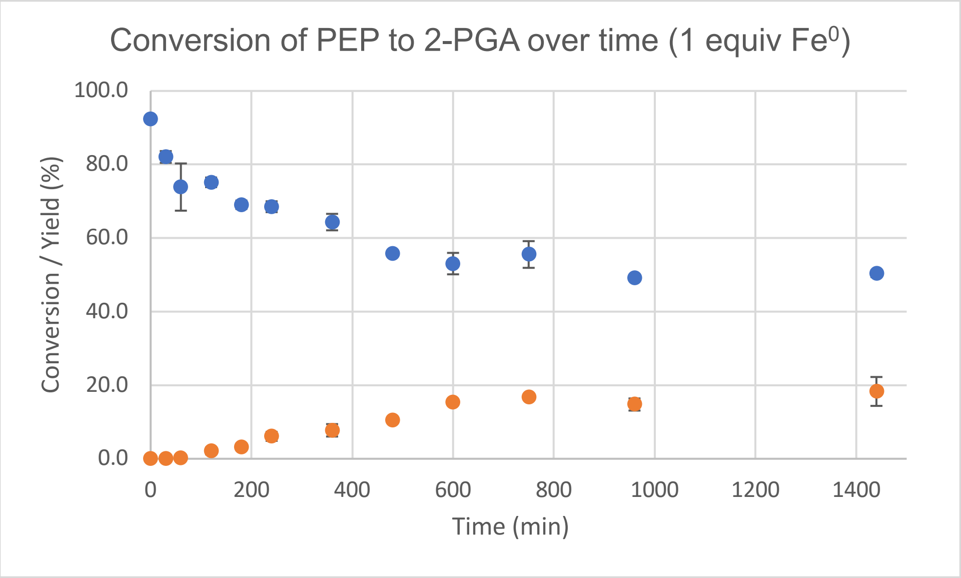

b) Kinetics using 0.5 equiv of Fe^0

Samples were prepared according to the general procedure **VII.A.1)** Eppendorf vials were charged with Fe^0 (0.5 equiv, 1.4 mg).

Table S19. Time variation with product quantification by ^1H qNMR.

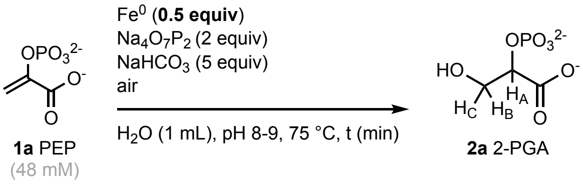

En-try	Time (min)	1a (1 H, 5.21 ppm[b])						2a (1 H, HB, 3.77-3.74 ppm)						1a (mM)	DMS (mM)
		Run 1		Run 2				Run 1		Run 2					
		Int.	Yield (%)	Int.	Yield (%)	moy	stdev	Int.	Yield (%)	Int.	Yield (%)	moy	stdev		
**1**	0	15.8	94.3	15.5	92.6	93.4	1.2	0.0	0.0	0.00	0.00	0.0	0.0	47.8	1.961
**2**	30	14.9	89.3	15.0	89.6	89.4	0.2	0.0	0.0	0.00	0.00	0.0	0.0	47.8	1.961
**3**	60	14.1	84.3	14.3	85.4	84.9	0.8	0.0	0.0	0.00	0.00	0.0	0.0	47.8	1.961
**4**	120	12.8	76.4	12.9	77.2	76.8	0.6	0.3	1.6	0.27	1.61	1.6	0.0	47.8	1.961
**5**	180	12.0	71.8	12.5	74.6	73.2	2.0	0.0	0.2	0.08	0.48	0.4	0.2	47.8	1.961
**6**	240	11.7	69.9	11.7	69.7	69.8	0.1	0.3	1.7	0.90	5.38	3.6	2.6	47.8	1.961
**7**	360	10.9	65.0	10.1	60.1	62.6	3.5	0.9	5.4	1.16	6.94	6.2	1.1	47.8	1.961
**8**	480	10.1	60.5	10.4	62.2	61.4	1.2	1.6	9.6	1.66	9.93	9.8	0.2	47.8	1.961
**9**	750	10.4	62.2	10.1	60.2	61.2	1.4	1.8	10.5	1.55	9.27	9.9	0.9	47.8	1.961
**10**	960	10.0	59.8	9.8	58.4	59.1	1.0	1.7	10.0	1.59	9.51	9.7	0.3	47.8	1.961
**11**	1440	9.4	56.2	10.1	60.5	58.3	3.0	1.3	8.0	1.53	9.15	8.6	0.8	47.8	1.961

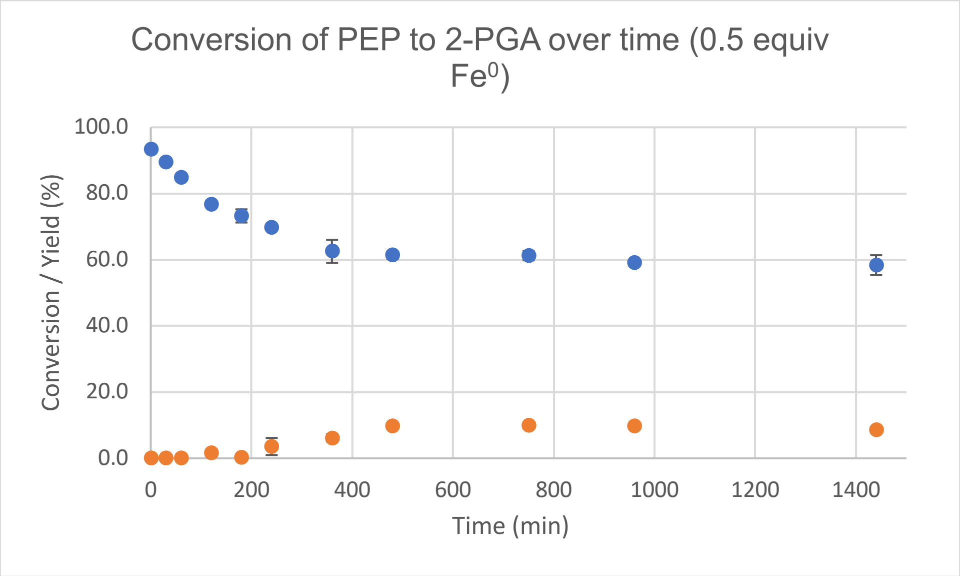

c) Kinetics with addition of 5% of products with 1 equiv. of Fe^0

Samples were prepared according to the general procedure **VII.A.1)** To the stock solution was added 5 % of products (i.e., **2a**, **2b**, and **2c**) was added to verify a potential catalyst deactivation effect. Eppendorf vials were charged with Fe^0 (1 equiv, 2.7 mg).

Table S20. Time variation with product quantification by ^{1}H qNMR.

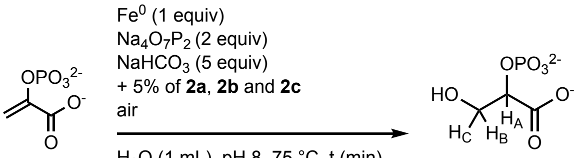

1a PEP (48 mM) → **2a** 2-PGA

Entry	Time (min)	**1a** (1 H, 5.21 ppm[b]) Run 1 Int.	Run 1 Yield (%)	Run 2 Int.	Run 2 Yield (%)	moy	stdev	**2a** (1 H, HB, 3.77-3.74 ppm) Run 1 Int.	Run 1 Yield (%)	Run 2 Int.	Run 2 Yield (%)	moy	stdev	**1a**	**DMS**
**1**	0	15.5	92.5	15.6	93.3	92.9	0.5	0.6	3.3	0.6	3.3	3.3	0.0	47.8	1.961
**2**	30	14.7	88.1	14.8	88.3	88.2	0.1	0.6	3.5	0.6	3.4	3.4	0.0	47.8	1.961
**3**	60	14.1	84.1	14.1	84.6	84.3	0.3	0.6	3.7	0.6	3.5	3.6	0.1	47.8	1.961
**4**	120	13.3	79.5	13.1	78.2	78.8	0.9	0.8	4.8	0.7	3.9	4.4	0.7	47.8	1.961
**5**	180	12.1	72.2	12.3	73.5	72.8	0.9	0.8	5.0	1.1	6.5	5.7	1.1	47.8	1.961
**6**	240	11.8	70.8	11.5	68.8	69.8	1.4	1.2	7.4	1.5	9.1	8.2	1.2	47.8	1.961
**7**	360	11.2	67.2	10.9	65.1	66.2	1.5	2.0	11.7	1.7	9.9	10.8	1.2	47.8	1.961
**8**	480	10.6	63.2	10.5	62.7	62.9	0.4	2.3	13.6	2.1	12.3	12.9	0.9	47.8	1.961
**9**	600	11.1	66.5	8.9	53.0	59.8	9.5	2.0	11.8	2.4	14.4	13.1	1.9	47.8	1.961
**10**	750	9.4	56.2	9.2	55.0	55.6	0.8	2.9	17.4	3.0	17.8	17.6	0.3	47.8	1.961
**11**	960	8.3	49.9	8.4	50.2	50.0	0.2	3.1	18.2	2.9	17.3	17.8	0.7	47.8	1.961
**12**	1440	9.0	53.9	8.3	49.7	51.8	3.0	2.6	15.4	3.0	17.8	16.6	1.7	47.8	1.961

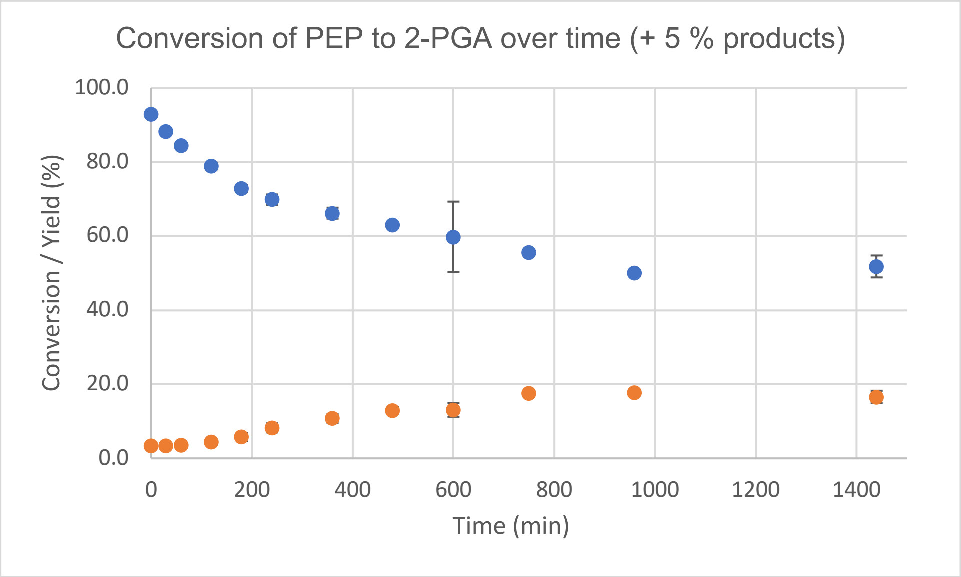

d) Comparison between kinetics in the presence and in the absence of 5% of product

Table S21. Comparison between kinetics in the presence and in the absence of 5% of product.

2PGA + 5% product				2PGA w/o 5% product		
**Time**	**[a]2PGA**	**[a]2PGA – 2PGA (t_0)**	**STD DEV**	**Time**	**[a]2PGA**	**STD DEV**
0	3,3	0	0	0	0	0
30	3,4	0,1	0	30	0,1	0,2
60	3,6	0,3	0,1	60	2,1	0,9
120	4,4	1,1	0,7	120	3,2	0,9
180	5,7	2,4	1,1	180	6,1	0,8
240	8,2	4,9	1,2	240	7,7	1,2
360	10,8	7,5	1,2	360	10,4	1,7
480	12,9	9,6	0,9	480	15,3	0,6
600	13,1	9,8	1,9	600	16,8	0,7
750	17,6	14,3	0,3	750	14,7	0
960	17,8	14,5	0,7	960	18,3	1,6
1440	16,6	13,3	1,7	1440	16,6	3,9

[a] Mean value for two runs.

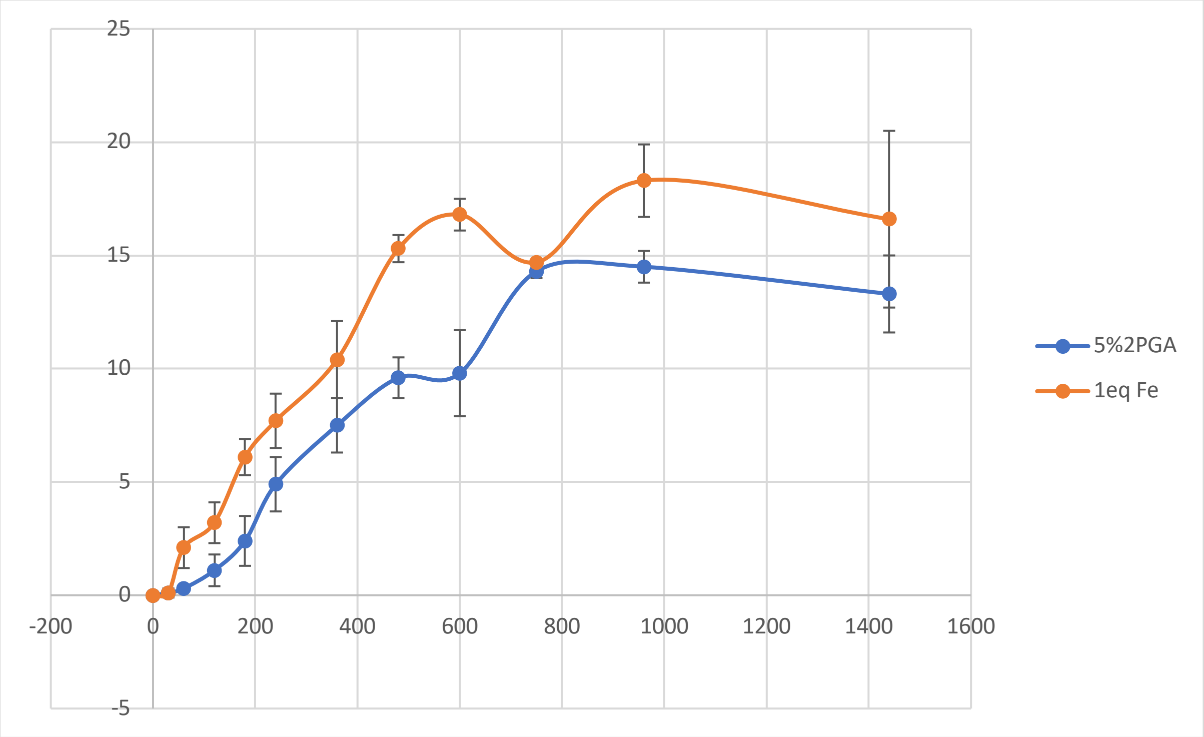

Figure S33. Comparison between kinetics in the presence and in the absence of 5% of product using 1 equivalent of Fe^0. In blue is represented the kinetic measurements in the presence of 5% of product at the beginning of the reaction at which we extracted the value obtained at t_0 (cf. 2PGA – 2PGA (t_0) column in Table S21). In orange is represented the kinetic measurements in the absence of 5% of product (cf. 2PGA w/o 5% product in Table S21).

B. Deuterium isotope effect

An aqueous stock solution of **PEP** (54 mM), $Na_4O_7P_2$ (2 equiv), and $NaHCO_3$ (5 equiv) was freshly prepared for 1 reaction as follows: **PEP** (54 mM, 11 mg), $Na_4O_7P_2$ (2 equiv, 25 mg) and $NaHCO_3$ (5 equiv, 20 mg) were dissolved in 0.95 mL of D_2O. After the addition of D_2O, gas bubbles were observed, and the mixture was vortexed until the dissolution of the remaining $Na_4O_7P_2$ powder. Then, the Eppendorf vial was charged with the iron catalyst (1 equiv, 2.7 mg), and 1 mL of the freshly prepared stock solution was added to the vial. To perform the reaction under air without evaporation of the solution under heating, a needle was introduced into the lid of the Eppendorf vial (Fig. S10A). The sample was placed on a pre-tempered thermoshaker and incubated for 16 h at 75 °C (see Table S21) with a speed of 800 rpm. After the indicated time, the **GP** for NMR sample preparation in the presence of metal catalysts was applied. The NMR sample was analyzed by ^1H qNMR (d1 = 30 s, 8 scans).

Table S22. Deterium isotope effect experiment analyzed by ^1H qNMR[a]

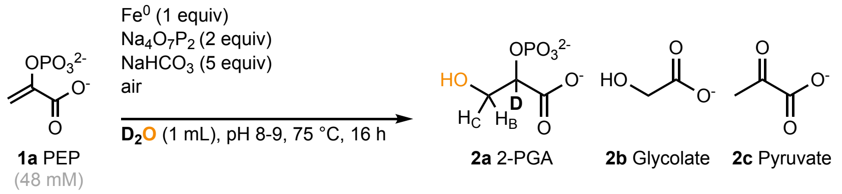

Entry	Int. **1a** (1 H, 5.21 ppm[b])	Yield **1a** (%)	Int. **2a** (1 H, H_B, 3.77-3.74 ppm)	Yield **2a** (%)	Int. **2b** (2 H, 3.8 ppm)	Yield **2b** (%)	Int. **2c** (3 H, 2.22 ppm)	Yield **2c** (%)	**PEP** (mM)	**DMS** (mM)[a]
**1**	8.6	45.3	1.8	9.5	6.3	16.6	0.4	0.7	54.3	1.964

[a] The yields were determined relative to dimethyl sulfone used as an internal standard (integral set to 6 H, concentration inside the NMR tube reported in the last column).

[b] The proton of **PEP** at 5.21 ppm was chosen for quantification to minimize the loss of integration due to the ^1H water suppression method (for details, see Fig. S11).

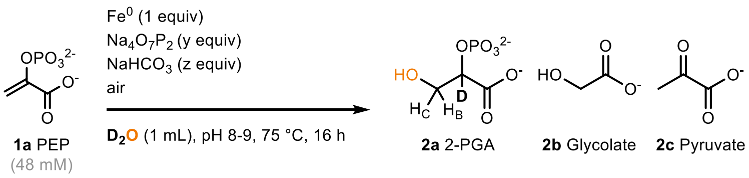

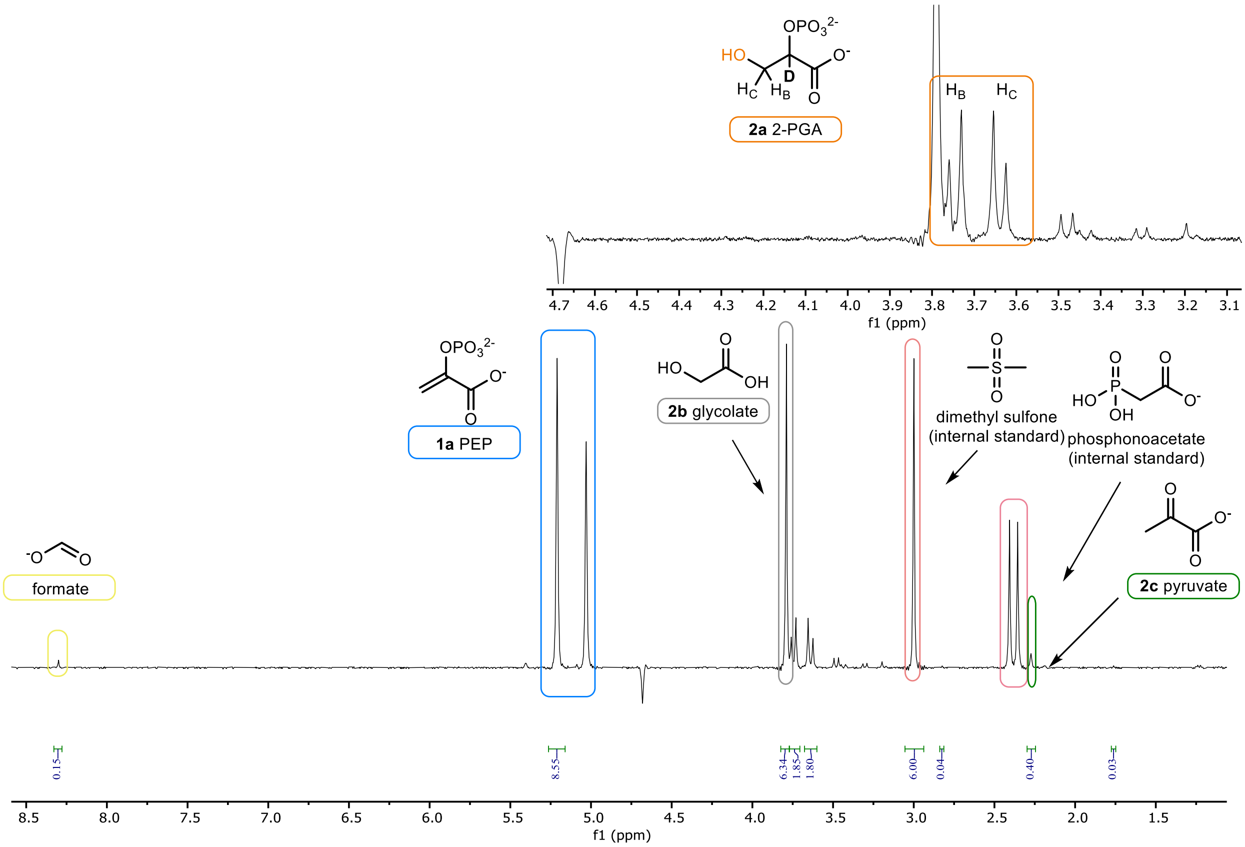

Figure S34. ^{1}H NMR spectrum (400 MHz, ns = 8, d1 = 30s) for the hydration of **1a** in D_2O. The proton (usually named H_A) of **2a** was exchanged with a deuterium atom. (pH 13-14 due to the addition of the thiolate/phosphate solution to precipitate metals).

C. Induction period experiment

An aqueous stock solution of $Na_4O_7P_2$ (2 equiv), and $NaHCO_3$ (5 equiv) was freshly prepared for 1 reaction as follows: $Na_4O_7P_2$ (2 equiv, 25 mg) and $NaHCO_3$ (5 equiv, 20 mg) were dissolved in 1 mL of H_2O. After the addition of H_2O, gas bubbles were observed, and the mixture was vortexed until the dissolution of the remaining $Na_4O_7P_2$ powder. Then, the Eppendorf vial was charged with the iron catalyst (1 equiv, 2.7 mg), and 1 mL of the freshly prepared stock solution was added to the vial. To perform the reaction under air without evaporation of the solution under heating, a needle was introduced into the lid of the Eppendorf vial (Fig. S10A). The sample was placed on a pre-tempered thermoshaker and incubated for 1h30 at 75 °C with a speed of 800 rpm. After the indicated time, **PEP** (48mM, 10 mg) was added to the reaction mixture and the reaction was run for an additional 30 min at 75 °C with a speed of 800 rpm. After the indicated time, the **GP** for NMR sample preparation in the presence of metal catalysts was applied. The NMR sample was analyzed by ^{1}H NMR (d1 = 2 s, 32 scans).

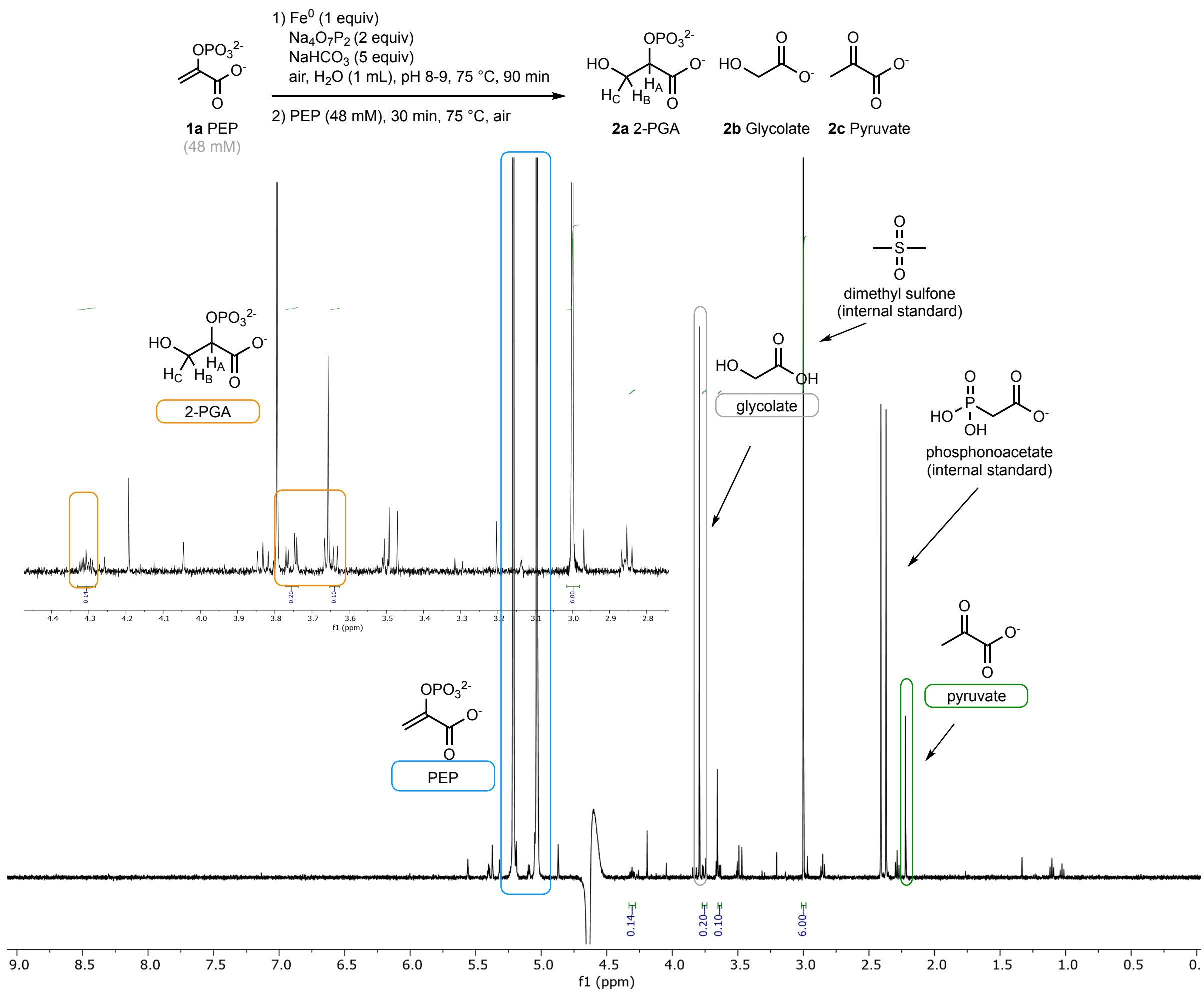

Figure S35. ^{1}H NMR spectrum (500 MHz, ns = 32, d1 = 2s) for the experiment with an induction period of 1h30 before the addition of **PEP** (**1a**) in the reaction mixture (pH 13-14 due to the addition of the thiolate/phosphate solution to precipitate metals). Only trace amounts of **2PGA** (**2a**) were observed after 30 min of reaction. Premixing the different reagents before adding the substrate did not avoid the induction period for the formation of **2a**.

References

[1] M. Yadav, S. Pulletikurti, J. R. Yerabolu, R. Krishnamurthy, *Nat. Chem.* **2022**, *14*, 170–178.
[2] Q. Dherbassy, R. J. Mayer, K. B. Muchowska, J. Moran, *J. Am. Chem. Soc.* **2023**, *145*, 13357–13370.
[3] K. B. Muchowska, S. J. Varma, J. Moran, *Nature* **2019**, *569*, 104–107.
[4] R. T. Stubbs, M. Yadav, R. Krishnamurthy, G. Springsteen, *Nat. Chem.* **2020**, *12*, 1016–1022.
[5] A. J. Coggins, M. W. Powner, *Nature Chem* **2017**, *9*, 310–317.
[6] H. Yao, D. E. Richardson, *J. Am. Chem. Soc.* **2000**, *122*, 3220–3221.
[7] O. Farr, N. Gaudu, G. Danger, M. J. Russell, D. Ferry, W. Nitschke, S. Duval, *Journal of The Royal Society Interface* **2023**, *20*, 20230386.
[8] J. Verduyckt, A. Geers, B. Claes, S. Eyley, C. V. Goethem, I. Stassen, S. Smolders, R. Ameloot, I. Vankelecom, W. Thielemans, D. E. D. Vos, *Green Chem.* **2017**, *19*, 4642–4650.
